# Easy and accurate reconstruction of whole HIV genomes from short-read sequence data with shiver

**DOI:** 10.1093/ve/vey007

**Published:** 2018-05-18

**Authors:** Chris Wymant, François Blanquart, Tanya Golubchik, Astrid Gall, Margreet Bakker, Daniela Bezemer, Nicholas J Croucher, Matthew Hall, Mariska Hillebregt, Swee Hoe Ong, Oliver Ratmann, Jan Albert, Norbert Bannert, Jacques Fellay, Katrien Fransen, Annabelle Gourlay, M Kate Grabowski, Barbara Gunsenheimer-Bartmeyer, Huldrych F Günthard, Pia Kivelä, Roger Kouyos, Oliver Laeyendecker, Kirsi Liitsola, Laurence Meyer, Kholoud Porter, Matti Ristola, Ard van Sighem, Ben Berkhout, Marion Cornelissen, Paul Kellam, Peter Reiss, Christophe Fraser, BEEHIVE Collaboration

**Affiliations:** 1 Big Data Institute, Li Ka Shing Centre for Health Information and Discovery, Nuffield Department of Medicine, University of Oxford, Oxford, UK; 2 Medical Research Council Centre for Outbreak Analysis and Modelling, Department of Infectious Disease Epidemiology, Imperial College London, London, UK; 3 Wellcome Centre for Human Genetics, Nuffield Department of Medicine, University of Oxford, Oxford, UK; 4 Department of Veterinary Medicine, University of Cambridge, Cambridge, UK; 5 Virus Genomics, Wellcome Sanger Institute, Wellcome Genome Campus, Hinxton, Cambridge, UK; 6 Laboratory of Experimental Virology, Department of Medical Microbiology, Center for Infection and Immunity Amsterdam (CINIMA), Academic Medical Center of the University of Amsterdam, Amsterdam, The Netherlands; 7 Stichting HIV Monitoring, Amsterdam, The Netherlands; 8 Department of Mathematics, Imperial College London, London, UK; 9 Department of Microbiology, Tumor and Cell Biology, Karolinska Institutet, Stockholm, Sweden; 10 Department of Clinical Microbiology, Karolinska University Hospital, Stockholm, Sweden; 11 Division for HIV and Other Retroviruses, Robert Koch Institute, Berlin, Germany; 12 School of Life Sciences, Ecole Polytechnique Fédérale de Lausanne, Lausanne, Switzerland; 13 Swiss Institute of Bioinformatics, Lausanne, Switzerland; 14 HIV/STI Reference Laboratory, Department of Clinical Science, WHO Collaborating Centre, Institute of Tropical Medicine, Antwerpen, Belgium; 15 Institute for Global Health, University College London, London, UK; 16 Department of Population Health, Faculty of Epidemiology and Population Health, London School of Hygiene and Tropical Medicine, London, UK; 17 Department of Pathology, John Hopkins University, Baltimore, MD, USA; 18 Department of Infectious Disease Epidemiology, Robert Koch-Institute, Berlin, Germany; 19 Division of Infectious Diseases and Hospital Epidemiology, University Hospital Zurich, Zurich, Switzerland; 20 Institute of Medical Virology, University of Zurich, Zurich, Switzerland; 21 Department of Infectious Diseases, Helsinki University Hospital, Helsinki, Finland; 22 Division of Intramural Research, NIAID, NIH, Baltimore, MD, USA; 23 INSERM CESP U1018, Université Paris Sud, Université Paris Saclay, APHP, Service de Santé Publique, Hôpital de Bicêtre, Le Kremlin-Bicêtre, France; 24 Kymab Ltd, Cambridge, UK; 25 Division of Infectious Diseases, Department of Medicine, Imperial College London, London, UK; 26 Department of Global Health, Academic Medical Center and Amsterdam Institute for Global Health and Development, Amsterdam, The Netherlands

**Keywords:** bioinformatics, next-generation sequencing, HIV, diversity, genome assembly, mapping

## Abstract

Studying the evolution of viruses and their molecular epidemiology relies on accurate viral sequence data, so that small differences between similar viruses can be meaningfully interpreted. Despite its higher throughput and more detailed minority variant data, next-generation sequencing has yet to be widely adopted for HIV. The difficulty of accurately reconstructing the consensus sequence of a quasispecies from reads (short fragments of DNA) in the presence of large between- and within-host diversity, including frequent indels, may have presented a barrier. In particular, mapping (aligning) reads to a reference sequence leads to biased loss of information; this bias can distort epidemiological and evolutionary conclusions. *De novo* assembly avoids this bias by aligning the reads to themselves, producing a set of sequences called contigs. However contigs provide only a partial summary of the reads, misassembly may result in their having an incorrect structure, and no information is available at parts of the genome where contigs could not be assembled. To address these problems we developed the tool shiver to pre-process reads for quality and contamination, then map them to a reference tailored to the sample using corrected contigs supplemented with the user’s choice of existing reference sequences. Run with two commands per sample, it can easily be used for large heterogeneous data sets. We used shiver to reconstruct the consensus sequence and minority variant information from paired-end short-read whole-genome data produced with the Illumina platform, for sixty-five existing publicly available samples and fifty new samples. We show the systematic superiority of mapping to shiver’s constructed reference compared with mapping the same reads to the closest of 3,249 real references: median values of 13 bases called differently and more accurately, 0 bases called differently and less accurately, and 205 bases of missing sequence recovered. We also successfully applied shiver to whole-genome samples of Hepatitis C Virus and Respiratory Syncytial Virus. shiver is publicly available from https://github.com/ChrisHIV/shiver.

## 1. Introduction

The genetic sequences of pathogens are a rich data source for studying their epidemiology and evolution, and provide information for vaccine and therapeutic design. In the past decade, next-generation sequencing (NGS) has transformed genomics, with decreasing costs and enormous increases in the amount of data available. Despite the success of NGS in other fields, sequencing of human immunodeficiency virus (HIV) is still largely based on the older method of Sanger sequencing. For example, on the comprehensive Los Alamos National Laboratory HIV database (http://www.hiv.lanl.gov/ accessed 11 October 2017), of the 147,751 samples with platform information, 90.8% were generated by Sanger sequencing, 6.9% with the Roche 454 platform, 2.2% with Illumina platforms, and 0.02% with the IonTorrent platform. Breakdowns of these numbers by date and sequence length are in Supplementary Section S1.

More broadly, NGS has been hugely successful both for sequencing samples with no within-sample diversity, and at the opposite end of the spectrum, for metagenomic studies. In the first case, any apparent within-sample diversity is attributable to sequencing error; in the latter case, there is no presumption that different fragments of sequence in the same sample have the same origin, and so each fragment is checked against large databases to catalogue these diverse origins (Kunin et al. 2008; Thomas, Gilbert, and Meyer, 2012).

**Figure 1.**
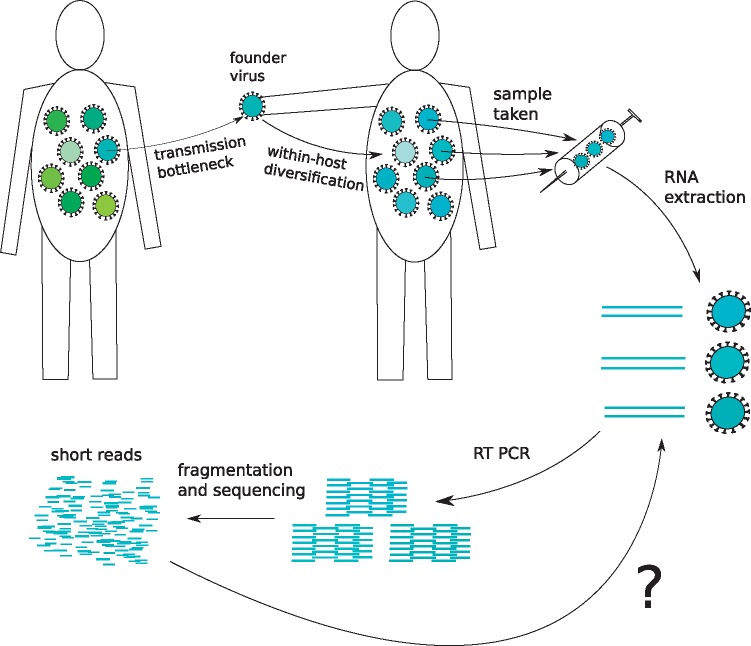
Interpreting NGS data for HIV. The question mark shows our question here: how best to discover the viral genotype from NGS data despite the high diversity of HIV between and within hosts?

HIV is an intermediate case: the long duration of chronic infection coupled with high rates of replication and mutation mean that a single infection, and hence a single sample, will contain a diverse collection of related viral particles, frequently called a quasispecies. The long generation time for HIV transmission, together with continual within-host evolution, results in large, star-like phylogenies at the between-host level (Grenfell et al. 2004), i.e. each individual’s quasispecies is quite distinct from the quasispecies of others. Reconstructing different aspects of these diverse quasispecies from *reads* (fragments of sequence; see Fig. 1) has proven technically challenging (Beerenwinkel et al. 2012) and may have hindered the widespread adoption of NGS for HIV. The complications of working with reads derived from a quasispecies can be bypassed with single genome amplification (SGA): in SGA, by limiting dilution, samples are reduced to single-virion aliquots that are sequenced separately (Simmonds et al. 1990; Palmer et al. 2005; Keele et al. 2008). However, the costs of using SGA for large population studies may be prohibitively high.

Here, we present the user-friendly programme shiver for working with HIV NGS data. Note that a variety of NGS platforms exist, which can be broadly classified into short-read-low-error platforms and long-read-high-error platforms (see e.g. Goodwin, McPherson, and McCombie, 2016); here we focus on the former. Our programme was developed as part of the BEEHIVE project (*Bridging the Evolution and Epidemiology of HIV in Europe*) in which samples from over 3,000 individuals with known date of HIV infection are being sequenced to investigate the viral-molecular basis of virulence (Fraser et al. 2014). The power of genome-wide association studies (GWASs), and of epidemiological analyses e.g. identifying transmission risk factors, is enhanced by focussing resources on the widest possible population coverage (and so use of SGA is not a priority). We explain the need for shiver in the following subsection.

### 1.1 Mapping reads: problems and solutions

The quasispecies in one infected individual can be summarised by the consensus sequence—the ‘average’ sequence of those virions sampled, as represented in the reads. Determining the most common base at each position in the genome, and which other bases are present and at what frequencies, requires the reads to be *mapped* (aligned) to a reference sequence. To what should they be mapped? Mapping to a reference too far from the quasispecies’ true consensus leads to biased loss of information (Archer et al. 2010; Henn et al. 2012; Iqbal et al. 2012; McElroy, Thomas, and Luciani, 2014). Like any form of sequence alignment, mapping relies upon sequence similarity; the more a read differs from its reference, the less likely it is to be aligned correctly or at all. This tends to hide differences between the sample and the reference, giving a consensus genome erroneously similar to the reference chosen.

The implications of this problem for downstream sequence analysis are worrying. Using the same reference for multiple infected individuals will tend to make their consensuses artefactually similar, overestimating proximity in a transmission network and distorting epidemiological conclusions. Using old reference sequences to construct new ones biases the new to resemble the old, which could distort our picture of evolution and hinder monitoring of emerging virulent or resistant variants. As an example, in a survey of *env* gene diversity in currently circulating viruses for vaccine design, it would be highly undesirable to artificially bias the reconstructed sequence towards similarity with the standard HXB2 reference virus isolated in 1983.

**Figure 2.**
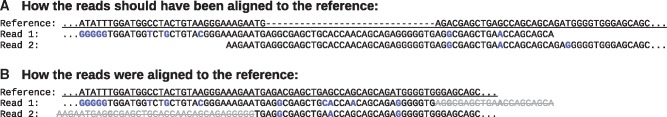
An example of biased loss of information encountered in our data when mapping to an existing reference. The reads contain a 30 bp insertion relative to the reference. Correct alignment, shown in the upper panel, would have inserted a 30 bp gap into the reference to accommodate this. What the mapper actually did (lower panel) was to align part of each read correctly either to the left of the insertion or to the right of it, and discard the rest of the read. ‘Read 1’ and ‘Read 2’ each represent roughly 2,000 similar reads; their consensus is therefore well supported but misses the insertion. This bias occurred despite the reference having been identified as the closest of 3,249 to this set of reads. Similar errors were made by the mapper’s smalt, BWA, and bowtie, resulting in the same erroneous consensus being called in each case. Bases in the reads that differ from the reference are shown in blue; the ends of the reads that were discarded during mapping (i.e. not aligned) are shown in grey with strikethrough. This figure corresponds to Position 8450 in Fig. 5.

**Figure 3.**
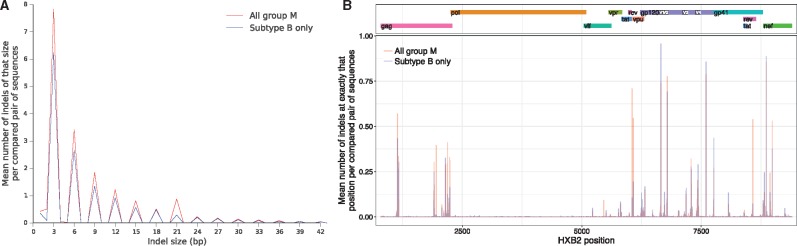
Quantifying indels in 3,249 whole genomes—those in the 2016 ‘all genome’ group M alignment from the Los Alamos National Laboratory HIV database. We trimmed both 3’ and 5’ ends of the alignment where sequences align poorly, then considered each of the roughly 5.3 million possible pairs of references therein. For each pair we calculated the size and position of their relative indels (i.e. taking their relative alignment from the overall alignment, ignoring positions at which both have a gap). We also considered just the subset of 1,019 subtype B sequences, which is less diverse than group M as a whole but shows similar indel patterns. Left panel: the distribution of indel sizes. The striking bias towards frame-preserving indels could be biological (frame-shifting indels will generally have a large fitness cost), artefactual (removal of frame-shifting indels from sequences during analysis before public release, on the assumption that this is sequencing or bioinformatic error), or a combination of both. Right panel: where in the genome the indels tend to occur. The observed pattern is consistent with purifying selection in pol and diversifying selection in env.

An example of this biased data loss is shown in Fig. 2, in which an insertion in the sample is lost because it is missing in the reference to which the reads were mapped. Reads containing insertions/deletions (indels) are particularly difficult to map correctly (Li, Ruan, and Durbin, 2008; Ye et al. 2009; McKenna et al. 2010; Albers et al. 2011). Inaccurate mapping at the sites of indels does not only result in missing the indel, as here, but can also prevent any reads from being mapped, or cause bases to be called incorrectly due to misalignment. This is an important point: even if the bases in an insertion are considered uninformative and are excluded from a particular comparative analysis, for example phylogenetic inference, it is undesirable that the insertion should cause missing or incorrect bases at neighbouring sites. Indels are known to be very common in HIV (Wood et al. 2009), especially in the *env* gene (Starcich et al. 1986). To quantify this further, we calculated indel size and position distributions in 3,249 whole genomes from the Los Alamos National Laboratory HIV database, shown in Fig. 3.

The loss of reads during mapping has been shown to be roughly proportional to the divergence between the true consensus and the reference used (Archer et al. 2010). The bias in the loss of reads (and the loss of accuracy in their alignment) occurs at different scales. Data are more likely to be lost in (1) those samples in a dataset that differ more greatly from the reference used for their mapping; (2) those parts of the genome, in a single sample, where the sample and reference are most different; and (3) a subset of genotypes, in a single diverse sample, that are more different from the reference than the other genotypes.

This problem means the simplest mapping strategy—using as a reference some existing, standard genome, even if chosen specifically for each sample based on the reads—has much room for improvement. For example one could map once to a standard reference, call the consensus, then use this as the reference for one or more rounds of remapping (Willerth et al. 2010; Gibson et al. 2014; McElroy, Thomas, and Luciani, 2014; Verbist et al. 2014; Ode et al. 2015). Remapping is expected to be more accurate, because the consensus initially called is expected to be closer to the true consensus than the standard reference is. For this to be the case all along the genome however, reads must map correctly all along the genome in the first step. If the sample has an indel not present in the reference, inaccurate mapping at the site of the indel may cause it to be missed when the consensus is called, as in Fig. 2. Remapping is then doomed to repeat the same error.

To correct for this, between initial mapping to the standard reference and calling the first consensus, multiple sequence alignment can be performed with the reads (Archer et al. 2010; Zanini et al. 2015). This removes some of the bias imposed by the initial mapping, because while mapping aligns each read to the reference sequentially and independently, multiple sequence alignment with the reads considers how the reads align to each other. It is then less important that reads map *correctly* all along the genome, since realignment may correct misalignment around indels, but the reads do still need to *map* all along the genome. If biased data loss leads to a failure of reads to map at a given point, the missing reads will not shape the initial consensus and remapping to that consensus will not recover them. For the variable loop regions of HIV’s *env* gene in particular, reads from one virus can easily fail to map to another; many examples of this can be seen in Supplementary Sections S4 and S5, visible as parts of the genome where reads do map to a reference tailored to the sample, but not to the closest identified real reference, resulting in missing sequence in the latter case.

These problems motivate *de novo* assembly (hereafter just assembly). Roughly, this consists of aligning overlapping reads to each other, tolerating some pre-set level of disagreement between them to allow for some within-sample diversity or sequencing error, iteratively extending using reads overhanging the edges, finally resulting in a set of sequences called contigs (see e.g. http://en.wikipedia.org/wiki/Sequence_assembly). Remapping to contigs (Henn et al. 2012; Yang et al. 2012; Malboeuf et al. 2013; McElroy, Thomas, and Luciani, 2014; Ode et al. 2015) settles ambiguity at positions spanned by multiple contigs which disagree, corrects positions where assembly did not call the most common base, provides minority variant information, and allows greater use to be made of base quality information than is typically done during assembly.

However, contigs may differ from the true consensus by more than just a few SNPs that can be corrected by mapping. Misassembly may occur, giving contigs supported by a high depth of reads but whose structure is very different from the known genome. This can arise *in silico* (McElroy, Thomas, and Luciani, 2014), i.e. by misassembly of correct reads; or as a result of chimeric reads produced during sequencing, due to recombination during library preparation (Meyerhans, Vartanian, and Wain-Hobson, 1990; Judo, Wedel, and Wilson, 1998; McElroy, Thomas, and Luciani, 2014), concatemerisation/ligation (Croucher et al. 2009), or stem loops of RNA secondary structure (Malboeuf et al. 2013).

Furthermore, the set of contigs resulting from assembly may not fully cover the genome. Gaps between contigs can be due to a total absence of reads there, following sequencing failure or only a partial genome present in the sample. They can also be due to the reads being too few (though non-zero), or too diverse, for successful assembly; in this case, mapping can recover consensus sequence not present in assembly output.

Finally, as the set of reads will generally contain contamination, so will the set of contigs. These contigs should be identified and discarded.

To address these problems we developed the tool shiver—*Sequences from HIV Easily Reconstructed—*to preprocess and map reads from each sample to a custom reference, tailored to be as close as possible to the expected consensus, constructed by correcting contigs and filling in gaps between them with the closest identified existing reference sequences. We wrote it to be easy to use, suitable for simple scripted application to large heterogeneous data sets, in our population genomics study and elsewhere.

## 2. Methods

### 2.1 A summary of the shiver method

**Figure 4.**
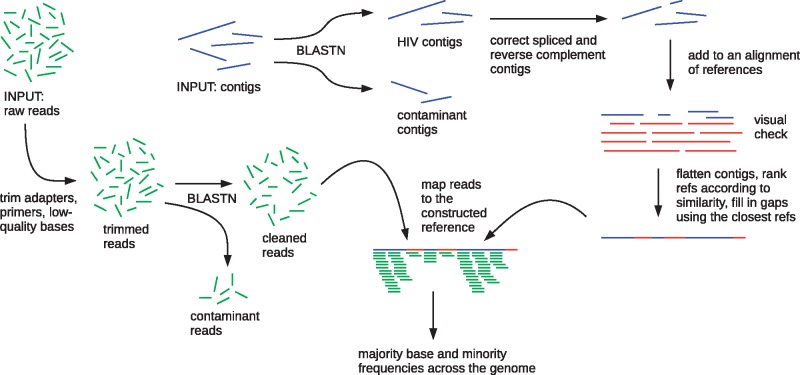
A summary of the steps in our method shiver.

The steps in shiver are shown in Fig. 4; see Supplementary Section S2 for more details.

In summary: paired-end short reads and contigs assembled from those reads are required as input for each sample; also required is a set of existing reference genomes, chosen by the user. Contigs are compared with the existing references using BLASTN (Altschul et al. 1990), then partitioned into those judged to be HIV and those judged to be contamination. HIV contigs are corrected as follows. First, spliced contigs—those concatenating two separated regions of the genome into a single sequence—are cut. The motivation for this cutting of contigs is the assumption that HIV does not exhibit major structural variation, e.g. variation in gene presence/absence or gene order, which is supported by sequence compendiums to date (http://www.hiv.lanl.gov/). Second, parts of contigs that did not have a blast hit to any existing reference are removed. Third, any contig (or part of a contig) found to be in the opposite orientation to the existing references is reverse-complemented. The contigs are added to the alignment of existing references using MAFFT (Katoh et al. 2002), and contigs found to have an overly large internal deletion are split into two separate contigs at that point.

At this point shiver stops to allow a visual check of the alignment of contigs and existing references. Once it is checked, shiver continues (all remaining steps in the programme are performed by the second of two commands needed for full processing). From this alignment, the closest existing reference is identified by comparison with all of the contigs. This is expected to be a more accurate identification of the closest existing reference than, for example, finding which existing reference most reads match most closely, which gives undue weighting to regions of the genome where more rounds of amplification resulted in an exponentially greater number of reads. shiver creates a reference for mapping by using contig sequence where available, and the closest existing reference to fill in any gaps between contigs (at parts of the genome where assembly failed). Before mapping, reads are trimmed for low-quality bases, adapter and primer sequences using Trimmomatic (Bolger, Lohse, and Usadel, 2014) and fastaq (https://github.com/sanger-pathogens/Fastaq); contaminant read pairs are diagnosed as those matching contaminant contigs more closely than the tailored reference, and are removed. The remaining reads are mapped to the tailored reference. By default we map using smalt with a minimum read identity (the fractional agreement between a read and the reference to be considered mapped) of 70%, independent mapping of mates in a pair, a maximum insert size of 2,000 bp, and discarding improperly paired reads. Optionally, BWA (Li and Durbin 2010) and bowtie (Langmead et al. 2009) can be used instead of smalt.

Following mapping, each position in the genome is considered in turn using SAMtools (Li et al. 2009), to find the frequencies of different bases. At positions where some reads have deletions relative to the mapping reference, we count the frequency of the gap character together with actual bases. At positions where some reads have insertions relative to the mapping reference, for the consensus we use the most common insertion size (which may be 0, i.e. no insertion). By default the most common base is called to give the consensus; optionally ambiguity codes can be used more readily, when the frequency of the most common base(s) is below a threshold. A consensus base is only called if the coverage equals or exceeds a minimum threshold specified by the user, to protect against the effect of residual low-coverage contaminant reads in genomic regions lacking genuine HIV reads. By default this is 15, but this is likely to need adjusting for different datasets. A tool contained in shiver helps the user to explore appropriate values (see the discussion of LinkIdentityToCoverage.py in Supplementary Section S3).

**Figure 5.**
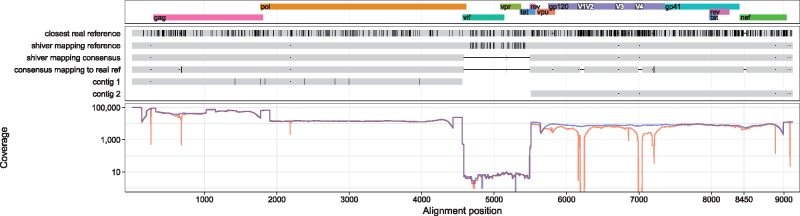
Top panel: HIV genes in their reading frames. Middle panel: sequences for the Miseq sample ERR732065. From top to bottom these are the closest identified real reference (see main text), the reference created and used for mapping by shiver, the consensus of reads mapped to shiver reference, the consensus of the same reads mapped to the real reference, and the contigs generated by *de novo* assembly. Vertical black lines inside sequences in the alignment denote single nucleotide polymorphisms (SNPs), defined here relative to the most common base among these sequences. Horizontal black lines indicate a lack of bases, i.e. a deletion relative to another sequence in the alignment or, for the two consensuses, simply missing sequence due to insufficient coverage. Bottom panel: the coverage (number of mapped reads) for the shiver reference in blue, and for the real reference in red. Mapping problems at Position 8450 are shown in detail in Fig. 2. Where the real reference and the sample differ by many close SNPs or an indel, differences often arise between the shiver consensus and the consensuses mapping to the real reference. The coverage plot beneath the sequences shows that at such points, the coverage mapping to the real reference almost always drops below the coverage mapping to the shiver reference; given that the same reads are being mapped to the same part of the genome with the same mapping parameters, this strongly suggests that the shiver consensus is more accurate. This is the case at Position 8450 in this figure, in the *nef* gene; the problem mapping to the real reference here was shown in detail in Fig. 2. Though the coverage here drops due to the problem aligning the reads, it is still more than 4,000, showing that a large absolute number of reads is no guarantee of accuracy. Mapping to the shiver reference on the other hand, coverage remains locally smooth. Similar errors mapping to the real reference in this figure can be seen in *gag* and in five different places in gp120.

By default, once the consensus is called, the cleaned reads are re-mapped to it (with any missing coverage in the consensus filled in with the corresponding part of the original tailored reference) for a second iteration of calling the base frequencies and the consensus. (This is why the shiver reference does not match the contigs exactly in Fig. 5 and the figures of Supplementary Sections S4 and S5).

shiver also produces a ‘global alignment’ of all consensuses it generates by coordinate translation, without need for an alignment algorithm.

### 2.2 Running shiver fully automatically

Alternatively shiver can be run from beginning to end without the break in the middle described above, for applications where visually checking the contigs is impractical. This is only possible for samples not requiring contig correction, and does not produce the global alignment of all samples’ consensuses together. The different alignment strategy used in this case, and our recommendation that the contigs be checked instead, are discussed further in Supplementary Section S2.5.

### 2.3 Using the shiver code

shiver and its documentation are available at https://github.com/ChrisHIV/shiver. It was designed to be run in Linux-like environments, including Mac OS. Once dependent packages are installed, shiver itself requires no installation: it is a set of executable scripts. The Genomic Virtual Laboratory (Afgan et al. 2015), provided for example on the UK Medical Research Centre’s Cloud Infrastructure for Microbial Bioinformatics (MRC CLIMB) (Connor et al. 2016), contains all dependencies (except smalt, which is loaded on MRC CLIMB with the single command brew install smalt, and otherwise available at http://www.sanger.ac.uk/science/tools/smalt-0), allowing shiver to be run immediately. The GitHub repository also contains a platform-independent virtual machine containing shiver with all of its dependencies pre-installed.

Before processing with shiver, short reads must be assembled into contigs. This important step, though difficult technically, is not onerous for the user: our chosen assembler IVA assembles contigs from reads with a single command from the command line, and can be run on a virtual machine provided by the Sanger pathogens group (http://sanger-pathogens.github.io/pathogens-vm/). The user can use any assembler; others are available in the Genomic Virtual Laboratory, including SPAdes, Velvet and MIRA, though currently none designed specifically for viral data.

shiver is run from the command line using three commands. Firstly, a one-off initialisation command:

 shiver_init.sh MyInitDir config.sh MyReferences.fasta \ MyAdapters.fasta MyPrimers.fasta

(the slash indicating that one command is here being split over multiple lines), which sets up an initialisation directory of files for shiver based on the user’s choice of existing references, and adapter and primer sequences to remove. Subsequently, for each sample to be processed, one command blasts, corrects and aligns the contigs:

 shiver_align_contigs.sh MyInitDir config.sh \ MyContigs.fasta MyID

where MyID is used for labelling output. After inspection of the corrected contigs aligned to the existing references, a second command constructs a tailored reference for mapping, preprocesses the reads, maps them and calls the consensus:

 shiver_map_reads.sh MyInitDir config.sh \ MyContigs.fasta MyID MyID.blast \ MyAlignedContigs.fasta MyForwardReads.fastq \ MyReverseReads.fastq

This produces, for each sample,

- the mapped reads in BAM format;
- a plain text file with the counts of the different bases at each position, also including HXB2 coordinates (by default; not relevant for non-HIV samples);
- the consensus;
- a coordinate-translated version of the consensus for a global alignment; and
- the insert-size distribution.

The global alignment of consensuses produced from all samples is constructed simply by combining the coordinate-translated consensus files from all samples into one file, e.g. running from the command line

 cat file1 file2 […] > MyGlobalAlignment.fasta

For our data, shiver typically look less than an hour to process each Miseq sample, and up to ten hours for each Hiseq sample (the latter containing roughly ten times as many reads), on a single core of the Imperial College London High-Performance Cluster (which is a mixture of computational resources with different specifications).

All bioinformatic parameters can be changed in the configuration file (config.sh above), allowing customisation of how reads are trimmed, how they are mapped, and how the consensus is called as a function of coverage and diversity. shiver also includes simple command-line tools for partial reprocessing (modifying sample output without rerunning the whole pipeline), and for analysis—see Supplementary Section S3.

### 2.4 Example data and its processing by shiver

We used two datasets as examples for processing with shiver. The first was sixty-eight publicly available Miseq samples: those sequenced and released with the IVA publication (Hunt et al. 2015), namely accession numbers ERR732065–ERR732132 on the European Nucleotide Archive. The samples have different origins; six are from a longitudinally sampled transmission pair studied by Brener et al. 2015. ERR732065–ERR732072 were sequenced with 150 bp reads, ERR732073–ERR732132 with 250 bp reads. Only forty-two of these sixty-eight samples were assembled by Hunt et al. 2015: the rest failed quality control checks designed to pre-select robust whole-genome samples. After downloading the short reads from the European Nucleotide Archive, we reassembled all sixty-eight samples with IVA for processing with shiver, as by design our method can be run in exactly the same way for those samples devoid of genuine sequence, those with partial genomes and those with whole genomes.

The second dataset was fifty Hiseq samples newly generated for the BEEHIVE project, from confirmed seroconverters from Europe. RNA was extracted manually from blood samples following the procedure of Cornelissen et al. 2016. This was amplified using universal primers that define four overlapping amplicons spanning the whole genome, following the procedure of Gall et al. 2012. Specifically, 5 μl of Amplicon 1 (the shortest and most successfully amplified amplicon) was pooled with 10 μl each of Amplicons 2–4. Multiple samples were pooled during library preparation, using one of 192 multiplex adaptors for each sample. The library was sequenced in ‘rapid run mode’ on both lanes of a HiSeq2500 instrument with read lengths of 2 × 250 bp, resulting in two lanes of short reads per sample. Automatic processing at the Wellcome Trust Sanger Institute used IVA to generate contigs for each lane, i.e. two sets of contigs per sample. We combined the two sets to allow comparison of the assembly output resulting from two technical replicates of short reads. For the large majority of cases the contigs were nearly identical, but stochastic differences in the read populations between lanes mean the resulting contigs occasionally differ.

The fifty Hiseq samples were chosen from a larger dataset currently being collected and sequenced for the BEEHIVE project’s primary aim of investigating the viral-molecular basis of virulence. Selection criteria for inclusion in the project include a known date of infection, either by negative and positive tests separated by less than a year, or by clinical signs of acute infection at diagnosis; and a sample obtained for sequencing between 6 and 24 months after diagnosis, before beginning antiretroviral treatment and before progression to AIDS. The fifty samples processed here were chosen as follows. (1) One sample chosen with a large difference in the fraction of the genome assembled between the two Hiseq lanes, as an example of the variability of assembly output. (2) Nine samples chosen with misassembled contigs for one or both Hiseq lanes, to illustrate the necessity of shiver’s contig correction. (3) From each of the Dutch, French, German and Swiss cohorts, ten samples with contigs spanning the whole genome: five subtype B and five non-B samples (subtype was determined with the COMET software (Struck et al. 2014)).

The existing reference set we used was the 2016 ‘compendium’ group M genome alignment from the Los Alamos National Laboratory, with a small amount of sequence trimmed from both edges of the alignment to match the region of the genome amplified by the sequencing protocol used for all data here (Gall et al. 2012), which partially excludes the flanking long terminal repeat regions.

For comparison with shiver’s constructed mapping reference, for each sample we used kallisto (Bray et al. 2016) to pseudo-align all the reads, using an index constructed from 3,249 whole genome references from the Los Alamos National Laboratory HIV database (those in the 2016 ‘all genome’ alignment) together with the whole human genome (as an attractor for human contaminant reads). We defined the closest existing/real reference sequence for that sample as the one with the highest transcript per million score.

For this analysis, we set the minimum coverage threshold (the number of mapped reads required to call the base at each position) to be 10 throughout, since the assembler we used—IVA—requires at least ten reads to extend a contig, and we compare the consensus to the contigs.

To illustrate application of shiver outside of HIV, we used it to process Illumina paired reads from a whole-genome Hepatitis C Virus (HCV) sample: accession number DRR000928 on the European Nucleotide Archive. We assembled the reads into contigs using SPAdes (Bankevich et al. 2012), and for the existing reference set required as shiver input we used the 2008 ‘all genome’ alignment of 471 references from the Los Alamos National Laboratory HCV database (Kuiken et al. 2005). We also ran shiver on Illumina paired reads from a whole-genome Respiratory Syncytial Virus (RSV) sample: accession number ERR438932 on the European Nucleotide Archive. We assembled the reads into contigs using SPAdes, and for the existing reference set we used the sixty-three whole genomes sequenced by Bose et al. 2015 from four continents to help capture global RSV diversity. For both the RSV and HCV reads we used kallisto to identify the closest sequence in the existing reference set, in the same manner as described above for the HIV dataset.

## 3. Results

We ran shiver on the paired-end short read HIV data described earlier—sixty-eight Illumina Miseq samples and fifty Illumina Hiseq samples. Only sixty-five of the Miseq samples had at least one contig that returned a BLASTN hit to a sequence in our chosen set of existing references; these and all fifty Hiseq samples were fully processed, giving whole or partial genomes. We produced consensus sequences, together with summary minority-variant information (base frequencies at each position) and detailed minority-variant information (all reads aligned to their correct position in the genome).

For comparison, for each sample we also mapped to the closest existing reference sequence identified using kallisto, instead of the shiver reference. We used the same mapping parameters, mapped the same set of reads (following shiver’s removal of adapters, primers, low-quality bases and contaminant read pairs), and called the consensus of the mapped reads in the same way (still using shiver), i.e. we changed only the reference sequence used for mapping.

Supplementary Sections S4 and S5 contain figures showing, for each sample, the genes of HIV in their reading frames, a set of sequences connected to this sample, and the coverage (number of reads mapped at each position) along the genome. We reproduce the figure for the first Miseq sample here (Fig. 5)—as an example for discussion. We see that there is no sequence data in the region around the *vif* and *vpr* genes, which is the part of Amplicon 3 in this sequencing protocol that is not overlapped by neighbouring Amplicons 2 or 4. Evidently Amplicon 3 failed to amplify for this sample. There is no contig sequence in this region, a coverage less than the threshold of 10, and so consensus sequence was not called. (The information contained in the few reads that did map to this region is retained in the minority-variant files produced by shiver; consensus sequence could be called here, if one chose to lower the minimum coverage threshold parameter below 10.)

Comparisons of these sequences are quantified for each sample in Supplementary Table S1, and in summary in Tables 1 and 2. For example Table 1 shows that mapping a sample’s reads to the shiver reference instead of the real reference, the median number of bases called differently and supported by higher coverage is 13; the median number of bases called differently but with equal or lower coverage is 0. Interpreting higher coverage as more accurate mapping, mapping to the shiver reference instead of the real reference typically corrects thirteen false SNPs per sample. For this comparison we only considered positions where a base was called in both consensuses, but the base differed. As in the case of Fig. 2, inaccurate mapping may also result in a stretch of sequence being missed from the consensus. The median increase in the consensus sequence length when mapping a sample’s reads to the shiver reference instead of the real reference is 205 bp.

**Table 1.** Comparing the consensus from mapping to the reference constructed by shiver with the consensus from mapping to the closest identified real reference.

Number of bases called differently, with higher coverage when mapping to the shiver reference than to the real reference	Min	0
	Median	13
	Mean	16.8
	Max	57
Number of bases called differently, with higher (or equal) coverage mapping to the real reference than to the shiver reference	Min	0
	Median	0
	Mean	1.2
	Max	24
Extra length of the shiver consensuses compared with the real reference’s consensus (in number of bases)	Min	−54
	Median	205
	Mean	239.4
	Max	1,262

Minima, medians, means, and maxima are over the combined set of sixty-five Miseq and fifty Hiseq samples processed. Means are rounded to one decimal place.

**Table 2.** Comparing the consensus from mapping to the reference constructed by shiver with the contigs (after correction of the contigs by shiver).

Length of sequence present in the contigs but missing from the consensus	Min	0
	Median	0
	Mean	0
	Max	0
Length of sequence present in the consensus but missing from the contigs	Min	0
	Median	0
	Mean	114.1
	Max	2,443
Number of positions where all corrected contigs disagree with the consensus	Min	0
	Median	7
	Mean	13.7
	Max	106

Minima, medians, means, and maxima are over the combined set of sixty-five Miseq and fifty Hiseq samples processed. Means are rounded to one decimal place.

Table 2 shows that, for more than half of the samples, the shiver consensus is no longer than the set of contigs (the median length increase is zero). However it is occasionally much longer—see the relevant column of Supplementary Table S1—due to assembly failure. The median number of bases in the shiver consensus that differ from all contigs at that point is 7. (Where the contigs disagree amongst themselves but one agrees with the consensus, we count this as agreement.) As the consensus is derived by mapping to the contig sequence at such points and calling the most common base, such positions of disagreement are probably improvements. Seven corrected SNPs is a highly conservative estimate of the improvement over the contigs, however, as the comparison was made after shiver performed contig correction (including both structural correction and trimming of contig ends where they have no BLASTN hit). This is because a base-by-base comparison of two sequences requires them to be aligned, and aligning the *spliced* or partially reverse-complemented contigs that shiver corrects (see Section 2.1) would give a nonsensical alignment. In addition, deriving the consensus from mapping instead of relying solely on *de novo* assembly means that minority-variant information is available.

As mentioned in Section 2, nine of the Hiseq samples were chosen as illustrations of misassembled contigs, and twenty-three of the Miseq samples with HIV contigs (twenty-six including those without HIV contigs) were not considered in the IVA publication due to failing sample quality control checks. These samples are identified in Supplementary Table S1. The statistics for shiver’s performance for these nine Hiseq samples are not worse than those for the all the data, e.g. a median of thirty-one bases called differently with higher coverage in the shiver consensus, and 0 bases called differently with higher coverage mapping to the real reference. This illustrates that problematic contigs do not mean that mapping to an existing reference becomes preferable, thanks to shiver’s contig correction. The IVA QC failures are mostly partial genomes; statistics for these samples are scaled down from their values for the whole data set due to these being shorter sequences. An exception is the increase in the consensus sequence length over the length of the contigs, whose median value is 0 for the whole dataset but thirty-two for the QC failures. It is not surprising that contigs should be shorter than mapping-derived consensuses for problematic samples previously excluded from consideration for assembly.

These improvements from using shiver are small compared with the length of the HIV genome—roughly 9,000 bases. However the aim of sequencing a known pathogen is not to produce a roughly correct picture of the known genome, but to obtain each sample’s sequence as accurately as possible, so that small numbers of differences between similar samples can be meaningfully interpreted.

**Figure 6.**
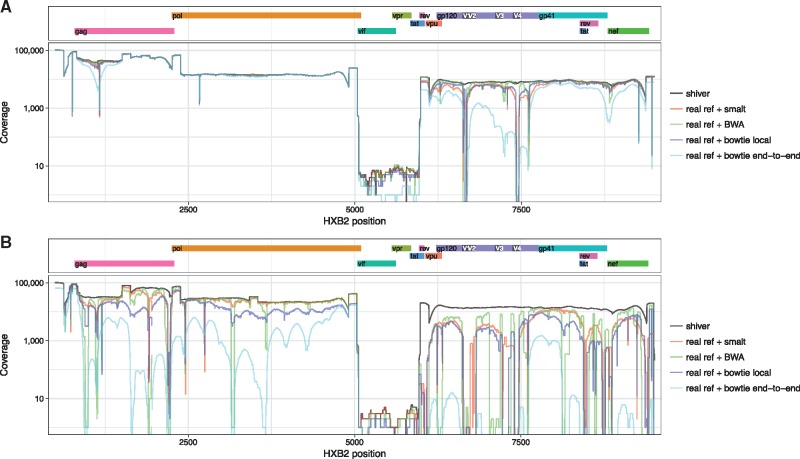
Coverage over the genome following different mapping strategies. The line marked ‘shiver’ is for mapping to the reference constructed for the sample using contigs, with smalt. The other lines are for mapping to the closest identified real reference using the indicated mapping algorithm. (A) is the same sample shown in Fig. 5; (B) is Miseq sample ERR732071, a more extreme example of mapping failure using the closest real reference.

The problems arising from mapping to a reference that differs from the sample in question do not arise simply from an inappropriate choice of mapper. To illustrate this, for the Miseq dataset we also mapped the reads to their closest real reference sequence using BWA and bowtie in both its ‘local’ and ‘end-to-end’ modes (for both mappers we used their default settings except for specifying a maximum insert size of 2,000 for bowtie, retaining only properly paired reads as we did with smalt). Figure 6A shows the resulting coverage along the genome for the same sample shown in Fig. 5. Localised drops in coverage indicate the same problems described previously. This was common across all of the samples; Fig. 6B shows a more extreme example, for which mapping to the closest real reference using any of the mappers performs very poorly.

**Figure 7.**
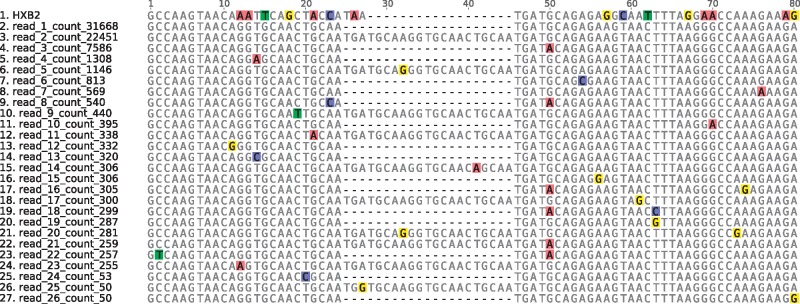
Within-host indel polymorphism in our Hiseq sample 17796_3_29: a 21 bp insertion in roughly a third of the reads duplicates the GATAMMQ amino acid motif at the boundary between p2 and p7 in *gag*. The value following ‘_count ’ in the sequence name is the number of times that exact sequence was found in the reads here following mapping with shiver; only sequences found at least fifty times are shown. HXB2 is included for comparison. Coloured squares highlight bases differing from the consensus; bases without a coloured square agree with the consensus base at that position (ignoring gaps).

Among the reads mapped by shiver, interesting within-host diversity is maintained, capable of revealing structure in the quasispecies. Figure 7 shows an example for our Hiseq sample 17796_3_29. The reads are from the boundary between p2 and p7 in the *gag* gene; roughly a third of them have a 21-bp insertion relative to the others. This insertion is not seen in any other sequence in the Los Alamos National Laboratory alignment ‘HIV1_ALL_2015_gag_DNA’ of 7,903 *gag* sequences (http://www.hiv.lanl.gov/). Though not a duplication at the nucleotide level, it duplicates the GATAMMQ amino acid motif. Mutations at the p2/p7 boundary (Ho et al. 2008) and insertions at other *gag* cleavage sites (Tamiya et al. 2004) have been implicated in restoring replicative capacity in viruses treated with protease inhibitors.

For the HCV sample, compared with mapping to the closest existing reference identified from the reads, shiver called nine bases differently, all supported by higher coverage. shiver also recovered a 15-bp stretch of sequence that was missing from the consensus after mapping to the closest existing reference. These nine different base calls and 15 bp of sequence were close together at the start of the E2 gene. There were no indels between the sample and the closest existing reference here, but a very high density of SNPs which prevented accurate mapping of the sample’s reads to the closest existing reference.

For the RSV sample, compared with mapping to the closest existing reference identified from the reads, shiver called only one base differently, supported by higher coverage. Clearly, examining only a single sample does not allow us to draw any conclusions; however, this much more modest improvement in using a constructed reference over an existing reference for RSV is not surprising. The smaller amount of diversity in RSV (especially within each of its two distinct subgroups, A and B) compared with HIV or HCV should make it easier to find an existing reference with a very high degree of similarity to the sample in consideration. On the other hand, for viruses exhibiting less diversity, each erroneous base call will have greater impact on comparative analyses; shiver may therefore still be useful in these cases.

In this methods article we do not present conclusions drawn from analyses of sequences generated with shiver. Two such analyses published so far are by Blanquart et al. 2017 and Ratmann et al. 2017. Blanquart et al. 2017 determined that the fraction of variability in HIV set-point viral load that is explained by viral genetic factors was around one third, using 1,373 European whole genomes. Ratmann et al. 2017 found predictors of HIV sequencing success or failure for 3,985 African whole genomes, and studied the effect of the observed amplification failure patterns on phylogenetic inference. Analysis of both genomic datasets is ongoing.

## 4. Discussion

We developed the tool shiver to preprocess and map reads from each sample to a custom reference, constructed using *de novo* assembled contigs supplemented by existing reference genomes. Tailoring the reference to be as close as possible to the expected consensus before mapping maximises the accuracy of the mapping, and therefore of the resulting consensus. shiver’s identification, ranking, and use of the closest existing references to fill in gaps between contigs boosts data recovery for samples with amplification failure or assembly failure. Such partial-genome samples, which are inevitable in large diverse data sets, are processed with exactly the same two commands; this simplifies scripted application of shiver to all samples in a data set. shiver also produces a global alignment containing all of the consensuses separately generated for each sample, which is usually required for comparative analysis of the sequences such as for phylogenetics or GWASs.

Mapping to shiver’s constructed reference instead of mapping the same reads to the closest identified real reference gives a median increase in consensus sequence length of 205 bp, with thirteen of the original bases called differently and more accurately. This shows the importance of tailoring the reference to the sample before mapping. shiver’s consensus, obtained by mapping reads to a reference constructed from the contigs, has a median of 7 bases called differently from the contigs even after correcting structural problems in the contigs and trimming suspicious sequence from their ends. This illustrates the need for mapping in addition to assembly.

A limitation of the method is that after reads have been successfully mapped (which imposes requirements on base quality and good alignment to the reference), we consider each read to carry equal weight in determining the consensus and the frequency of variant bases. The frequency of a variant in the reads and its frequency in the sampled virions may differ due to PCR bias—amplification of some virions more than others. A proper reconciliation of these frequencies would require modelling the number of virions in the sample, their diversity, the process generating PCR bias, and sequencing error, which is beyond the scope of this work. Included in shiver is the option to *deduplicate* mapped reads based on their position: from each set of paired reads with identical mapped coordinates, retaining only one pair and discarding the rest as suspected PCR duplicates (using Picard). This is turned off by default, as decreasing the coverage and discarding some diversity in the reads may not be appropriate for every sequencing protocol. We do not include an option for removal of duplicate reads before mapping based on exact sequence matches, as this preferentially retains reads with sequencing error. Instead of addressing the problem of PCR bias at the analysis stage, it can be addressed with the sequencing protocol: primer IDs (Jabara et al. 2011) can associate every read to its template, allowing identification of all PCR duplicates (as well as permitting separate reconstruction of all haplotypes). As with SGA however, higher costs for each sample currently limit applicability to large population studies.

Another limitation is that no mapping of diverse reads can guarantee perfect accuracy at every position in every sample, as perfect sequence alignment is an unsolved problem. In particular where samples contain indel polymorphisms, or where localised misassembly results in an indel not present in the reads, mapping may misalign reads in a way that is not cured by remapping to their own consensus, since the misalignment gives an error in the consensus. As with all automatic sequence alignment, there is scope for improvement by manual inspection. shiver’s performance is also linked to that of the assembler used to produce the input contigs. For a sample with parts of the genome where assembly failed to produce contigs, shiver’s reference is constructed using the closest identified reference in lieu of the missing contigs. For such samples the bias of mapping to an existing reference is still present to some degree, though mitigated by shiver’s option to map a second time to its initial consensus.

For sequences that are recombinants of a type not seen in existing reference sets, shiver will nevertheless construct an appropriate reference for mapping provided contigs were fully assembled from the available reads, i.e. either the contigs span the whole genome, or they are missing only where reads are missing. As shiver fills in gaps between contigs using the single closest existing reference (supplemented by further existing references only at the ends, i.e. if the closest reference is shorter than some others), in the event of partial assembly failure for a novel recombinant this might not produce a mapping reference as well tailored to the sample as some process of mixing different existing references at different parts of the genome to locally match the available contigs. However shiver’s second round of mapping to the first round’s consensus will partially mitigate this, and as novel recombinant samples with partial assembly failure are expected to be rare (noting that the success of *de novo* assembly is independent of subtype or recombination), we prefer not to mix existing references throughout the genome, for simplicity and robustness to reference misalignment.

A design choice is that shiver does not take into account translation to amino acids, and in particular does not bias towards maintaining reading frames. Deliberately including this bias would be clearly justified for many organisms, but the case is arguable for HIV due to overlapping reading frames, frame-shifting polymorphisms, and possibly antisense expression (Miller 1988; Cassan et al. 2016). Other tools exist to extract in-frame gene sequences from shiver consensuses, such as Gene Cutter (https://www.hiv.lanl.gov/content/sequence/GENE_CUTTER/cutter.html).

Individuals who are dually infected—hosting two distinct quasispecies, whether by two distinct founder viruses establishing productive infections, or by superinfection—are known to be special cases clinically, and perhaps for evolution, because of the opportunity for recombination. It is important to note that they are also special cases for bioinformatic processing (Giallonardo et al. 2014). If one of the two quasispecies is more highly represented in the reads at every position in the genome, the consensus sequence for the infected individual will be simply the consensus of the more abundant quasispecies. However if one quasispecies has more reads at part of the genome and the other has more reads elsewhere in the genome, the consensus will be a recombinant of both quasispecies; a recombinant which may never have existed *in vivo*, and which may invalidate phylogenies in which it is included. Clearly, care must be taken in identifying such individuals as their dually infected status may not be known.

Our focus here has been reconstruction of the consensus sequence that summarises a quasispecies. The process of doing this from diverse reads—from different virions in the quasispecies—retains rich information on within-host diversity. Our separate tool phyloscanner (Wymant et al. 2017) allows easy extraction, processing, alignment and parallel phylogenetic analysis of the short reads from many genomic windows of many mapped read files, for example those produced by shiver. Examination of within-host and between-host diversity together, at every position along the genome, allows identification of dual infections, transmission, recombination and contamination. These more detailed pictures of quasispecies and the relationships between them, in addition to their summaries as consensus sequences, further motivate the valuable role NGS has to play in our understanding of HIV.

## Data availability

The Miseq short reads processed here are publicly available on the European Nucleotide Archive: accession numbers ERR732065–ERR732132. The newly generated Hiseq short reads processed here will be made available subject to a data access request, to ensure patient confidentiality is protected.

## Supplementary data

Supplementary data are available at *Virus Evolution* online.

**Conflict of interest:** A.J.G. participated in an advisory board meeting for ViiV Healthcare in July 2016. K.P. is a member of the Viiv Dolutegravir’ Advisory Board and Viiv Data and Insights: Standardisation in Measuring and Collecting Care Continuum Data Advisory Board. H.G. reports receipt of grants from the Swiss National Science Foundation, Swiss HIV Cohort Study, University of Zurich, Yvonne Jacob Foundation, and Gilead Sciences; fees for data and safety monitoring board membership from Merck; consulting/advisory board membership fees from Gilead Sciences; and travel reimbursement from Gilead, Bristol-Myers Squibb, and Janssen. P.R. through his institution has received independent scientific grant support from Gilead Sciences, Janssen Pharmaceuticals Inc, Merck & Co, Bristol-Myers Squibb, and ViiV Healthcare; he has served on scientific advisory boards for Gilead Sciences and ViiV Healthcare and on a data safety monitoring committee for Janssen Pharmaceuticals Inc, for which his institution has received remuneration.

## Supplementary Material

Supplementary Data
