# Supplementary material for: Easy and accurate reconstruction of whole HIV genomes from short-read sequence data with shiver
Source: Virus Evol. 2018 May 18;4(1):vey007. doi: 10.1093/ve/vey007 (PMC5961307; doi:10.1093/ve/vey007)
Supplement: Supplementary Data [file vey007_supp.zip › PipelinePaper_v4_SI_AlnPlotsOnly.pdf]

Supplementary Sequence Alignment Figures for *Easy and Accurate Reconstruction of Whole HIV Genomes from Short-Read Sequence Data with SHIVER*

SI 4 Sequences and Coverage by Sample: Miseq Data

For each sample we show an alignment of the closest identified real reference, the reference constructed and used for mapping by `shiver`, the consensus of reads mapped to this reference, the consensus of reads mapped to the real reference (the exact same reads, i.e. following `shiver` preprocessing, mapped with all the same parameters), and the contigs. The contigs shown are those after any correction by `shiver`, since when misassembly gives partial reverse complements, their alignment gives a mess. The coverage (number of reads) resulting from mapping to each reference is shown with blue (`shiver` reference) and red (closest real reference) lines below the alignment. For both consensus sequences a minimum coverage of 10 was required to call the base at each position, since `IVA` requires a minimum of 10 overhanging reads to extend a contig. Thin black vertical lines inside sequences in the alignment denote SNPs (relative to the most common base amongst the sequences here). Thicker black horizontal lines indicate a lack of bases, i.e. a deletion relative to another sequence in the alignment or, for the two consensuses, simply missing sequence due to coverage being less than 10. Above each alignment are the genes of HIV in their respective reading frames.

Coverage is, in the first instance, defined with respect to the reference used for mapping. To plot the coverage from mapping to different references on the same axis we translate coordinates with respect to each reference to the coordinates of the alignment that contains both references. Where mapping reference A has a deletion with respect to mapping reference B, inside A's deletion (B's insertion) there are no coordinates with respect to A. We define the coverage for reference A at such points to be its last value before the deletion. This means that where one reference has an inappropriate indel that prevents some reads from being mapped, we will see its coverage drop while the other reference's coverage remains locally smooth.

HXB2 was initially included in each alignment for ease of determining the gene positions, and then removed. This is why in a few positions of a few alignments, all sequences present have a gap.

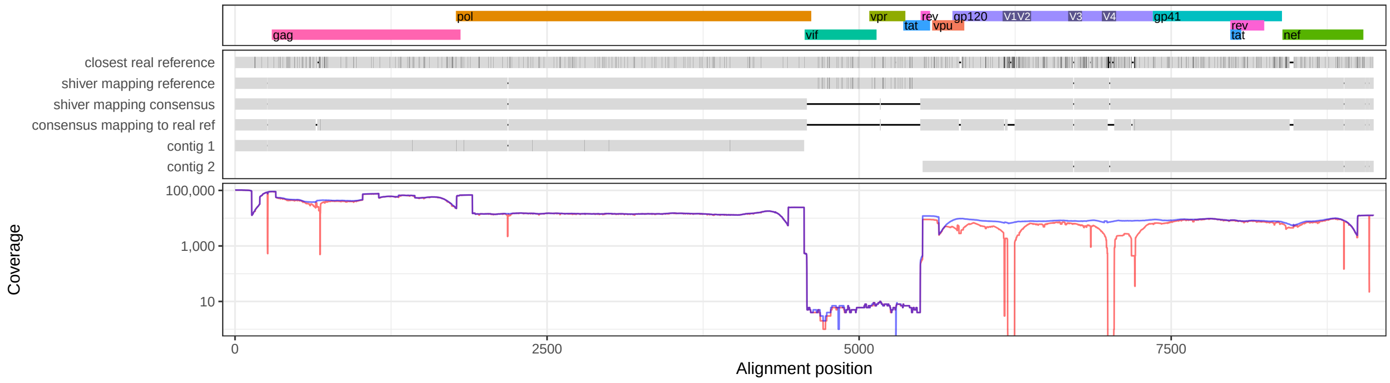

Figure S5: ERR732065 sequences and coverage (mapping to the `shiver` reference in blue, to the real reference in red).

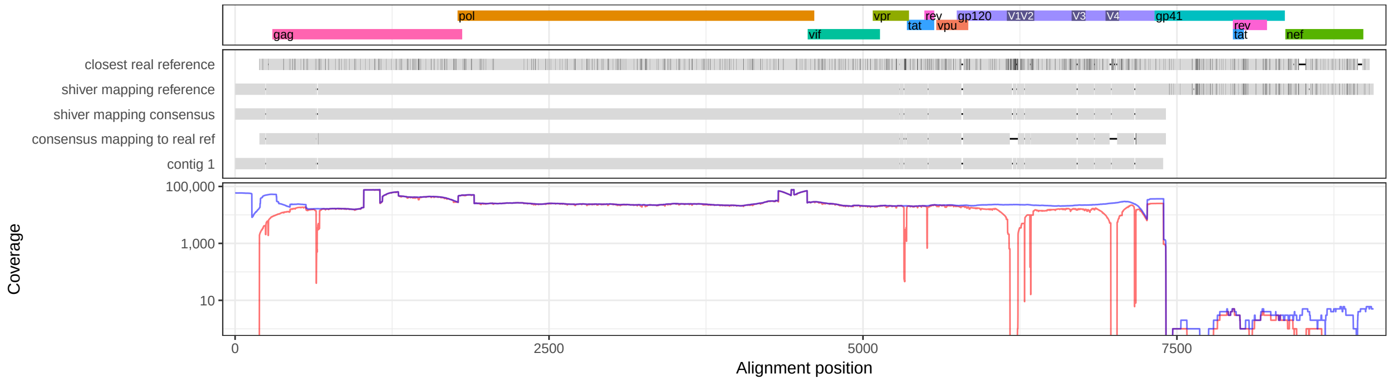

Figure S6: ERR732066 sequences and coverage (mapping to the `shiver` reference in blue, to the real reference in red).

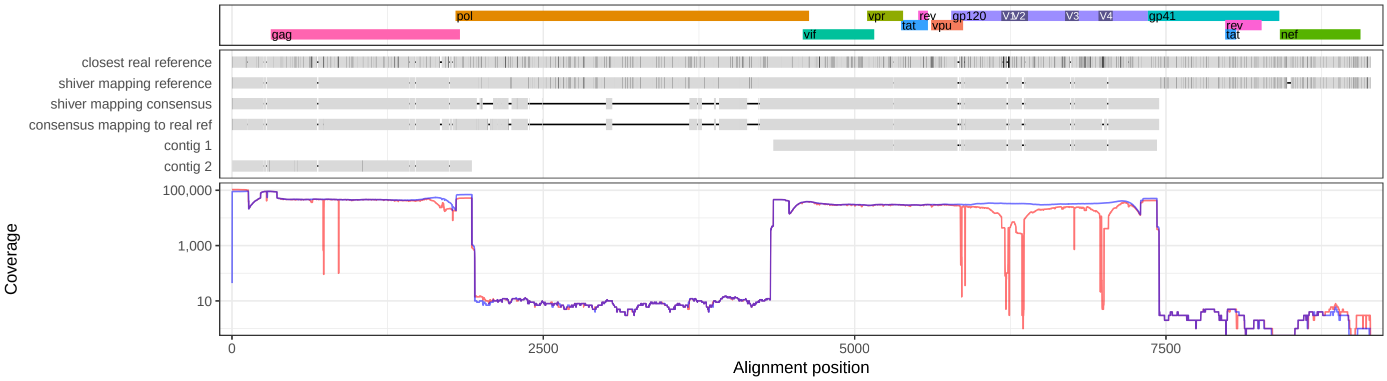

Figure S7: ERR732067 sequences and coverage (mapping to the `shiver` reference in blue, to the real reference in red).

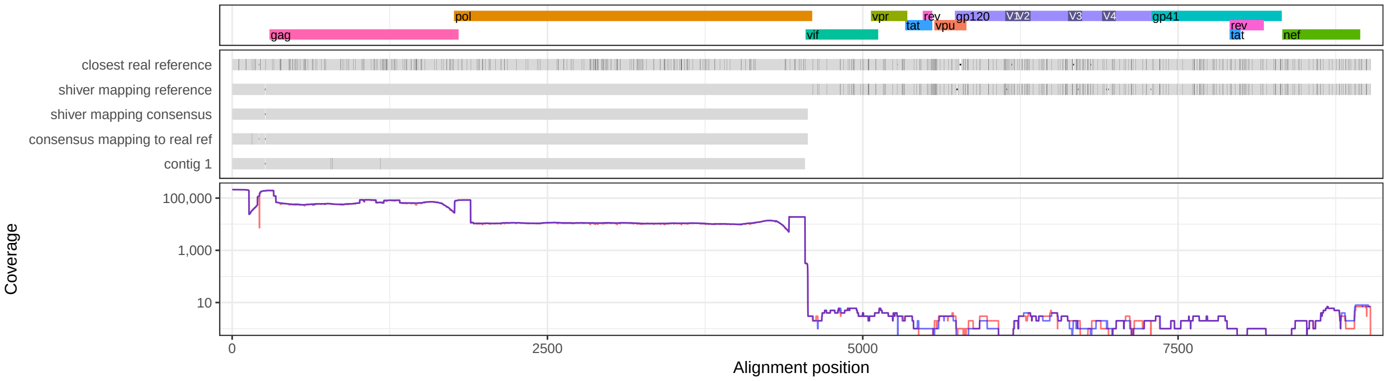

Figure S8: ERR732068 sequences and coverage (mapping to the `shiver` reference in blue, to the real reference in red).

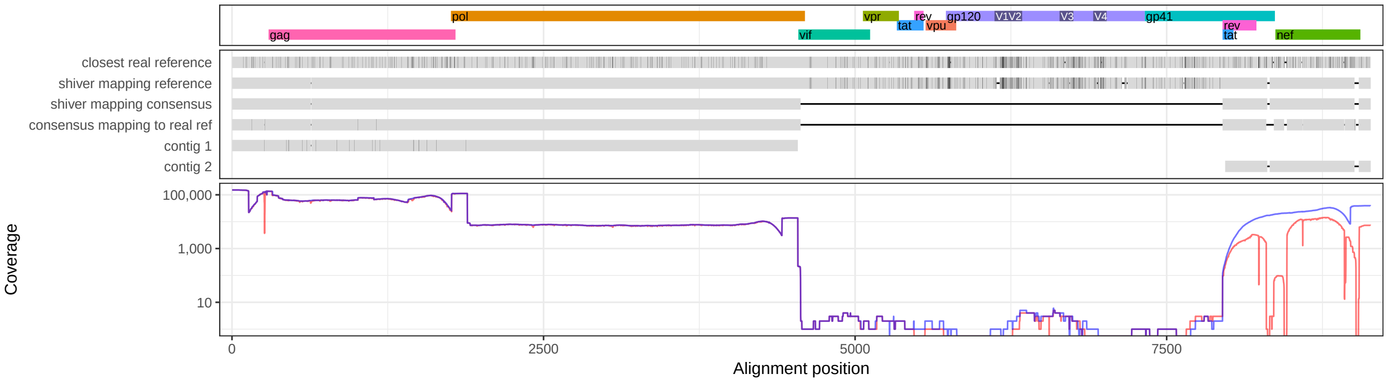

Figure S9: ERR732069 sequences and coverage (mapping to the `shiver` reference in blue, to the real reference in red).

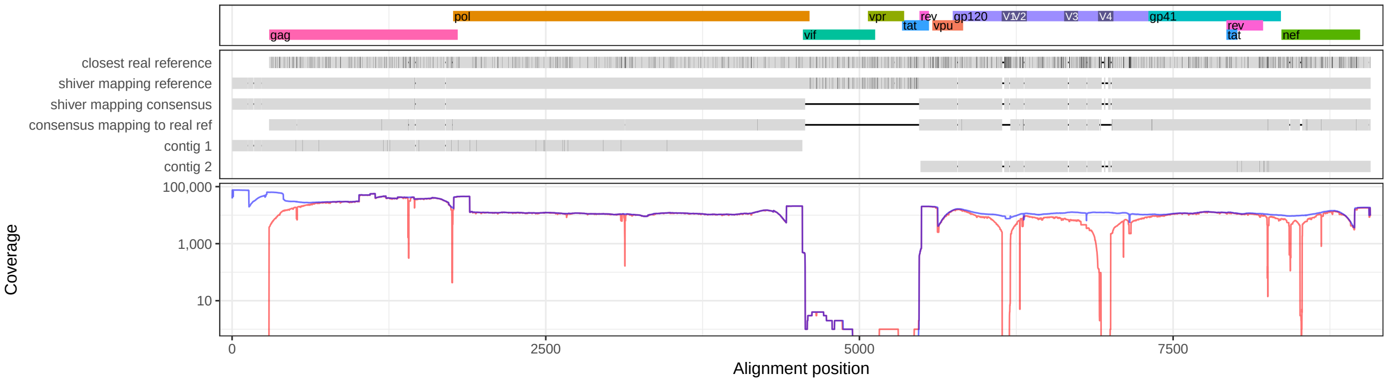

Figure S10: ERR732070 sequences and coverage (mapping to the `shiver` reference in blue, to the real reference in red).

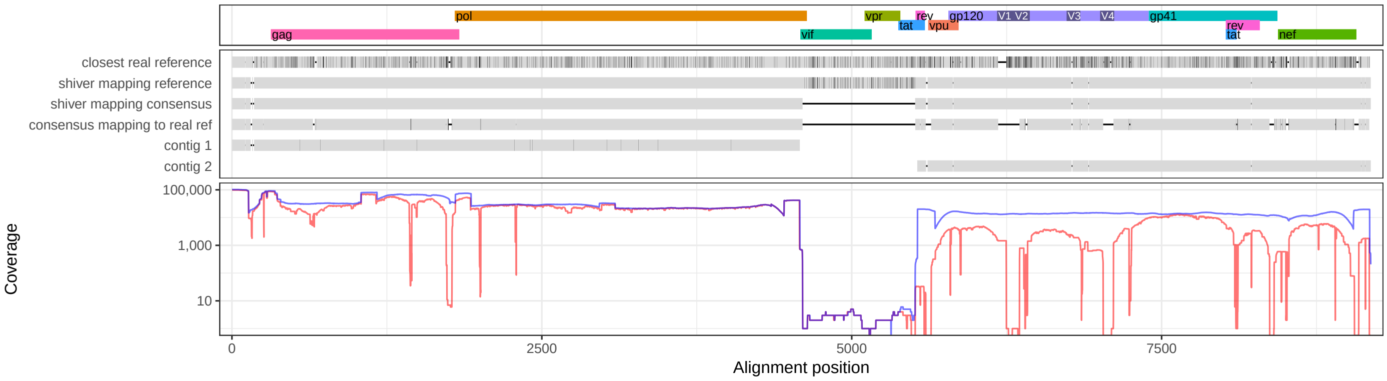

Figure S11: ERR732071 sequences and coverage (mapping to the `shiver` reference in blue, to the real reference in red).

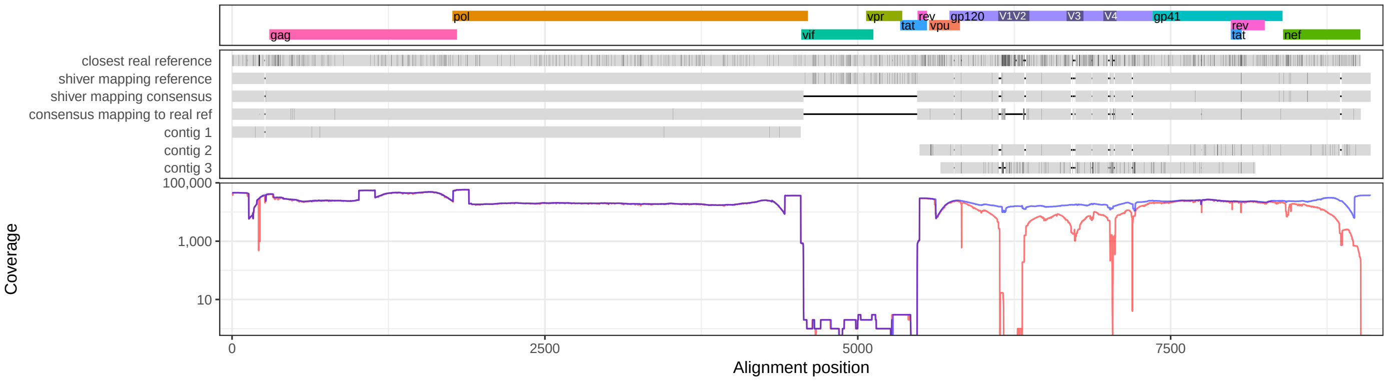

Figure S12: ERR732072 sequences and coverage (mapping to the `shiver` reference in blue, to the real reference in red).

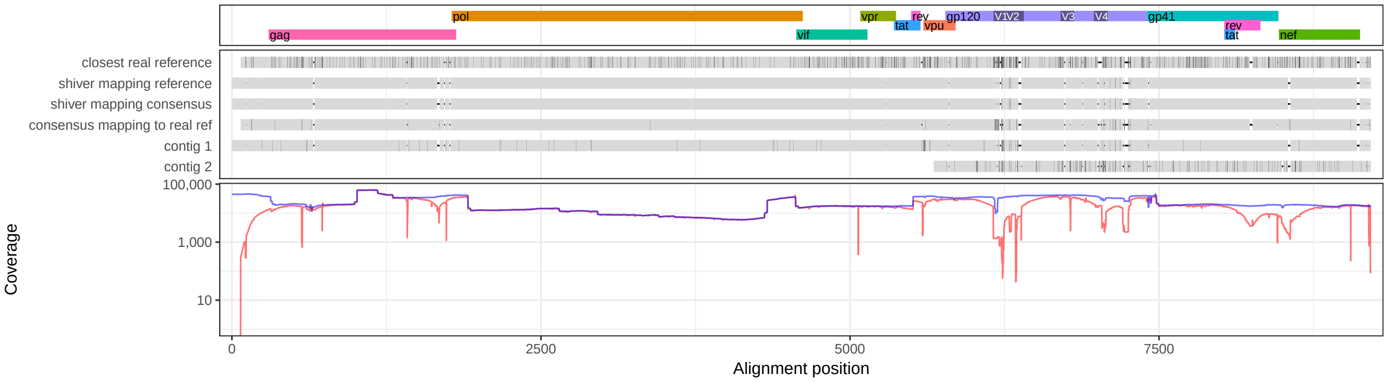

Figure S13: ERR732073 sequences and coverage (mapping to the `shiver` reference in blue, to the real reference in red).

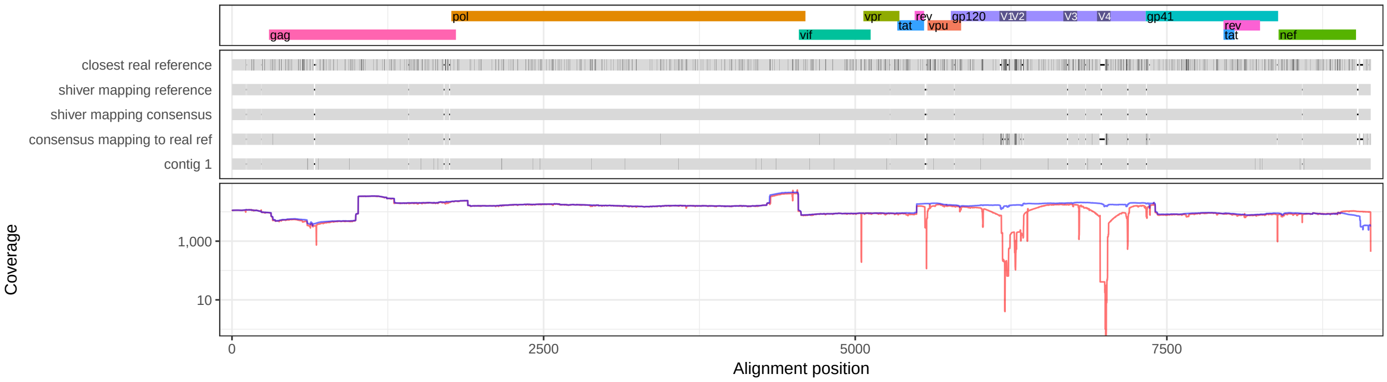

Figure S14: ERR732074 sequences and coverage (mapping to the `shiver` reference in blue, to the real reference in red).

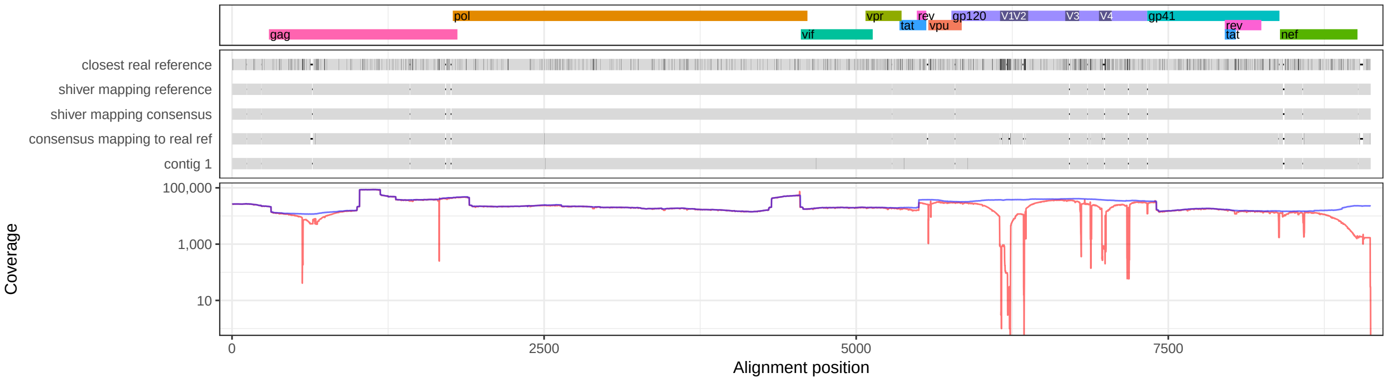

Figure S15: ERR732076 sequences and coverage (mapping to the `shiver` reference in blue, to the real reference in red).

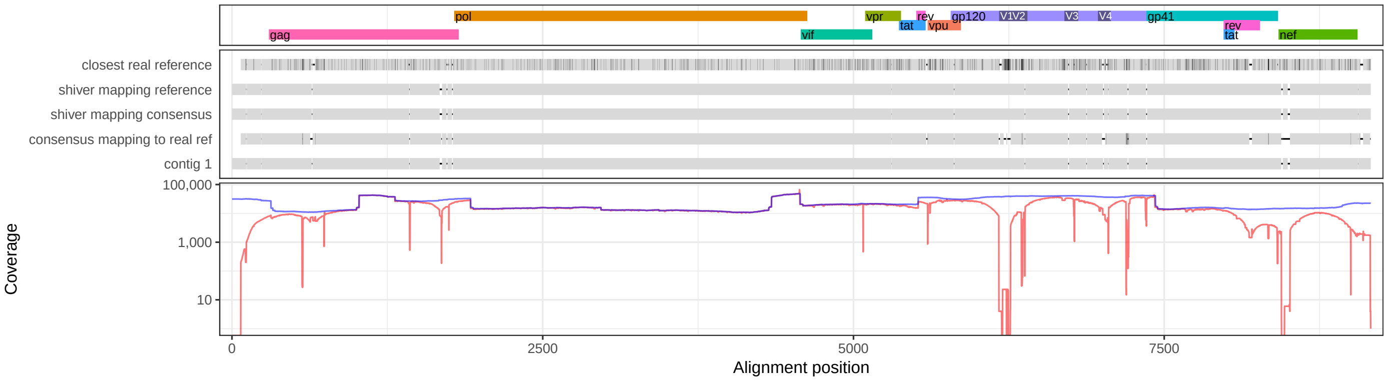

Figure S16: ERR732077 sequences and coverage (mapping to the `shiver` reference in blue, to the real reference in red).

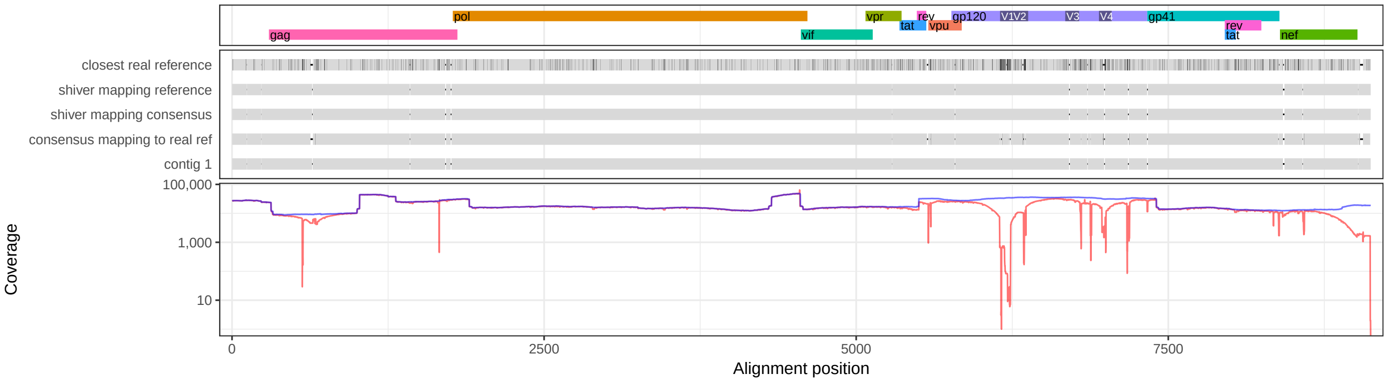

Figure S17: ERR732078 sequences and coverage (mapping to the `shiver` reference in blue, to the real reference in red).

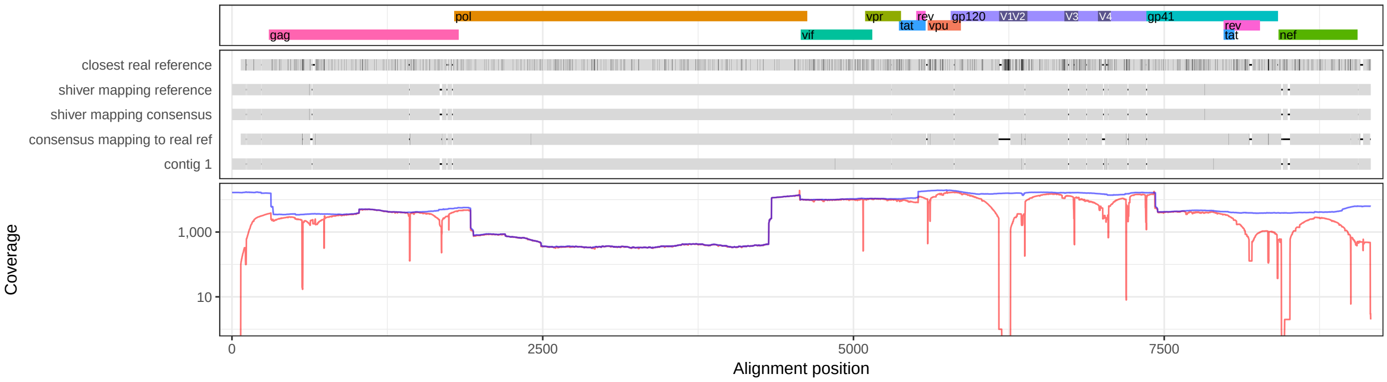

Figure S18: ERR732079 sequences and coverage (mapping to the `shiver` reference in blue, to the real reference in red).

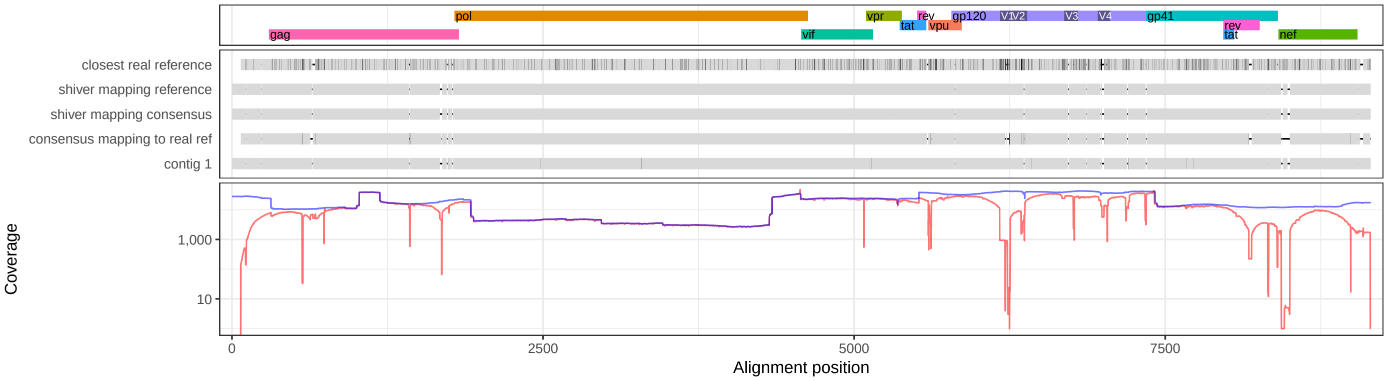

Figure S19: ERR732080 sequences and coverage (mapping to the `shiver` reference in blue, to the real reference in red).

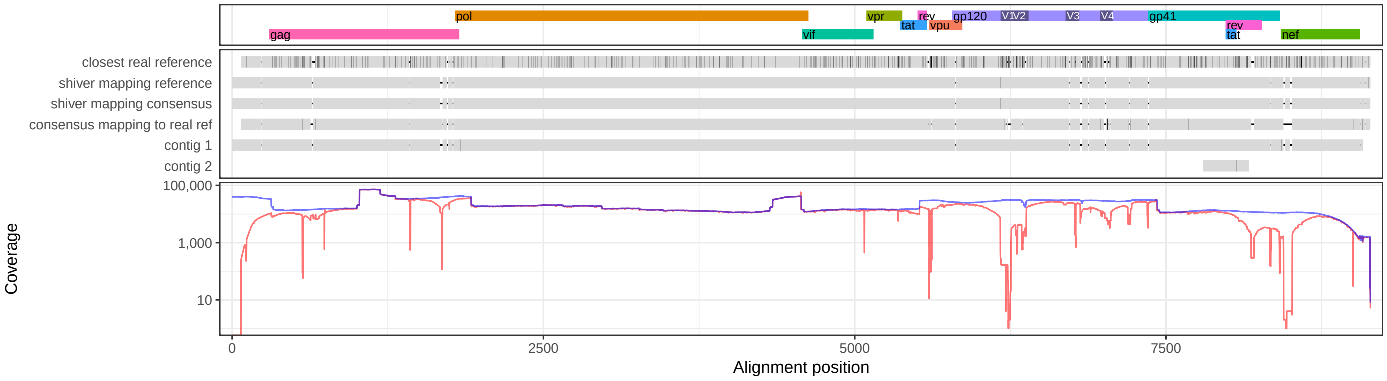

Figure S20: ERR732081 sequences and coverage (mapping to the `shiver` reference in blue, to the real reference in red).

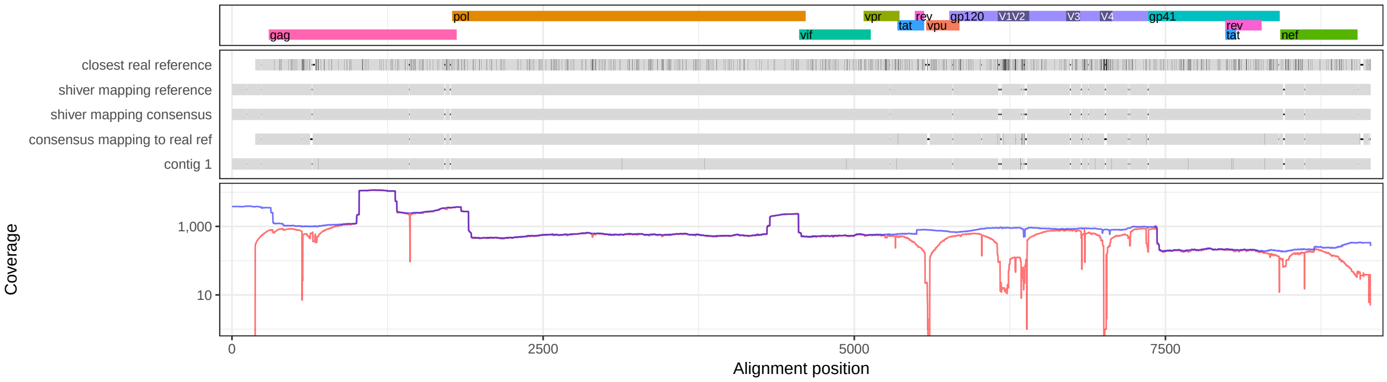

Figure S21: ERR732082 sequences and coverage (mapping to the `shiver` reference in blue, to the real reference in red).

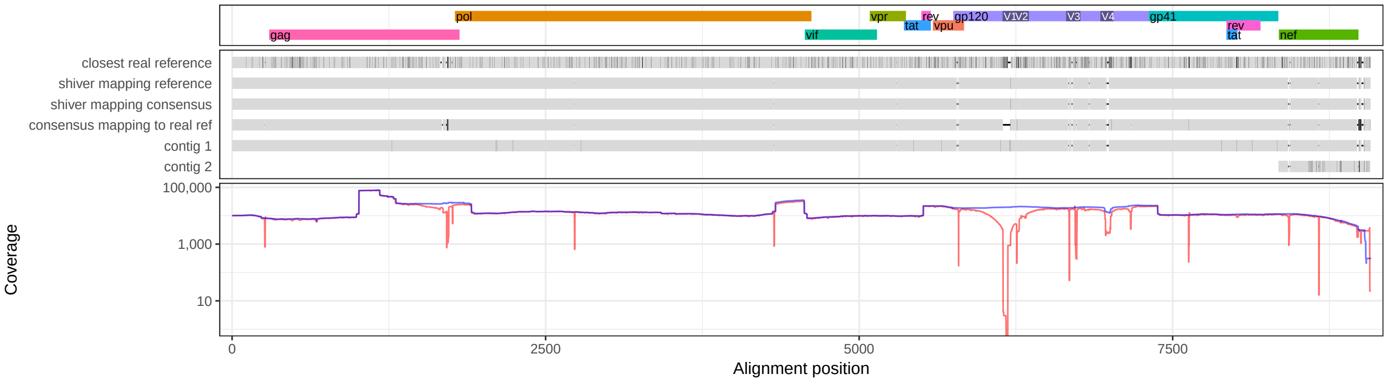

Figure S22: ERR732083 sequences and coverage (mapping to the `shiver` reference in blue, to the real reference in red).

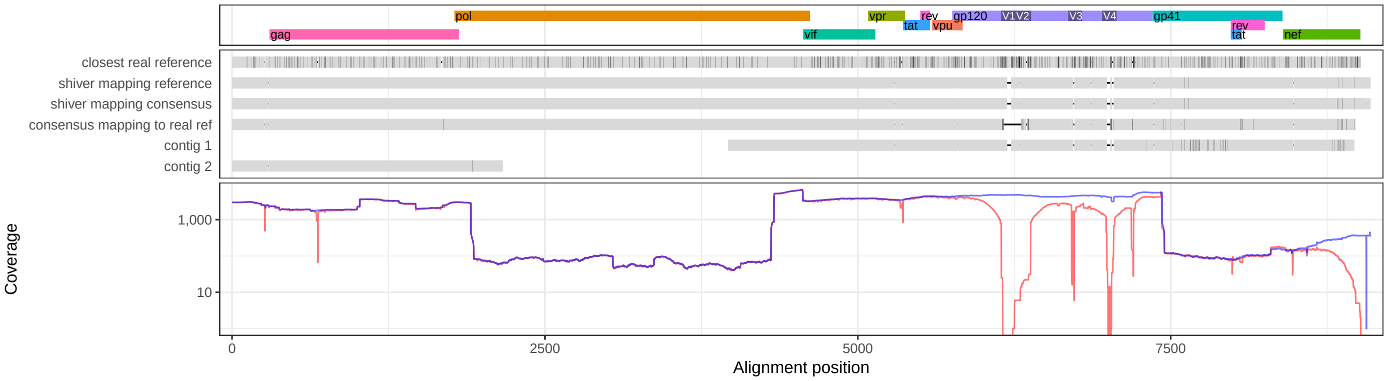

Figure S23: ERR732085 sequences and coverage (mapping to the `shiver` reference in blue, to the real reference in red).

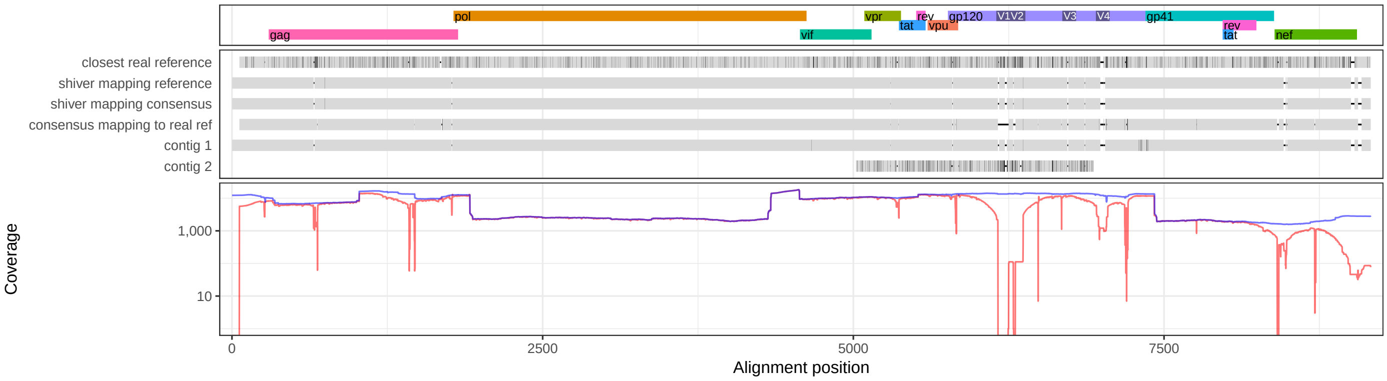

Figure S24: ERR732086 sequences and coverage (mapping to the `shiver` reference in blue, to the real reference in red).

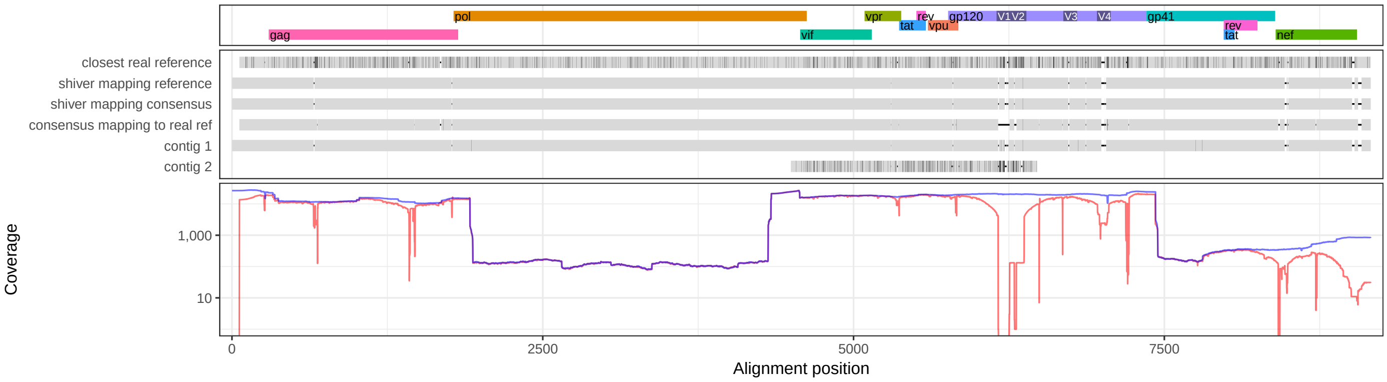

Figure S25: ERR732087 sequences and coverage (mapping to the `shiver` reference in blue, to the real reference in red).

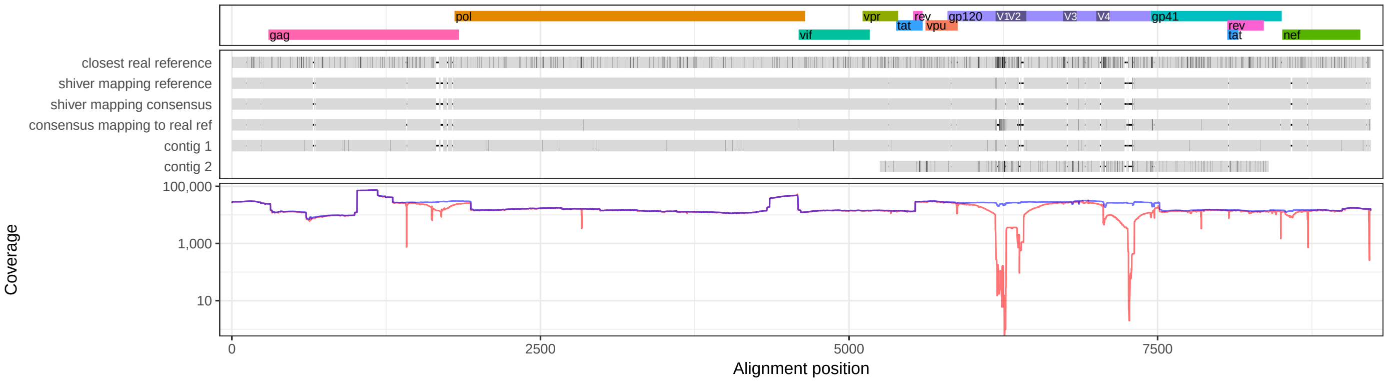

Figure S26: ERR732088 sequences and coverage (mapping to the `shiver` reference in blue, to the real reference in red).

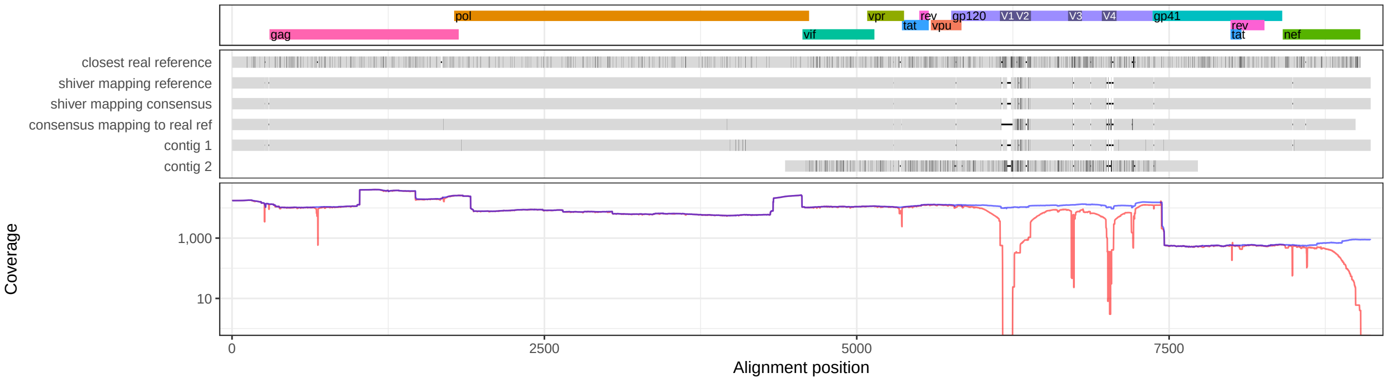

Figure S27: ERR732089 sequences and coverage (mapping to the `shiver` reference in blue, to the real reference in red).

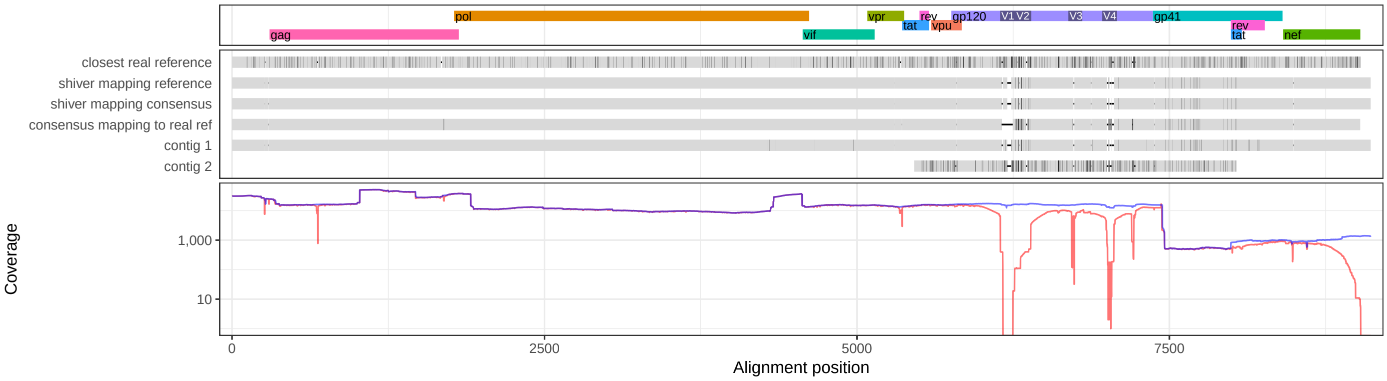

Figure S28: ERR732090 sequences and coverage (mapping to the `shiver` reference in blue, to the real reference in red).

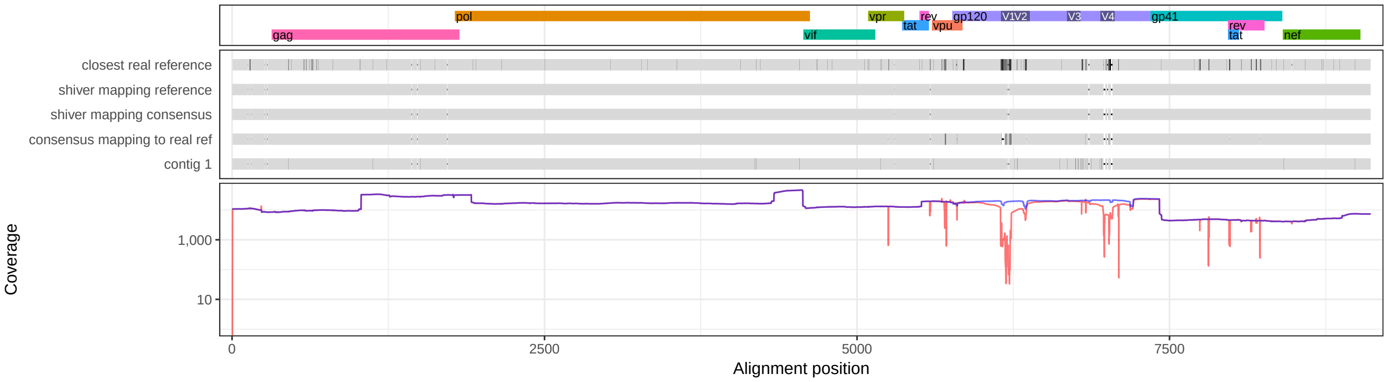

Figure S29: ERR732091 sequences and coverage (mapping to the `shiver` reference in blue, to the real reference in red).

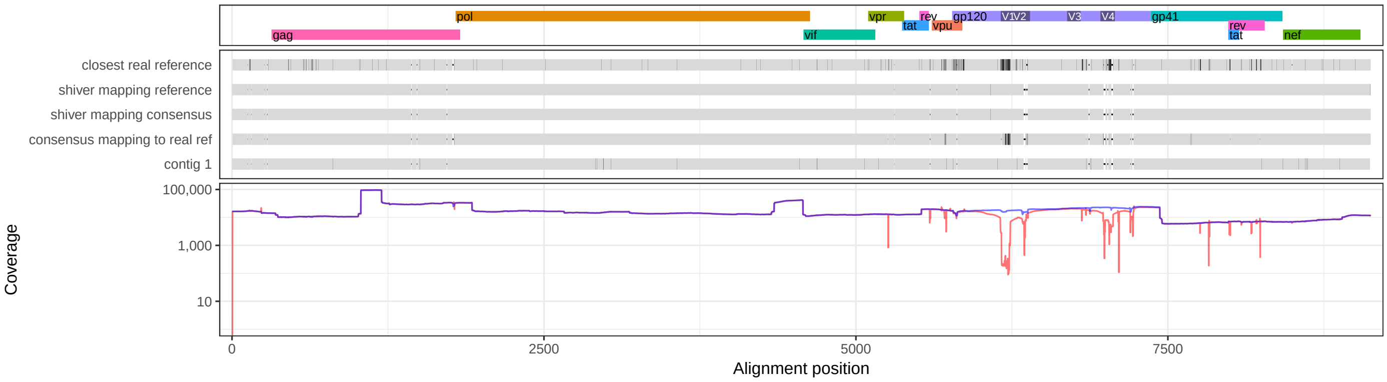

Figure S30: ERR732092 sequences and coverage (mapping to the `shiver` reference in blue, to the real reference in red).

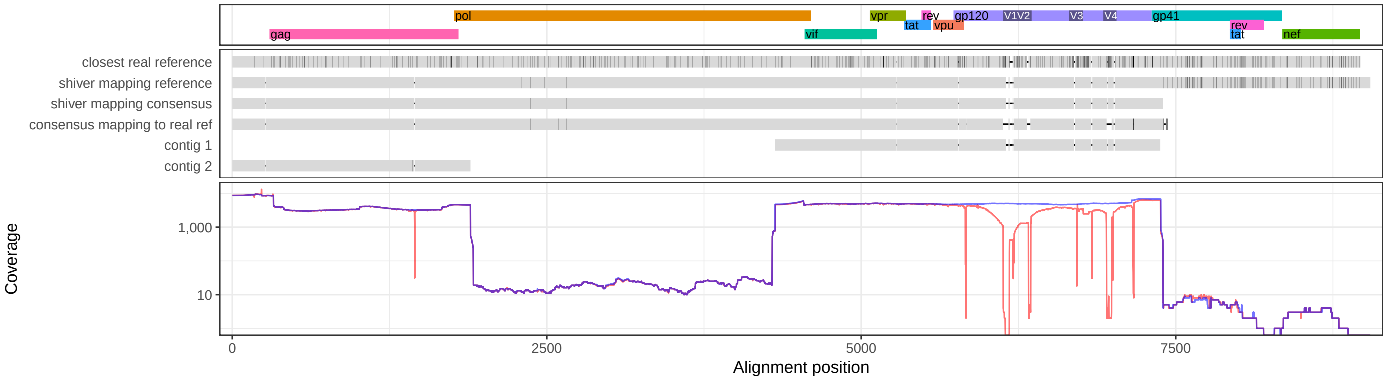

Figure S31: ERR732093 sequences and coverage (mapping to the `shiver` reference in blue, to the real reference in red).

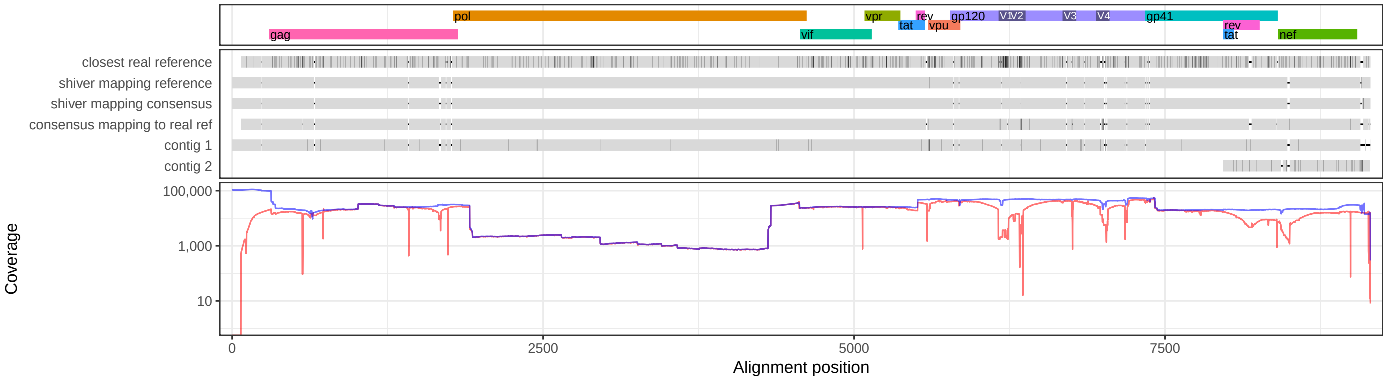

Figure S32: ERR732094 sequences and coverage (mapping to the `shiver` reference in blue, to the real reference in red).

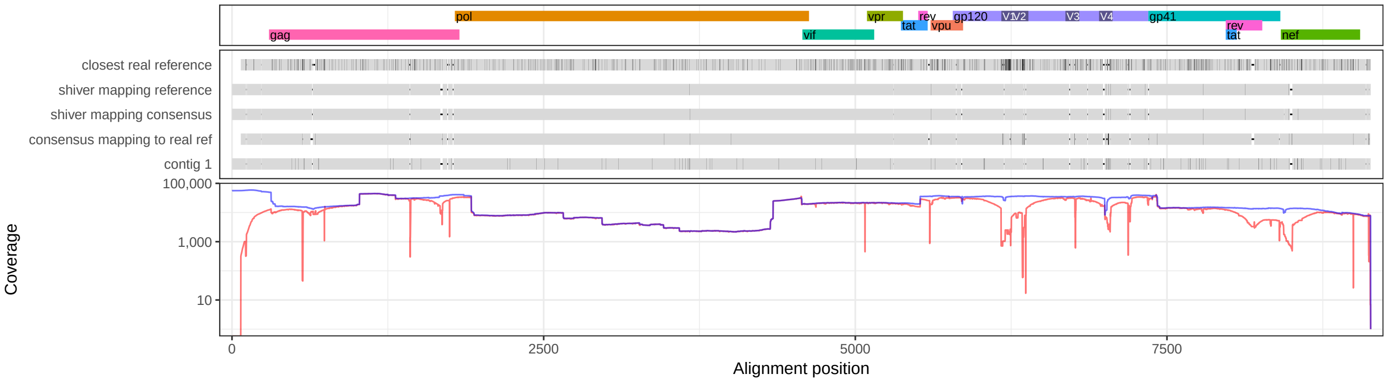

Figure S33: ERR732095 sequences and coverage (mapping to the `shiver` reference in blue, to the real reference in red).

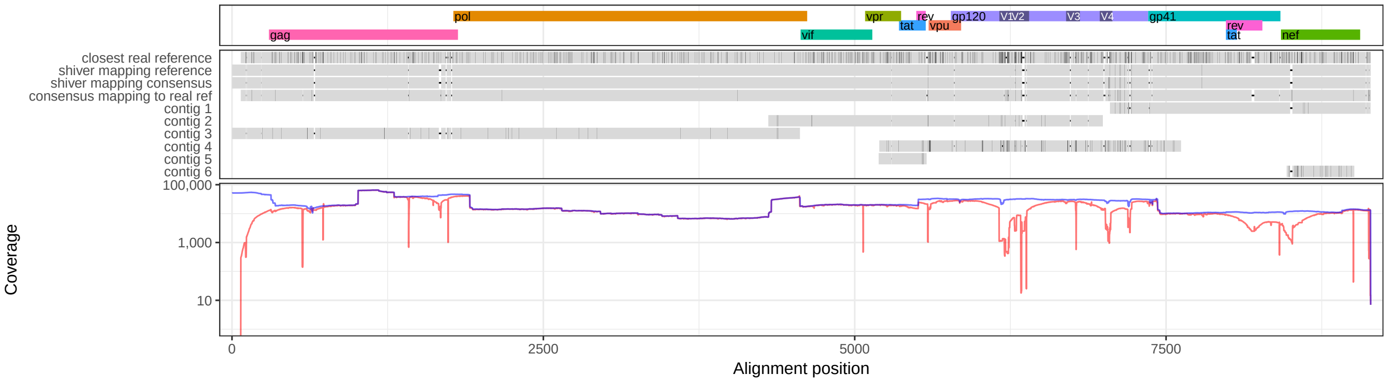

Figure S34: ERR732096 sequences and coverage (mapping to the `shiver` reference in blue, to the real reference in red).

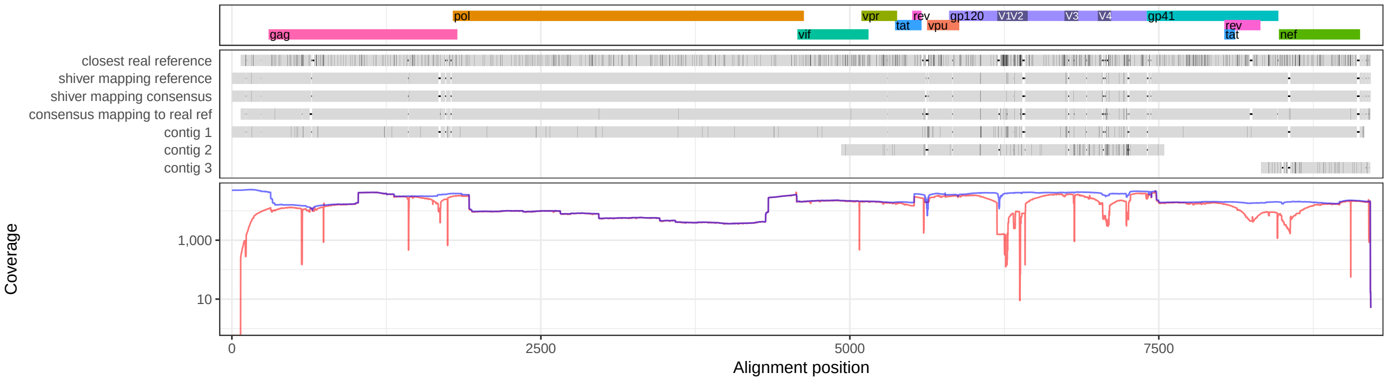

Figure S35: ERR732097 sequences and coverage (mapping to the `shiver` reference in blue, to the real reference in red).

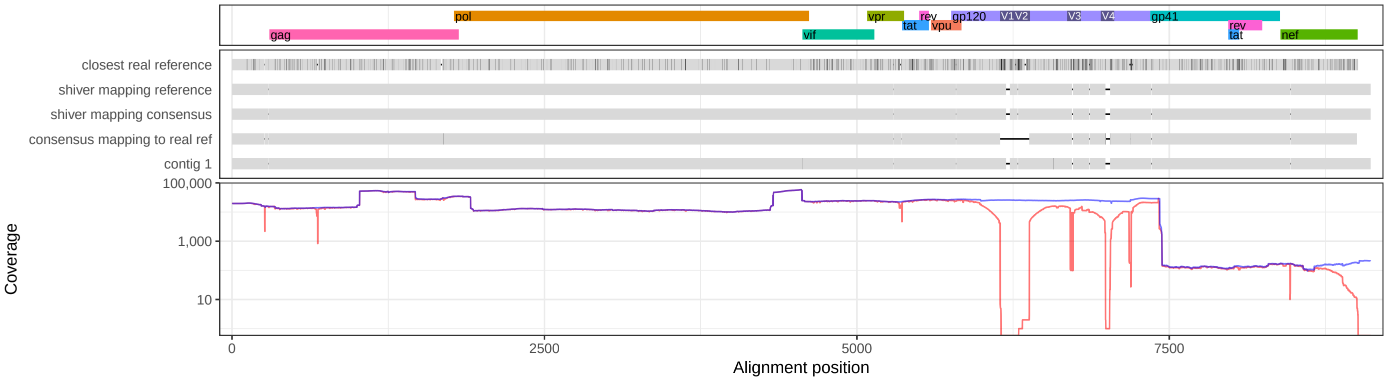

Figure S36: ERR732098 sequences and coverage (mapping to the `shiver` reference in blue, to the real reference in red).

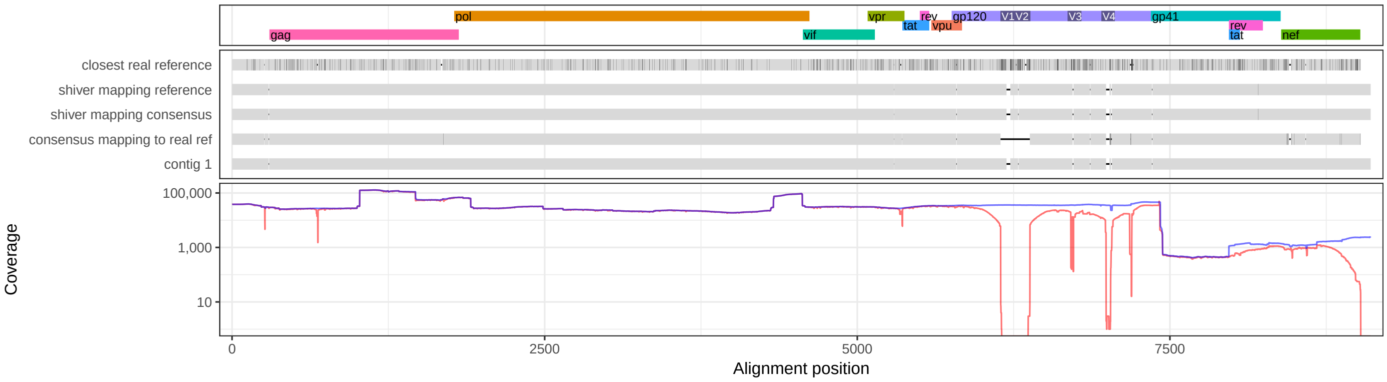

Figure S37: ERR732099 sequences and coverage (mapping to the `shiver` reference in blue, to the real reference in red).

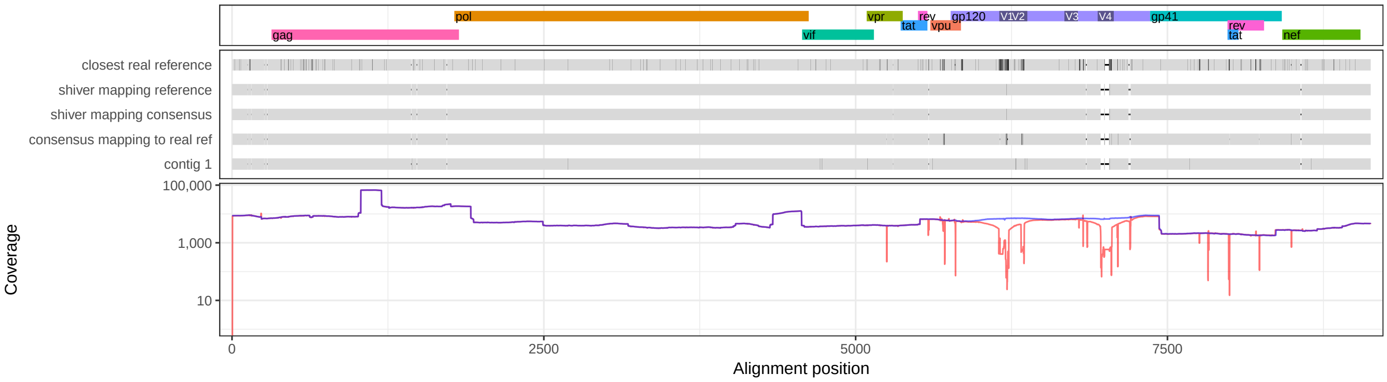

Figure S38: ERR732100 sequences and coverage (mapping to the `shiver` reference in blue, to the real reference in red).

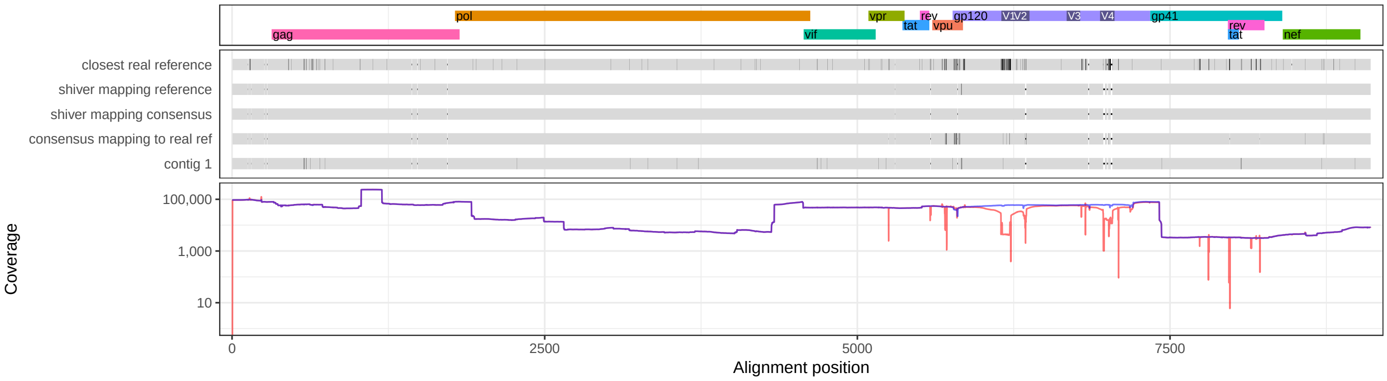

Figure S39: ERR732101 sequences and coverage (mapping to the shiver reference in blue, to the real reference in red).

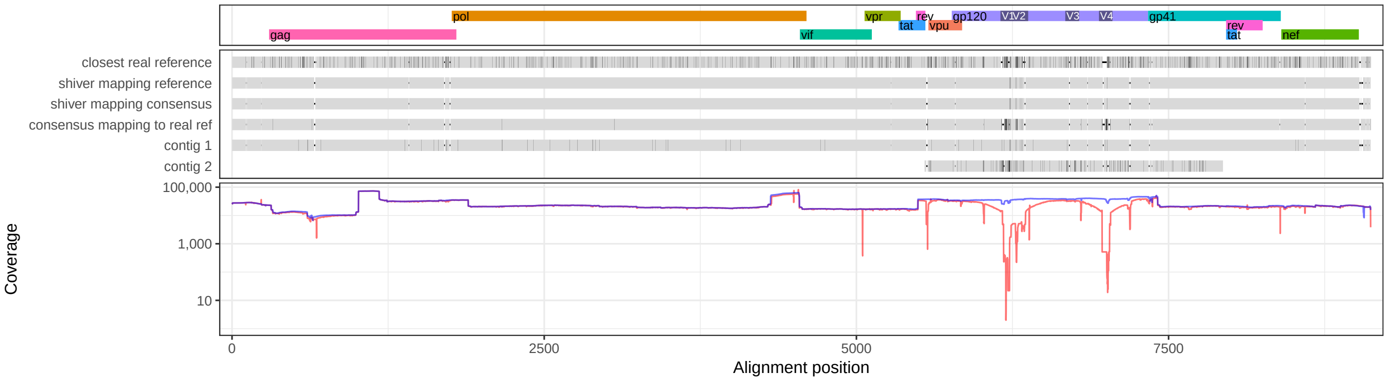

Figure S40: ERR732102 sequences and coverage (mapping to the shiver reference in blue, to the real reference in red).

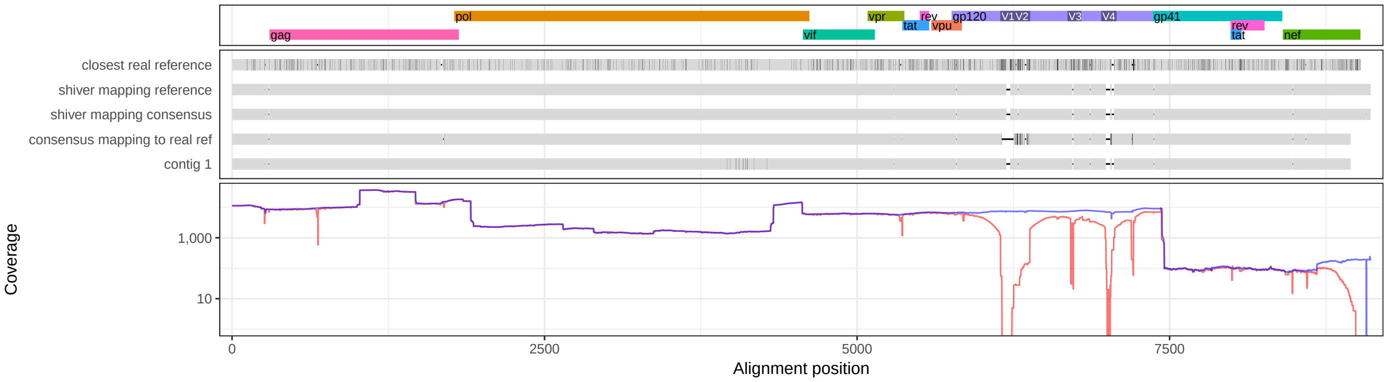

Figure S41: ERR732103 sequences and coverage (mapping to the shiver reference in blue, to the real reference in red).

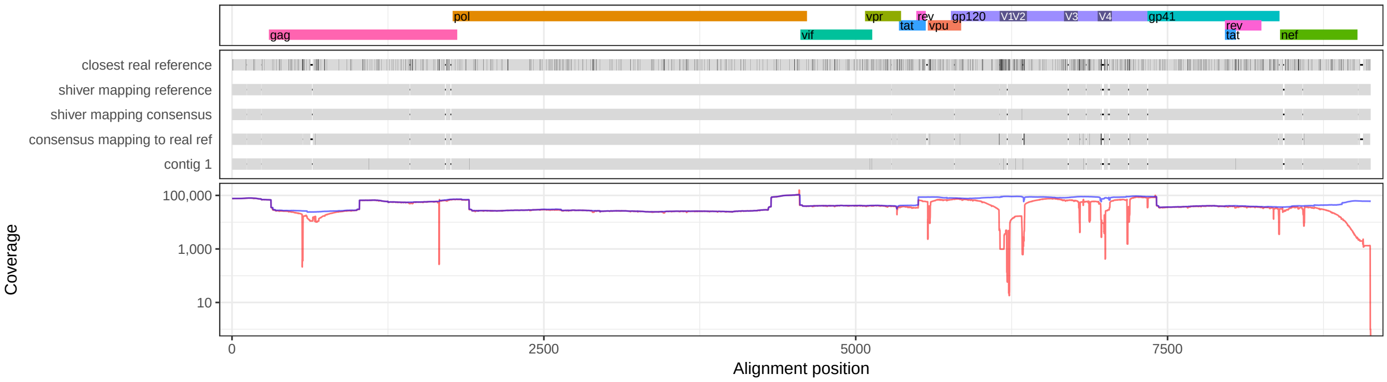

Figure S42: ERR732104 sequences and coverage (mapping to the shiver reference in blue, to the real reference in red).

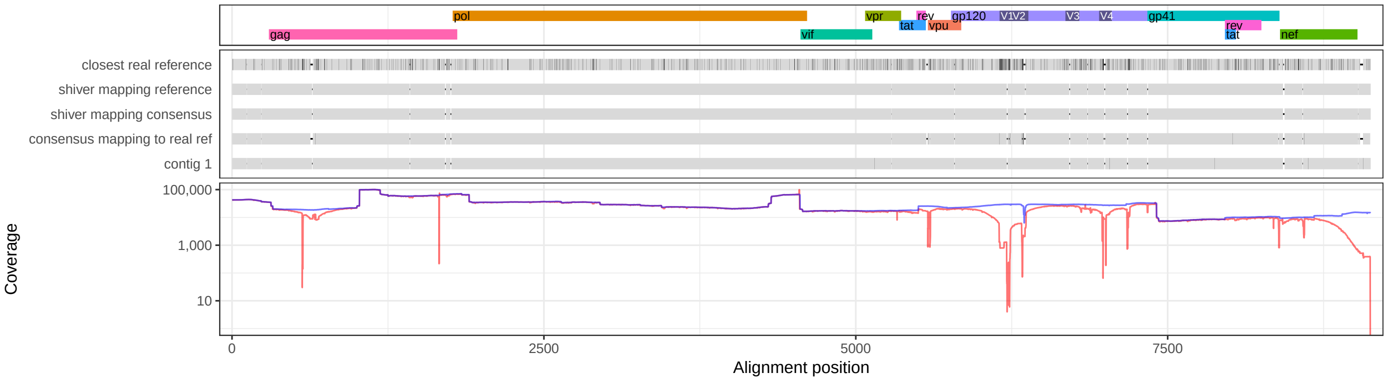

Figure S43: ERR732105 sequences and coverage (mapping to the `shiver` reference in blue, to the real reference in red).

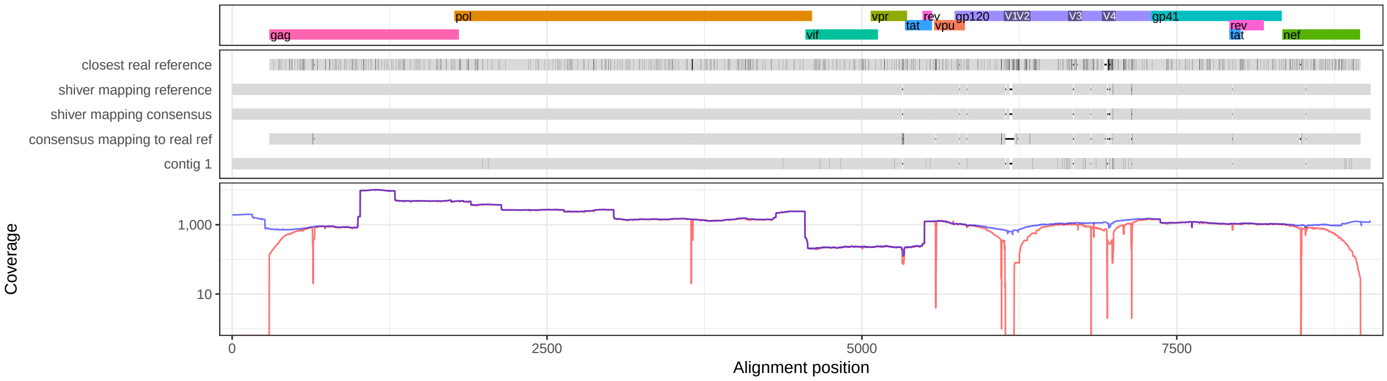

Figure S44: ERR732106 sequences and coverage (mapping to the `shiver` reference in blue, to the real reference in red).

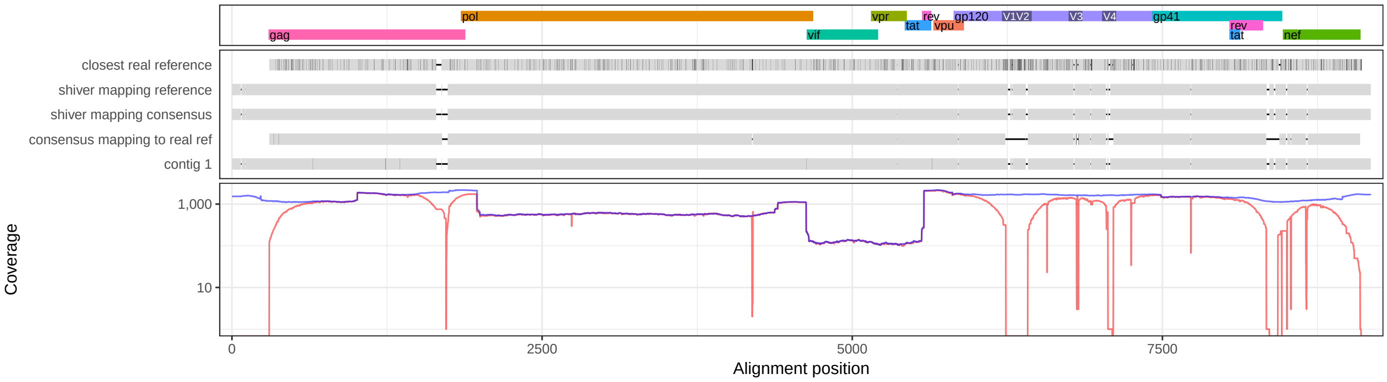

Figure S45: ERR732107 sequences and coverage (mapping to the `shiver` reference in blue, to the real reference in red).

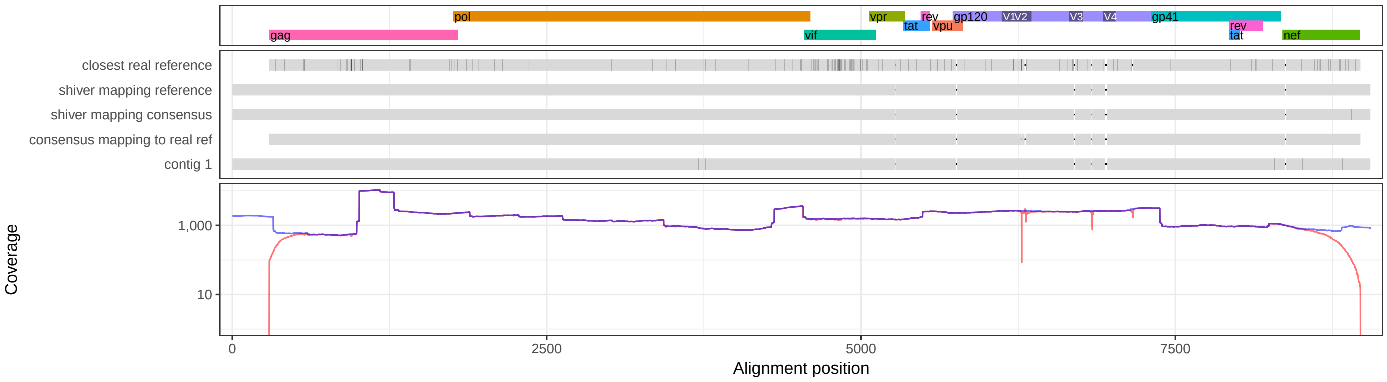

Figure S46: ERR732108 sequences and coverage (mapping to the `shiver` reference in blue, to the real reference in red).

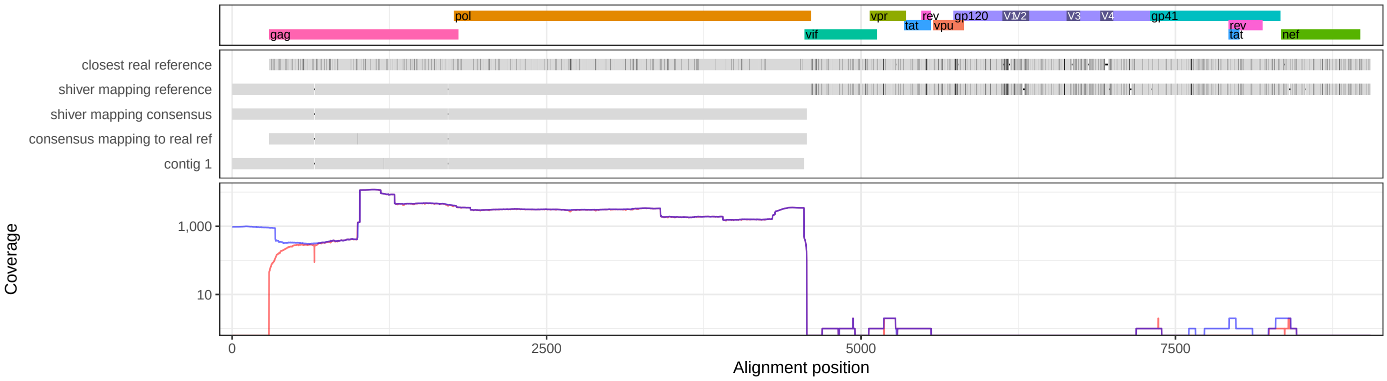

Figure S47: ERR732109 sequences and coverage (mapping to the `shiver` reference in blue, to the real reference in red).

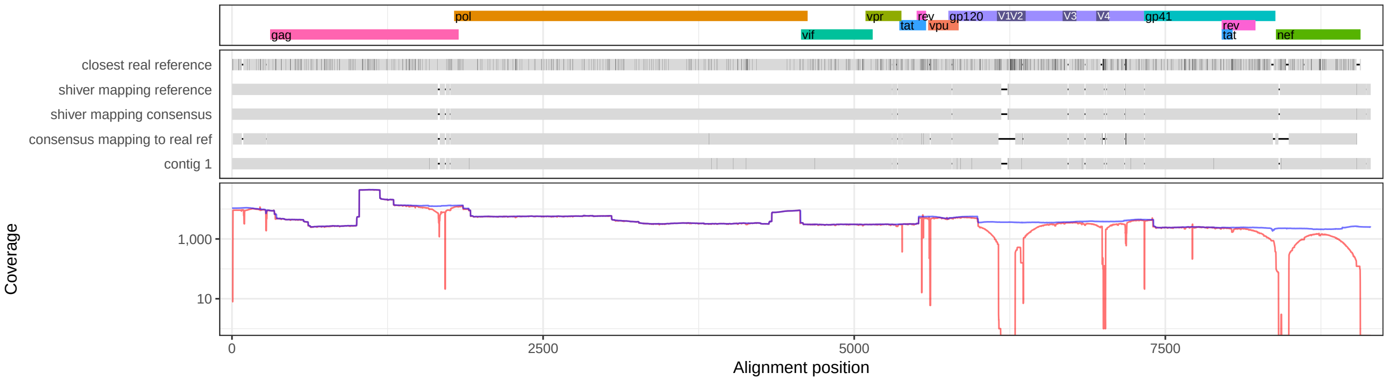

Figure S48: ERR732110 sequences and coverage (mapping to the `shiver` reference in blue, to the real reference in red).

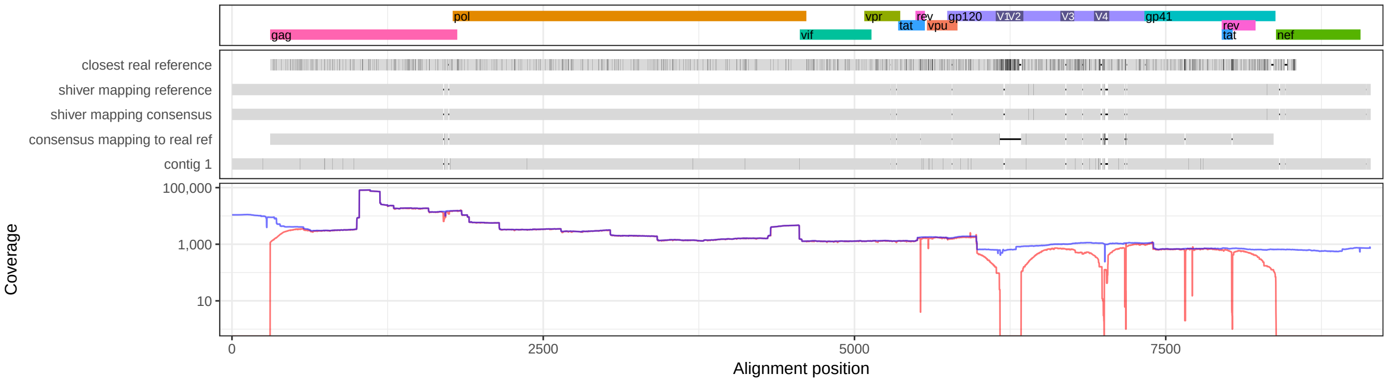

Figure S49: ERR732111 sequences and coverage (mapping to the `shiver` reference in blue, to the real reference in red).

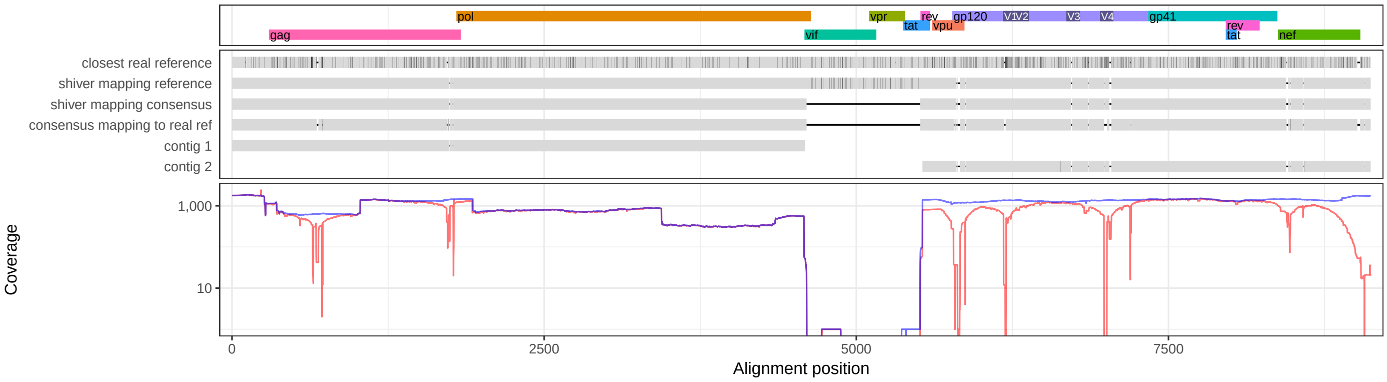

Figure S50: ERR732112 sequences and coverage (mapping to the `shiver` reference in blue, to the real reference in red).

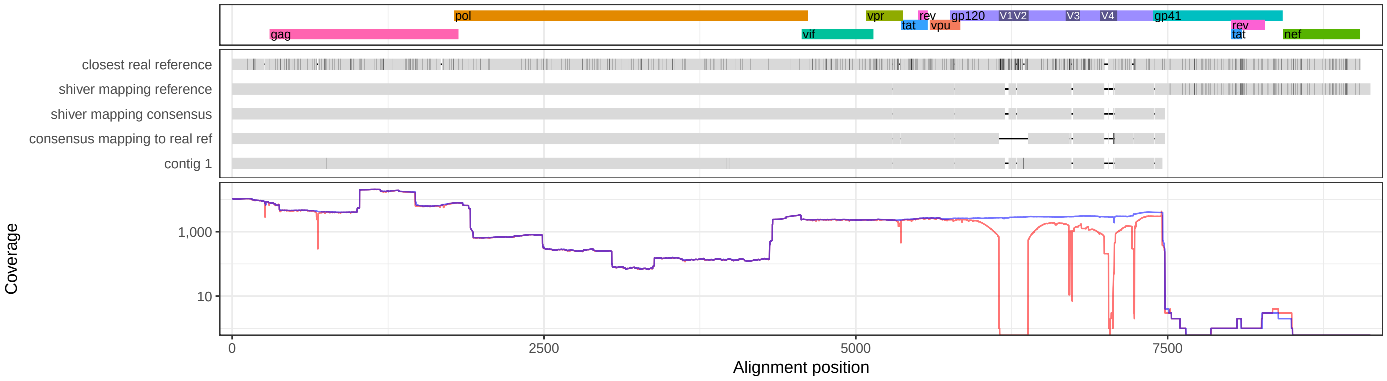

Figure S51: ERR732113 sequences and coverage (mapping to the shiver reference in blue, to the real reference in red).

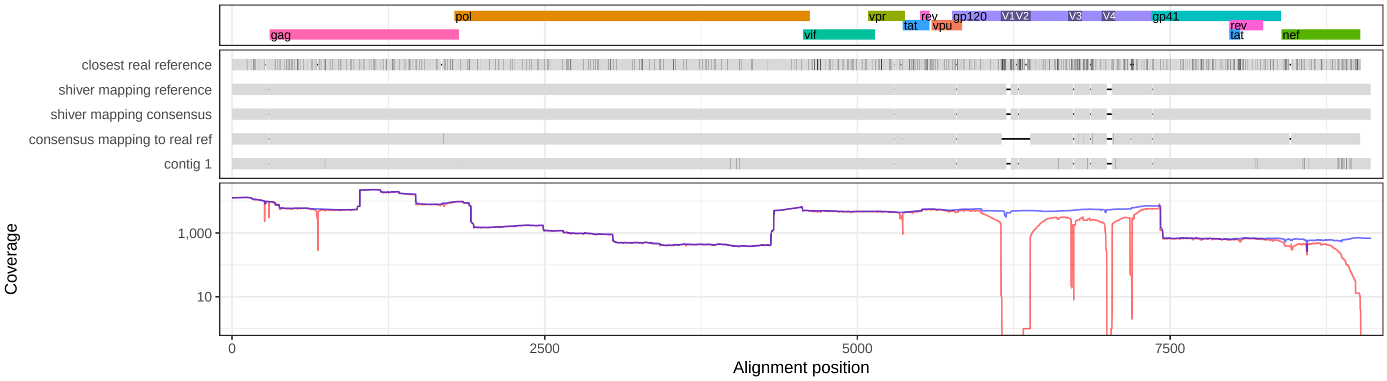

Figure S52: ERR732114 sequences and coverage (mapping to the shiver reference in blue, to the real reference in red).

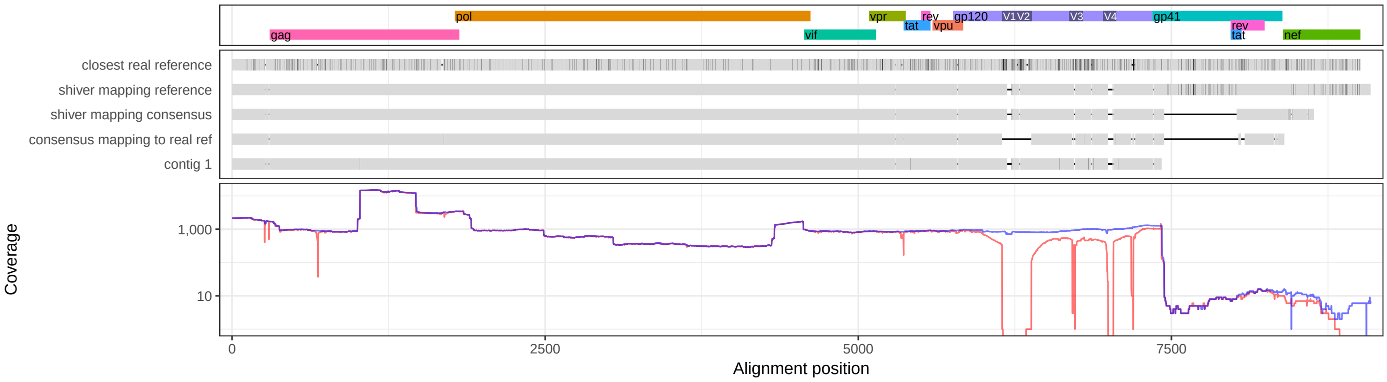

Figure S53: ERR732115 sequences and coverage (mapping to the shiver reference in blue, to the real reference in red).

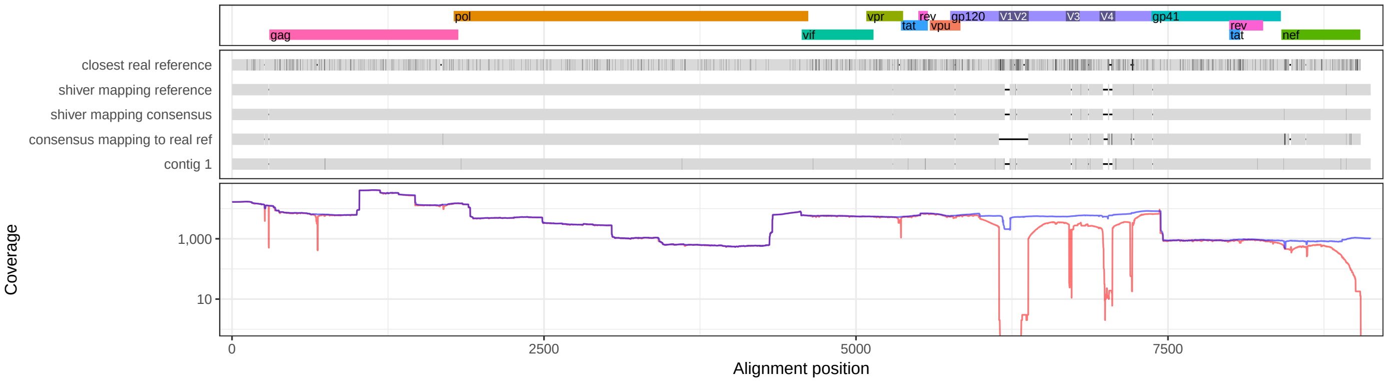

Figure S54: ERR732116 sequences and coverage (mapping to the shiver reference in blue, to the real reference in red).

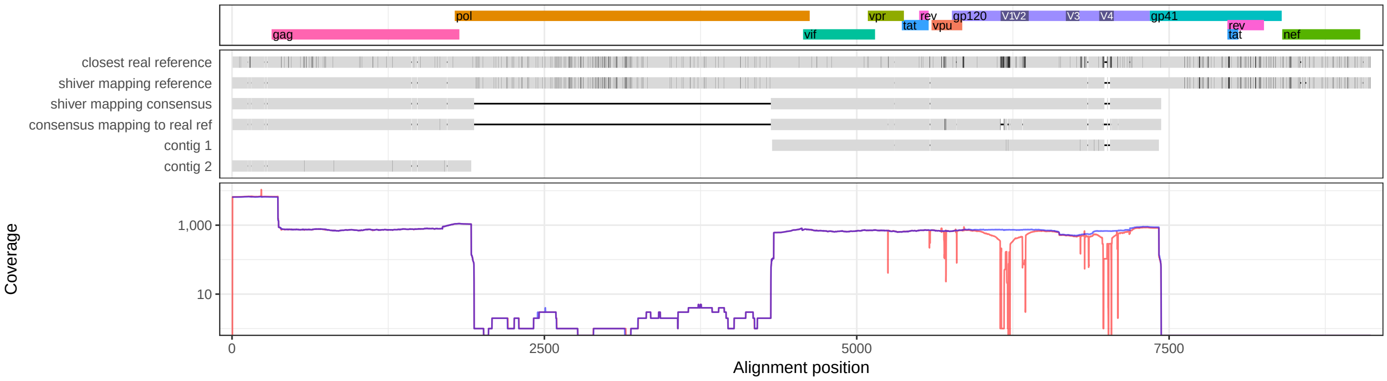

Figure S55: ERR732117 sequences and coverage (mapping to the `shiver` reference in blue, to the real reference in red).

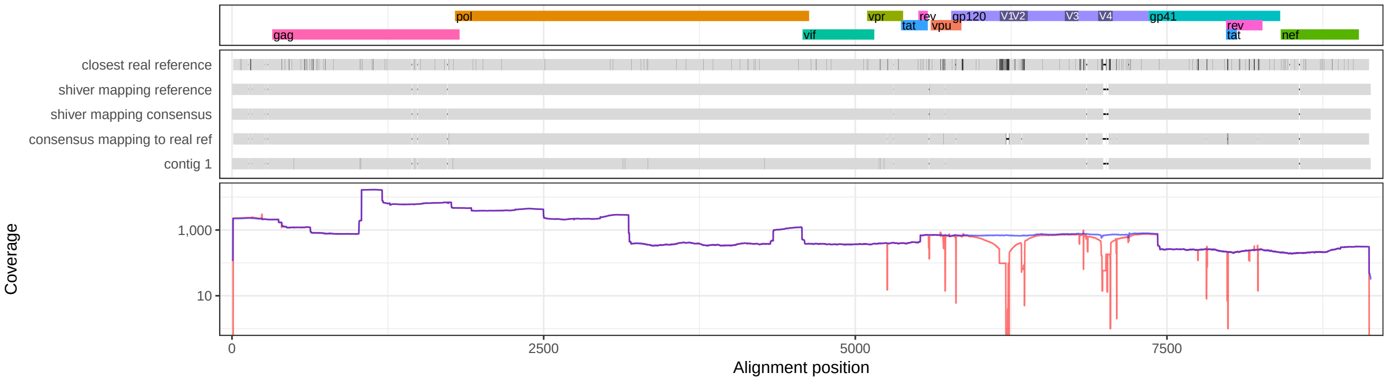

Figure S56: ERR732118 sequences and coverage (mapping to the `shiver` reference in blue, to the real reference in red).

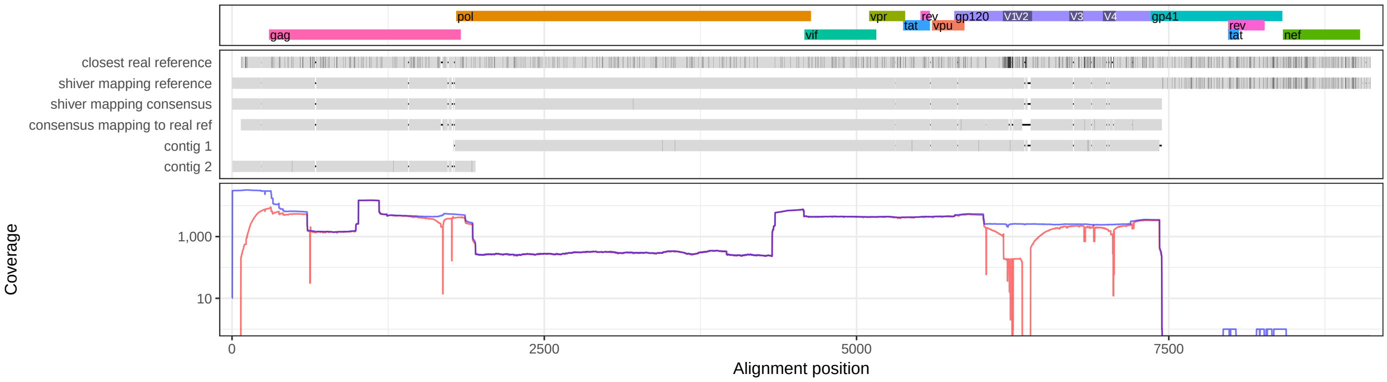

Figure S57: ERR732119 sequences and coverage (mapping to the `shiver` reference in blue, to the real reference in red).

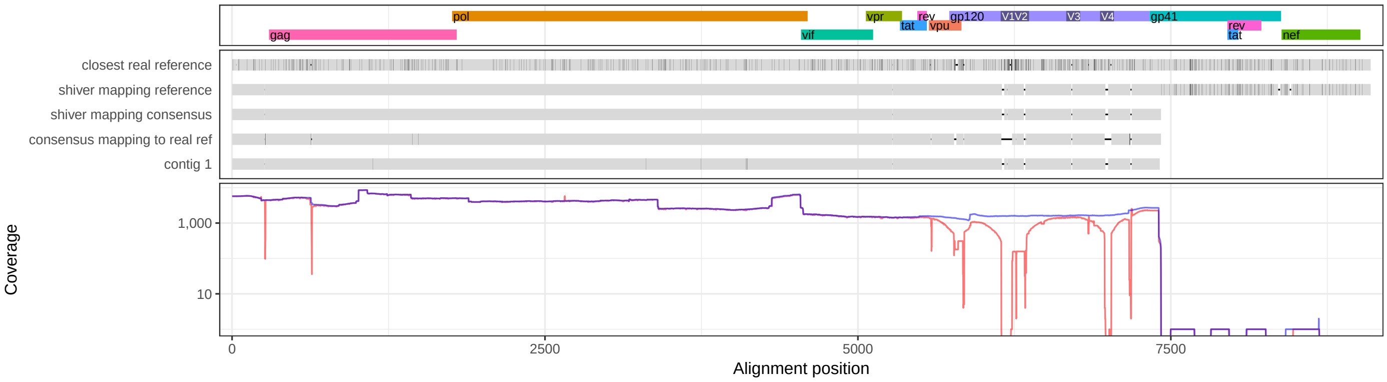

Figure S58: ERR732120 sequences and coverage (mapping to the `shiver` reference in blue, to the real reference in red).

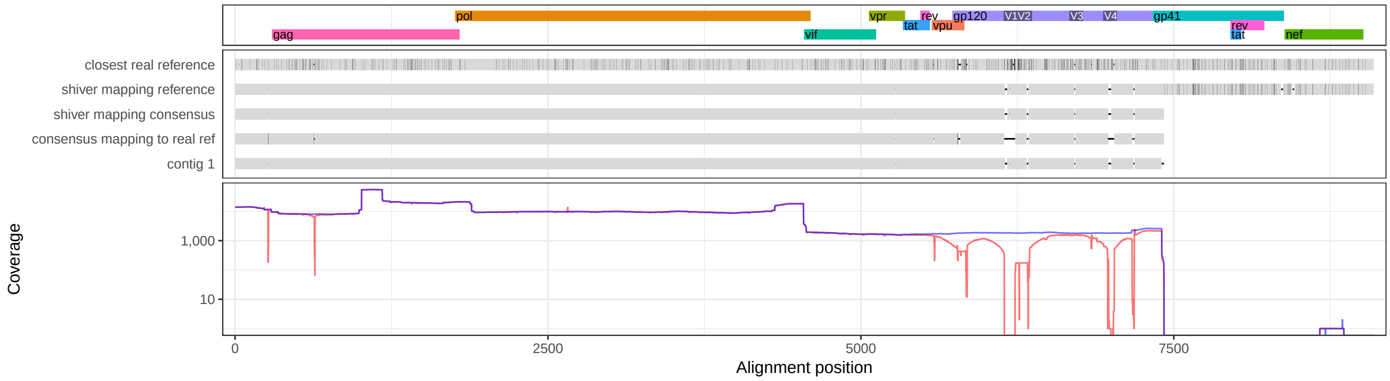

Figure S59: ERR732121 sequences and coverage (mapping to the shiver reference in blue, to the real reference in red).

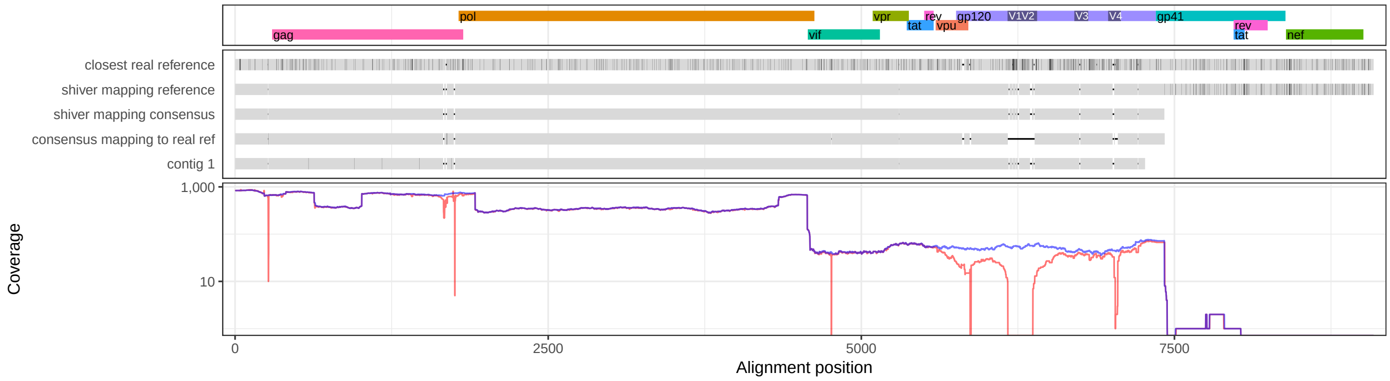

Figure S60: ERR732122 sequences and coverage (mapping to the shiver reference in blue, to the real reference in red).

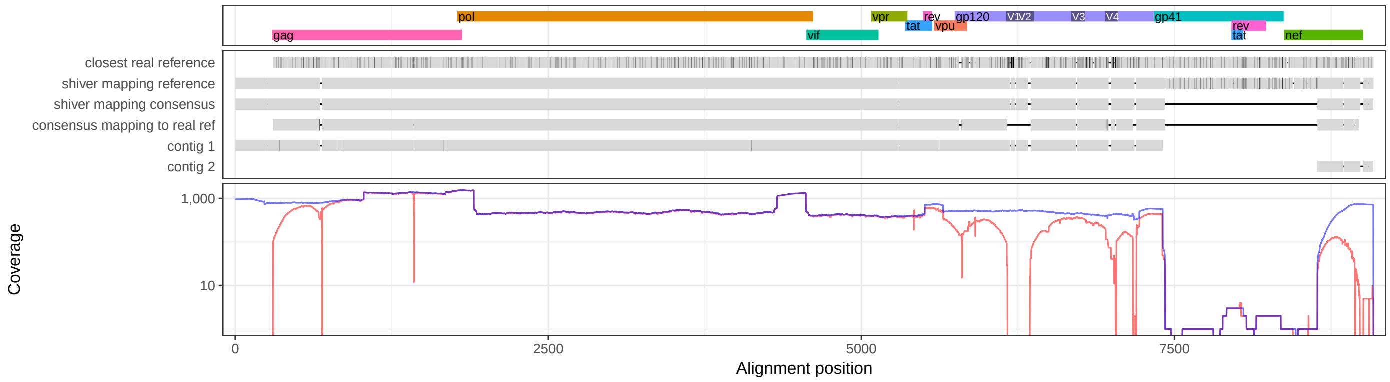

Figure S61: ERR732123 sequences and coverage (mapping to the shiver reference in blue, to the real reference in red).

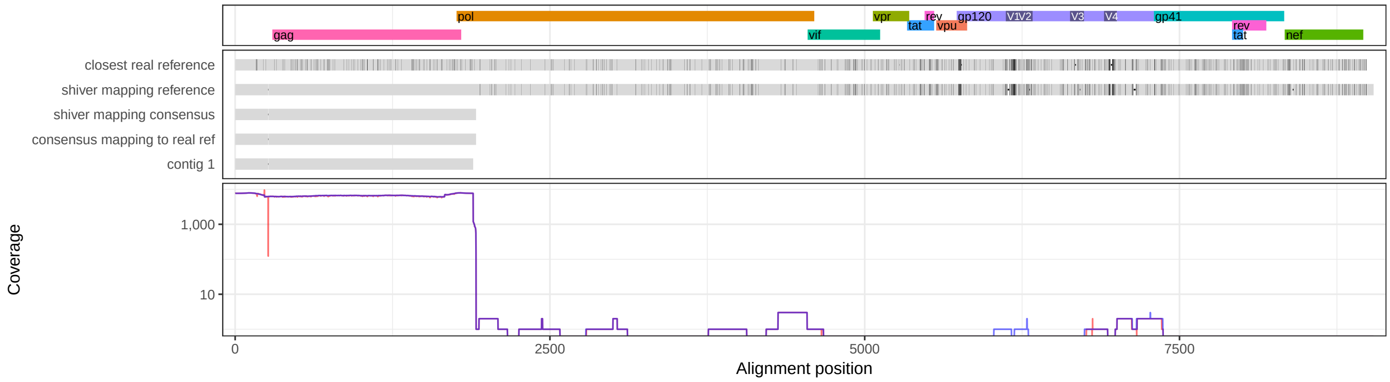

Figure S62: ERR732124 sequences and coverage (mapping to the shiver reference in blue, to the real reference in red).

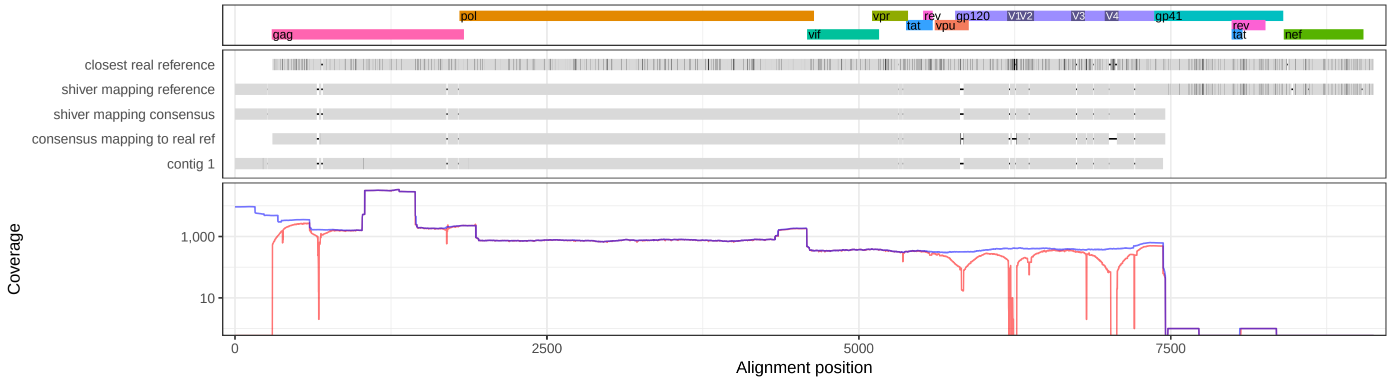

Figure S63: ERR732126 sequences and coverage (mapping to the `shiver` reference in blue, to the real reference in red).

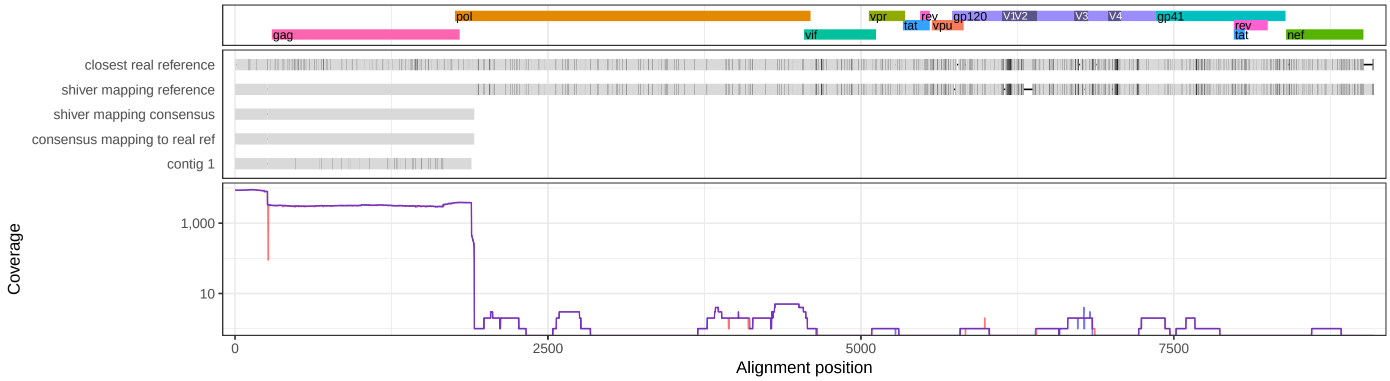

Figure S64: ERR732127 sequences and coverage (mapping to the `shiver` reference in blue, to the real reference in red).

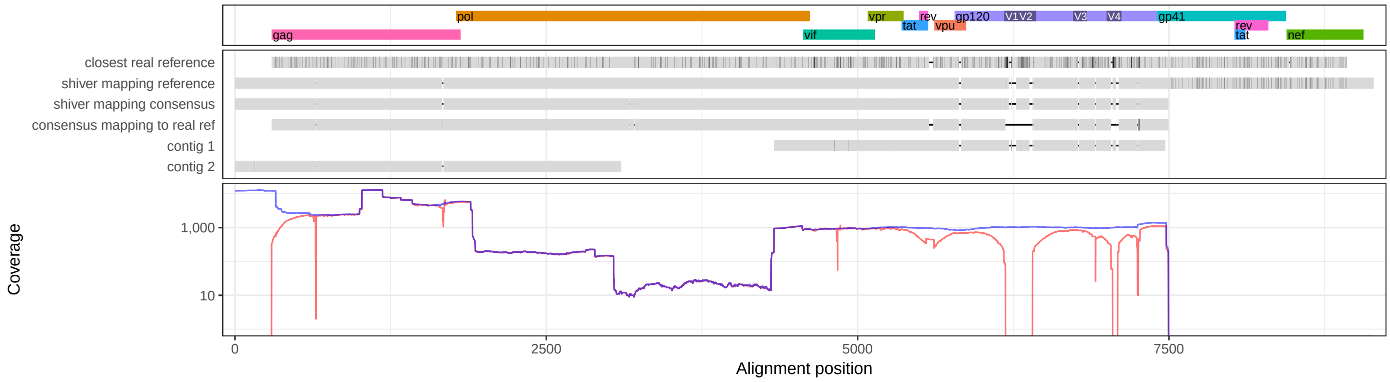

Figure S65: ERR732128 sequences and coverage (mapping to the `shiver` reference in blue, to the real reference in red).

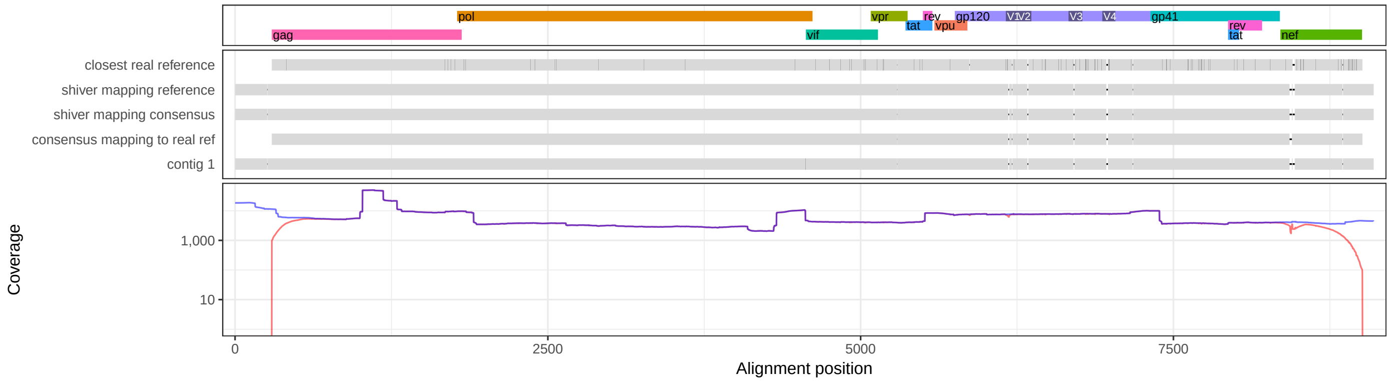

Figure S66: ERR732129 sequences and coverage (mapping to the `shiver` reference in blue, to the real reference in red).

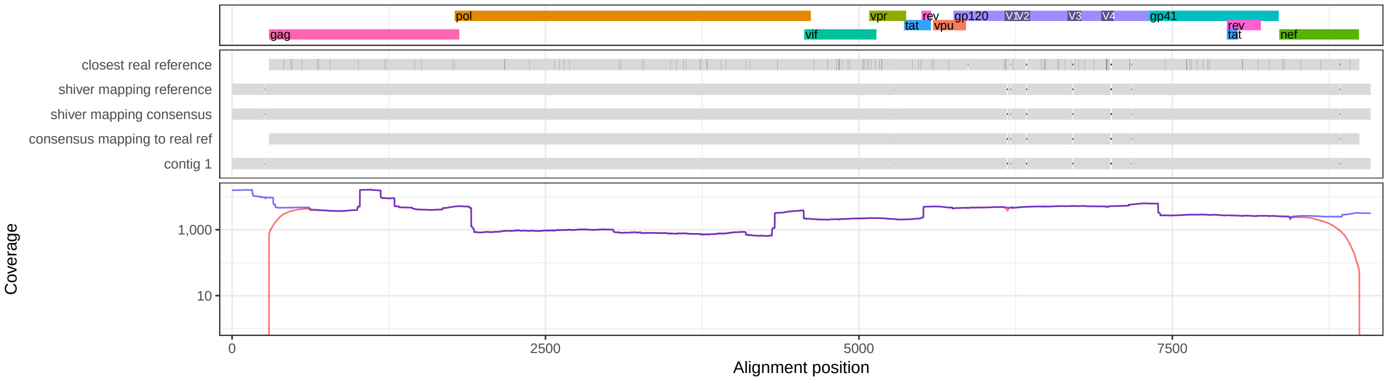

Figure S67: ERR732130 sequences and coverage (mapping to the `shiver` reference in blue, to the real reference in red).

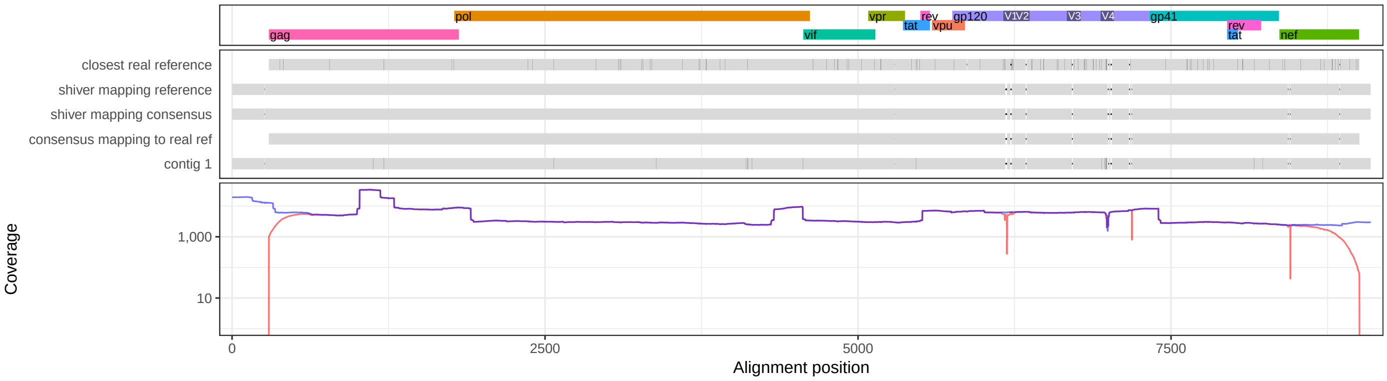

Figure S68: ERR732131 sequences and coverage (mapping to the `shiver` reference in blue, to the real reference in red).

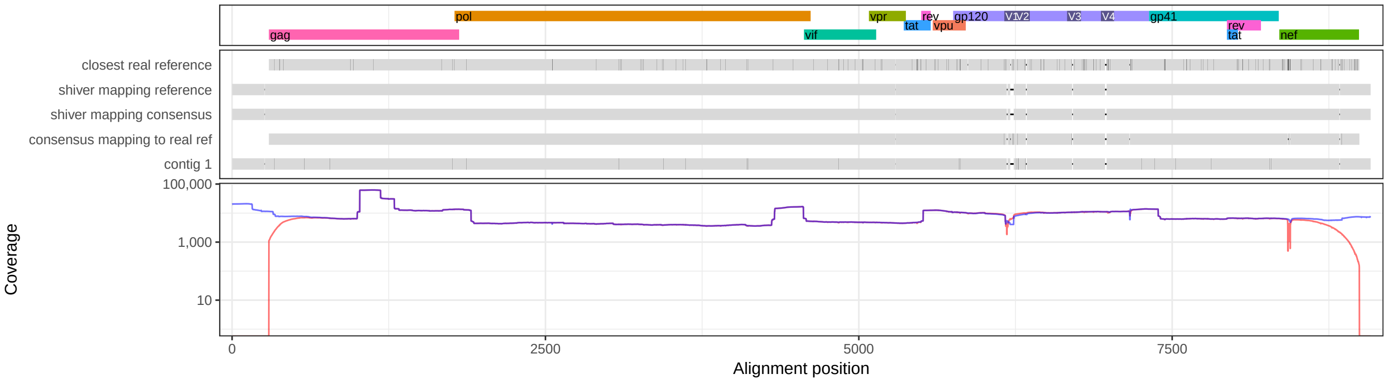

Figure S69: ERR732132 sequences and coverage (mapping to the `shiver` reference in blue, to the real reference in red).

SI 5 Sequences and Coverage by Sample: Hiseq Data

Plots of the same format as those described in Supplementary Information section SI 4, for the Hiseq data.

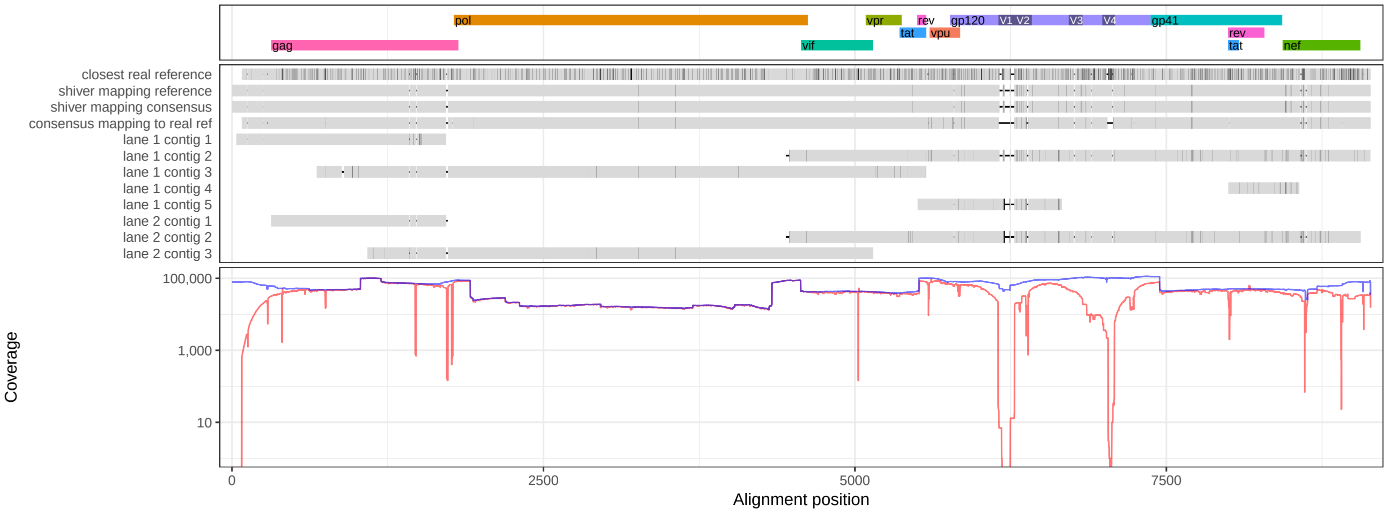

Figure S70: 17621_3_80 sequences and coverage (mapping to the shiver reference in blue, to the real reference in red).

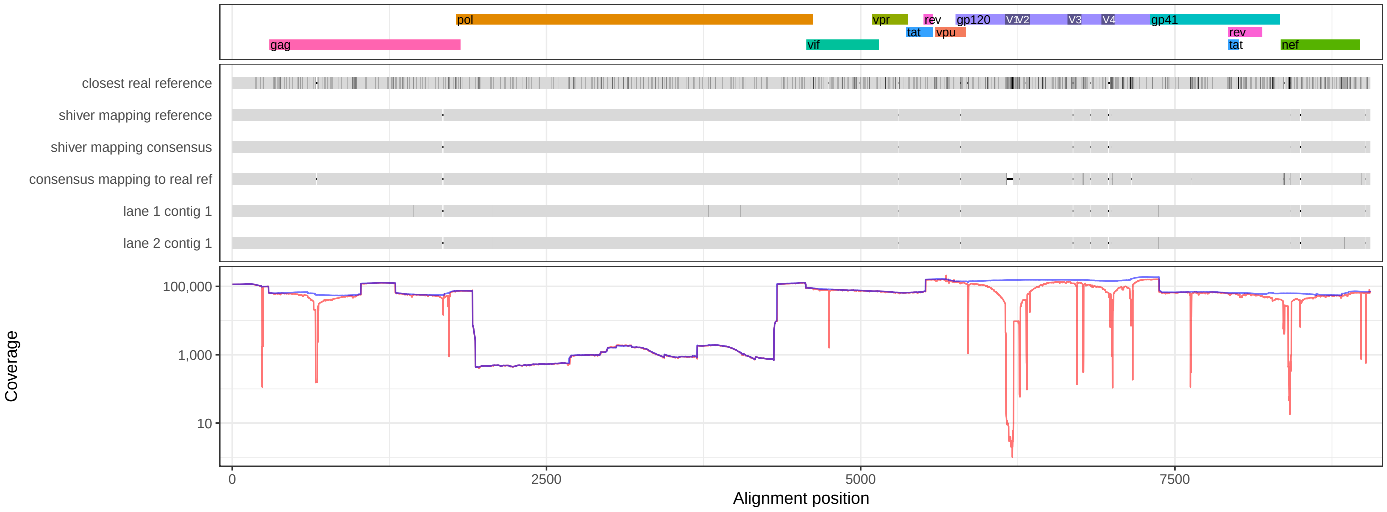

Figure S71: 17653_3_25 sequences and coverage (mapping to the shiver reference in blue, to the real reference in red).

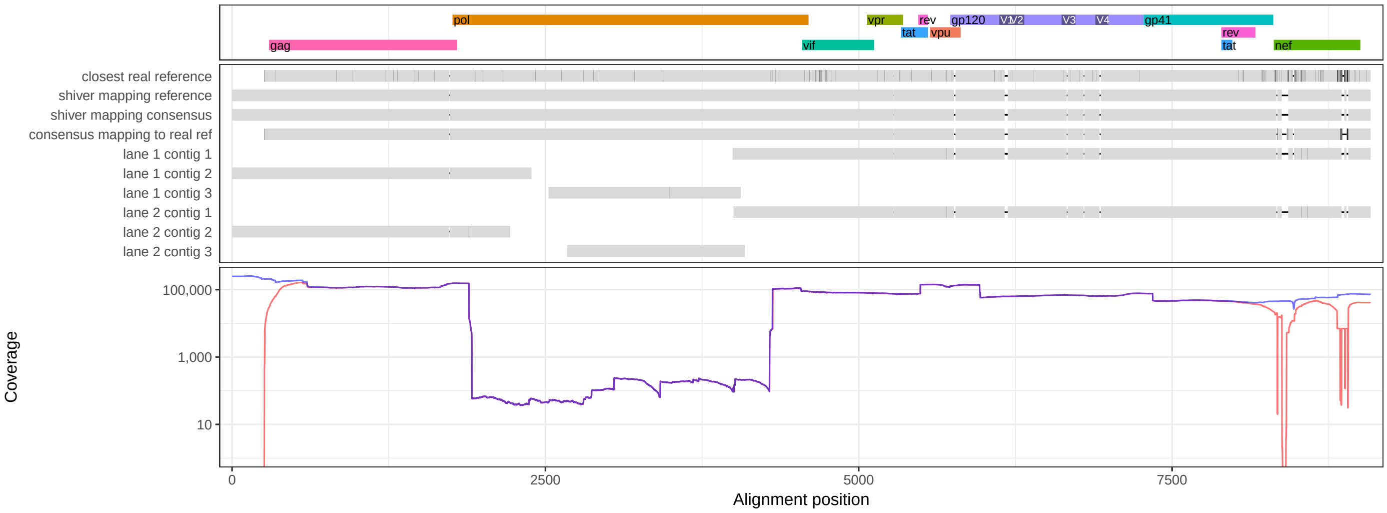

Figure S72: 17653_3_36 sequences and coverage (mapping to the shiver reference in blue, to the real reference in red).

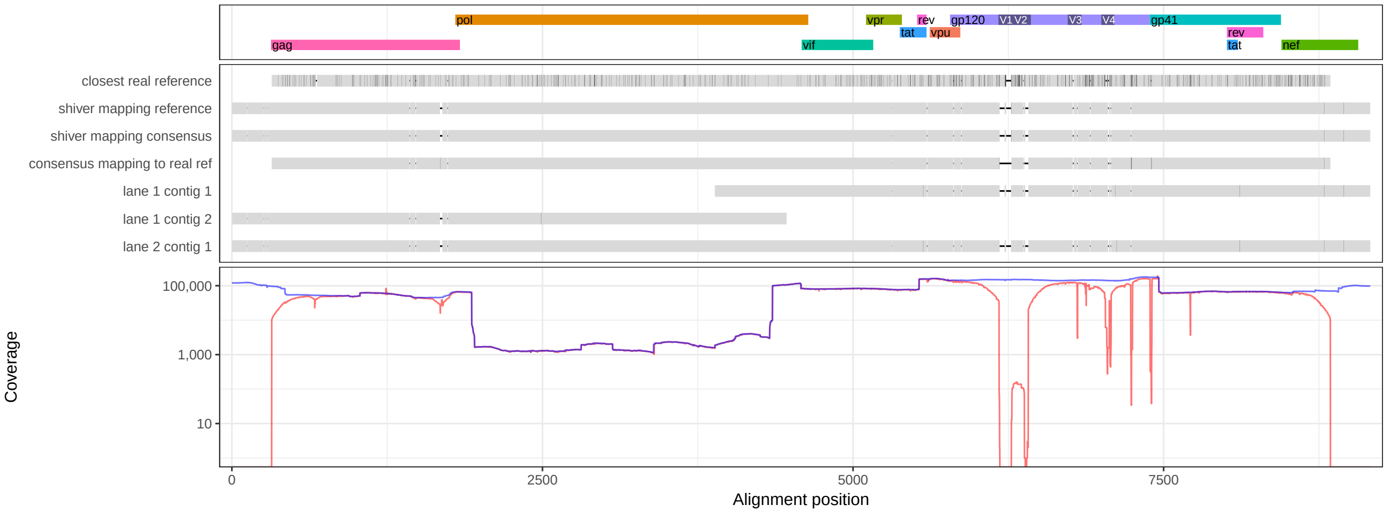

Figure S73: 17653_3_56 sequences and coverage (mapping to the `shiver` reference in blue, to the real reference in red).

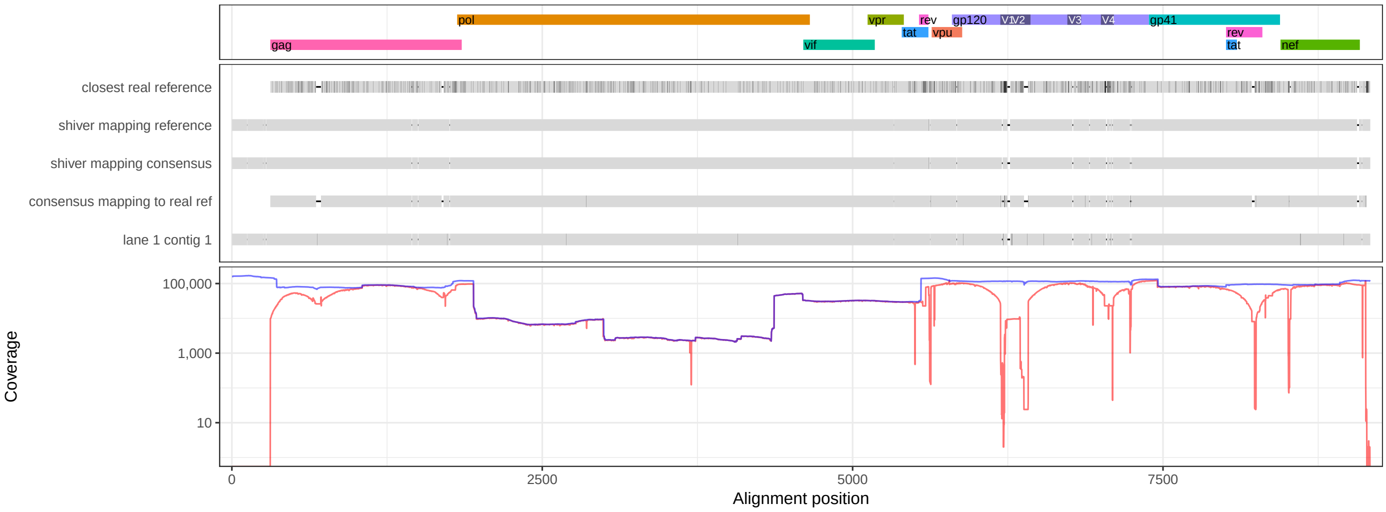

Figure S74: 17653_3_62 sequences and coverage (mapping to the `shiver` reference in blue, to the real reference in red).

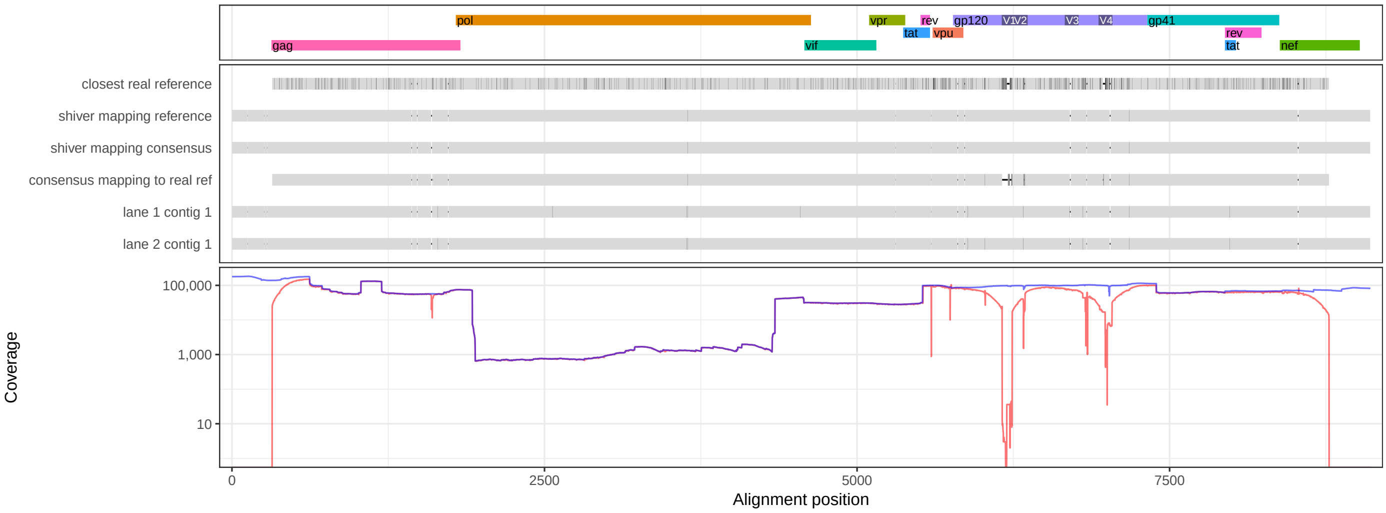

Figure S75: 17653_3_64 sequences and coverage (mapping to the `shiver` reference in blue, to the real reference in red).

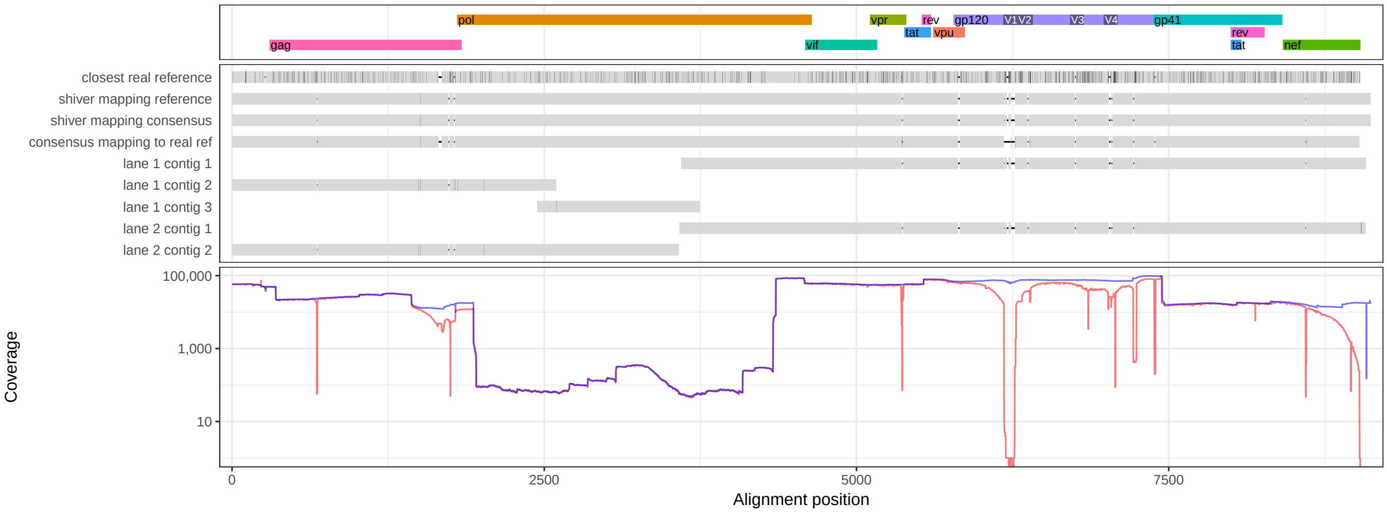

Figure S76: 17653_3_72 sequences and coverage (mapping to the `shiver` reference in blue, to the real reference in red).

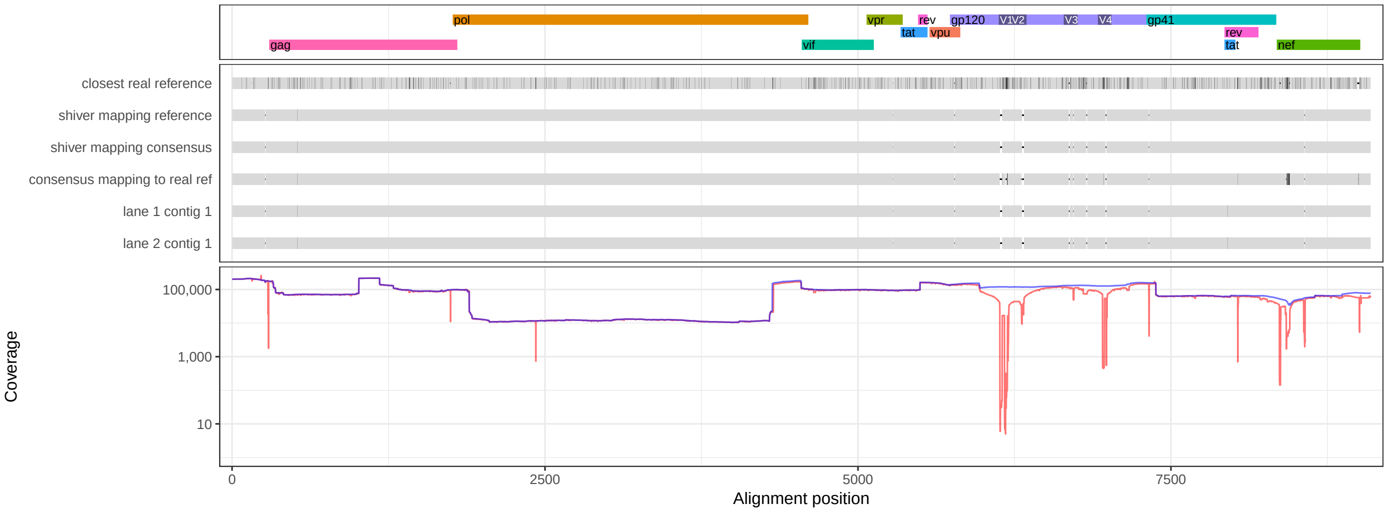

Figure S77: 17653_3_74 sequences and coverage (mapping to the `shiver` reference in blue, to the real reference in red).

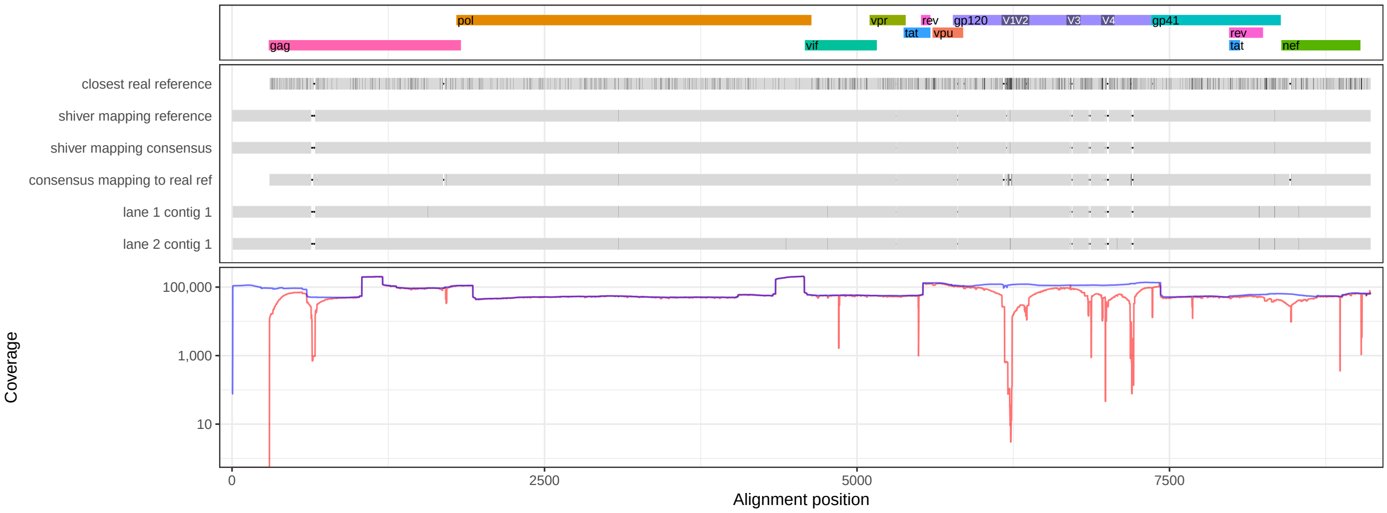

Figure S78: 17654_3_46 sequences and coverage (mapping to the `shiver` reference in blue, to the real reference in red).

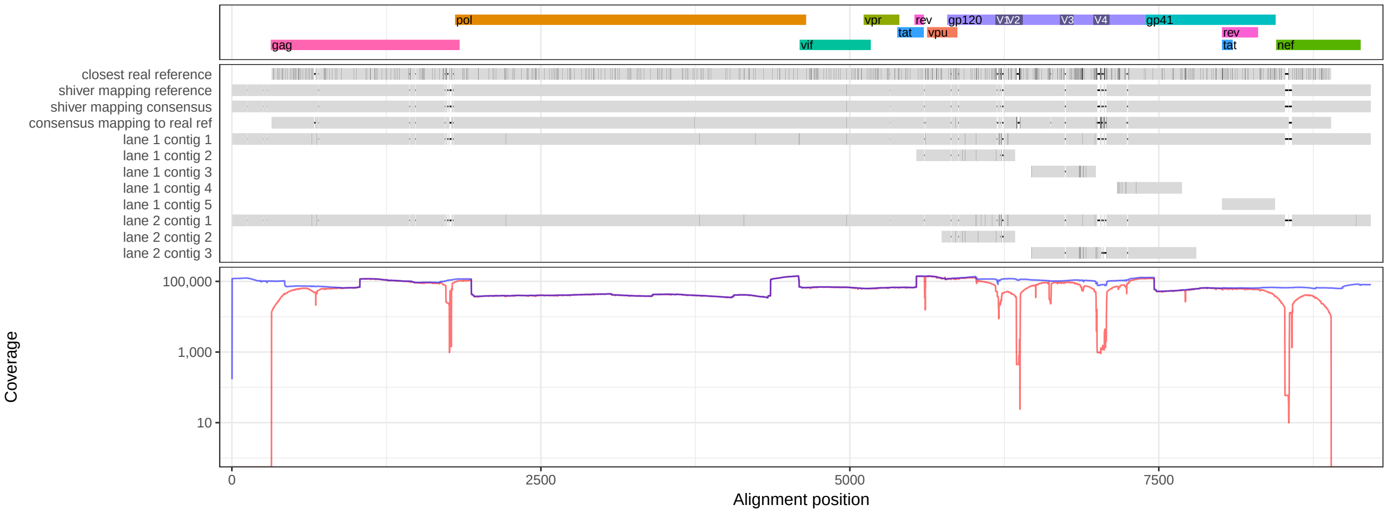

Figure S79: 17654_3_71 sequences and coverage (mapping to the shiver reference in blue, to the real reference in red).

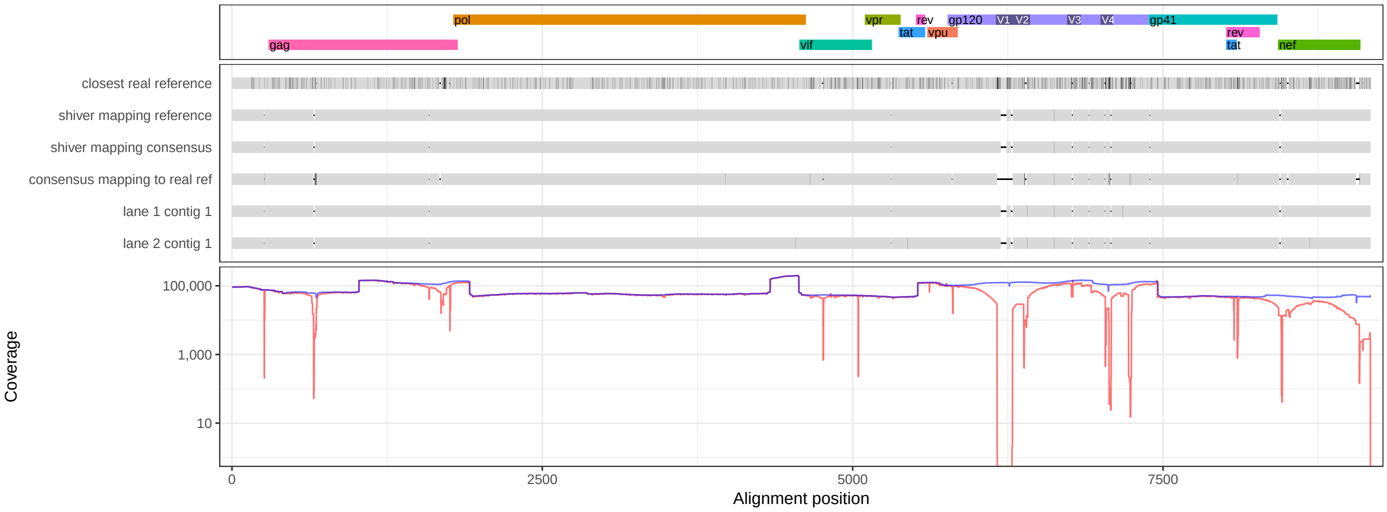

Figure S80: 17654_3_72 sequences and coverage (mapping to the shiver reference in blue, to the real reference in red).

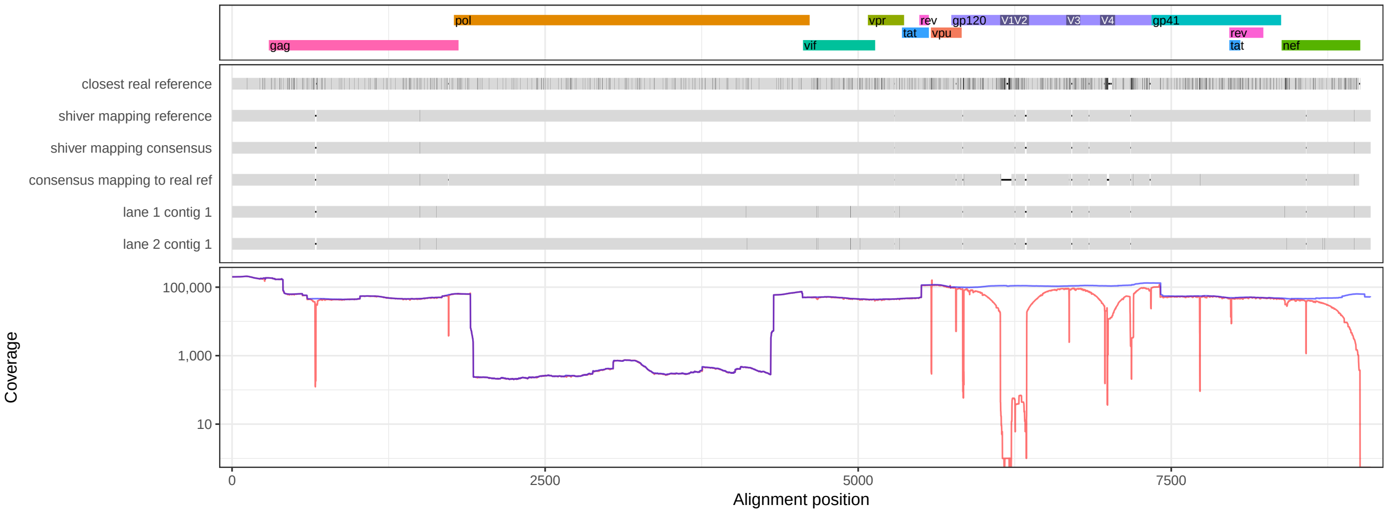

Figure S81: 17654_3_78 sequences and coverage (mapping to the shiver reference in blue, to the real reference in red).

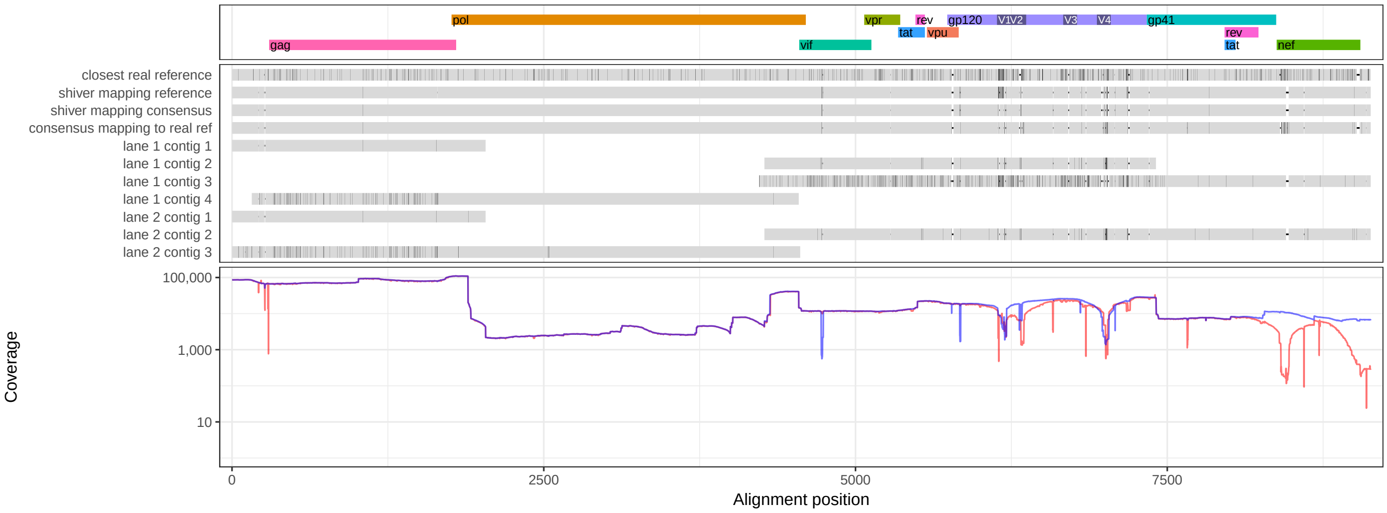

Figure S82: 17795_3_40 sequences and coverage (mapping to the `shiver` reference in blue, to the real reference in red).

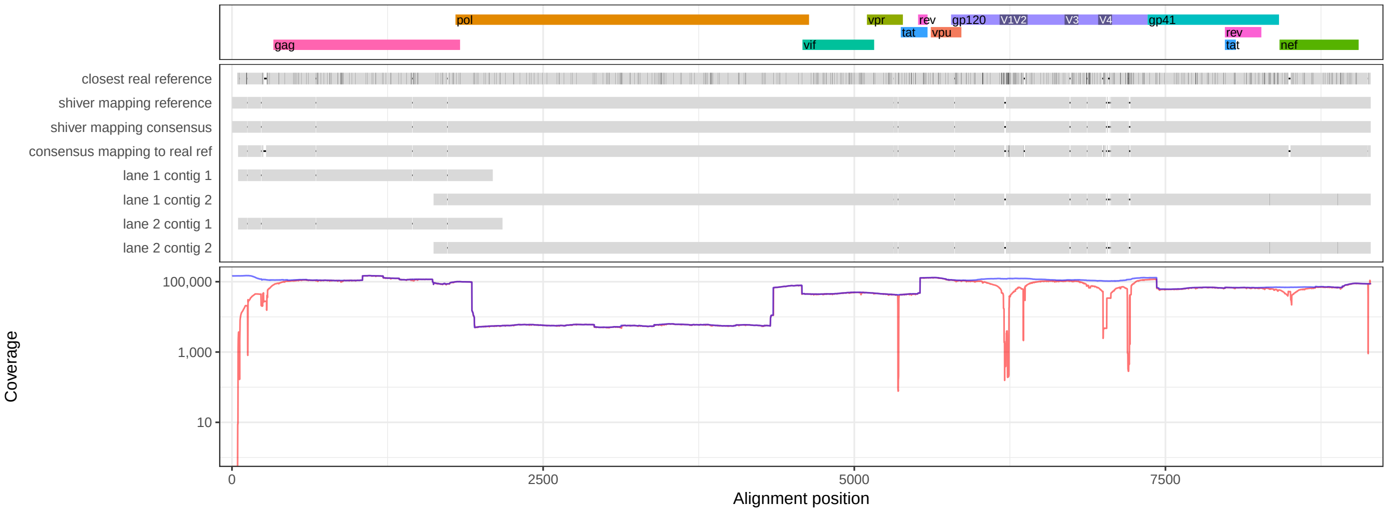

Figure S83: 17796_3_1 sequences and coverage (mapping to the `shiver` reference in blue, to the real reference in red).

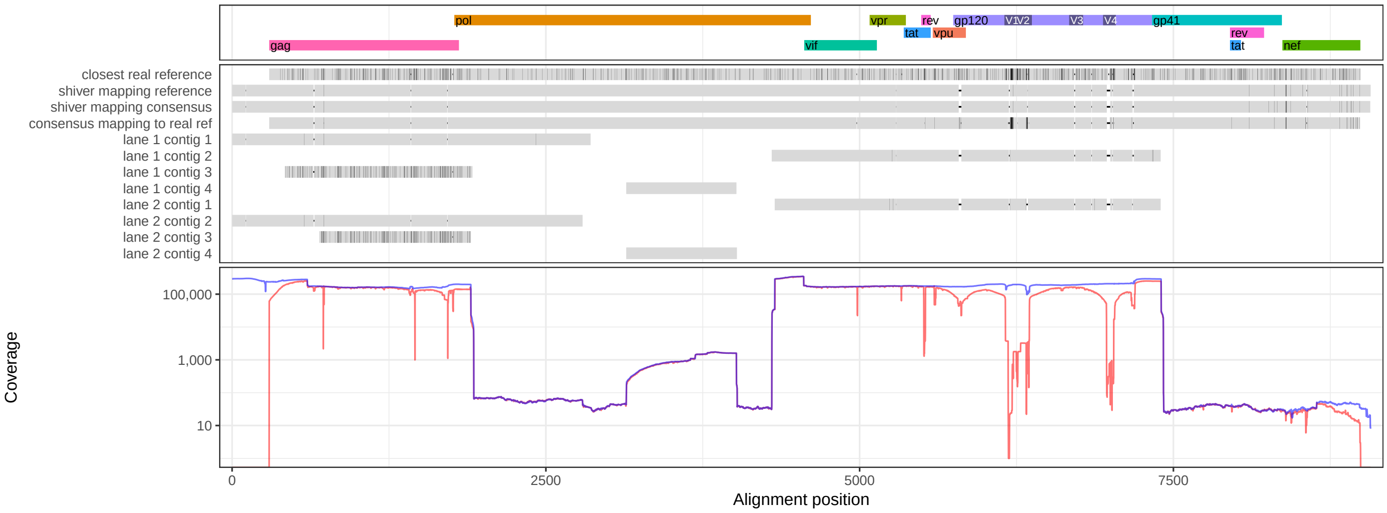

Figure S84: 17796_3_29 sequences and coverage (mapping to the `shiver` reference in blue, to the real reference in red).

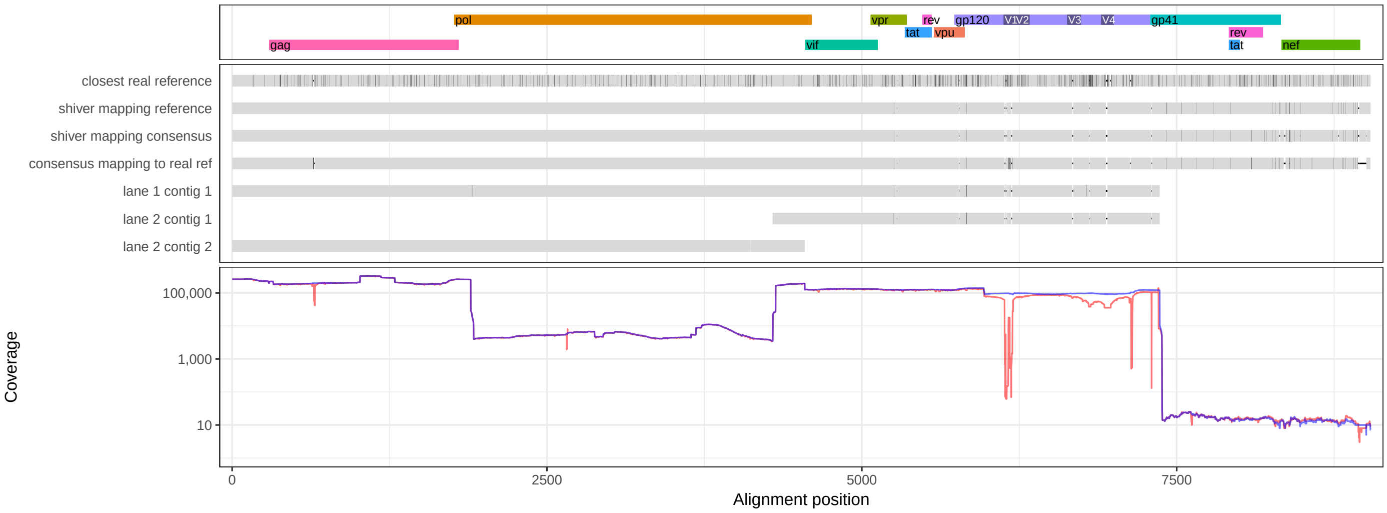

Figure S85: 17796_3_30 sequences and coverage (mapping to the `shiver` reference in blue, to the real reference in red).

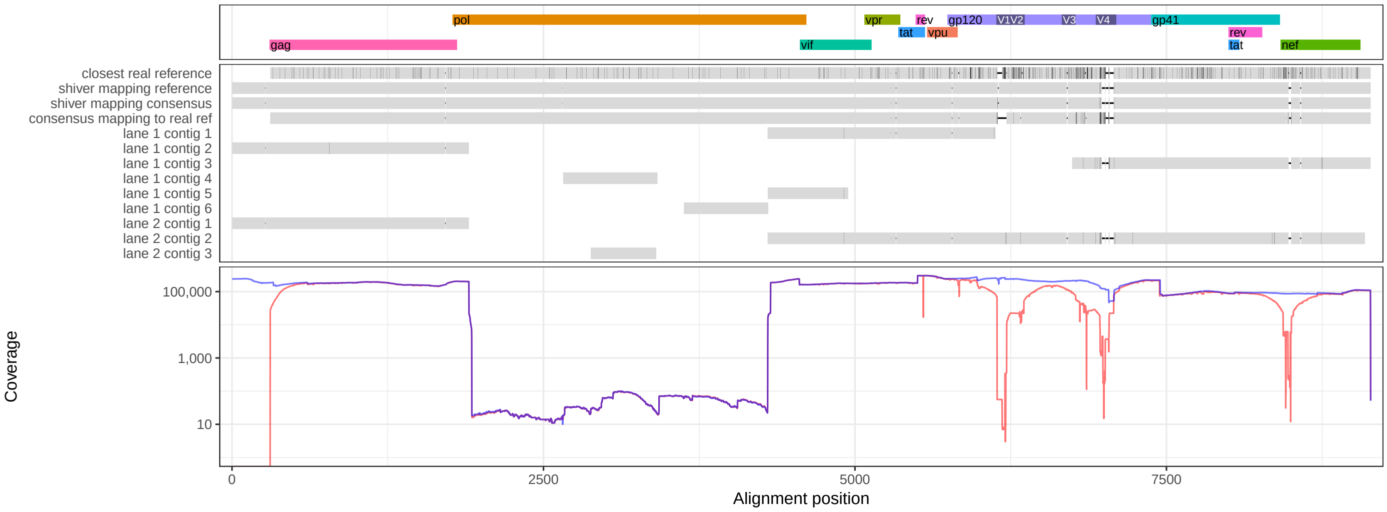

Figure S86: 17796_3_35 sequences and coverage (mapping to the `shiver` reference in blue, to the real reference in red).

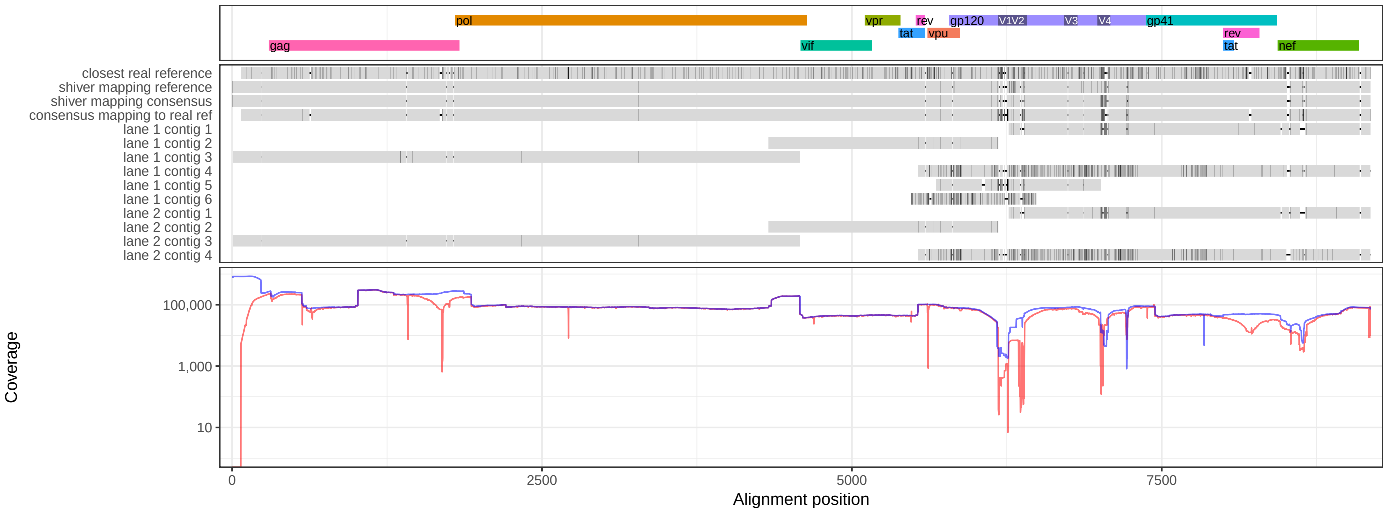

Figure S87: 18209_3_31 sequences and coverage (mapping to the `shiver` reference in blue, to the real reference in red).

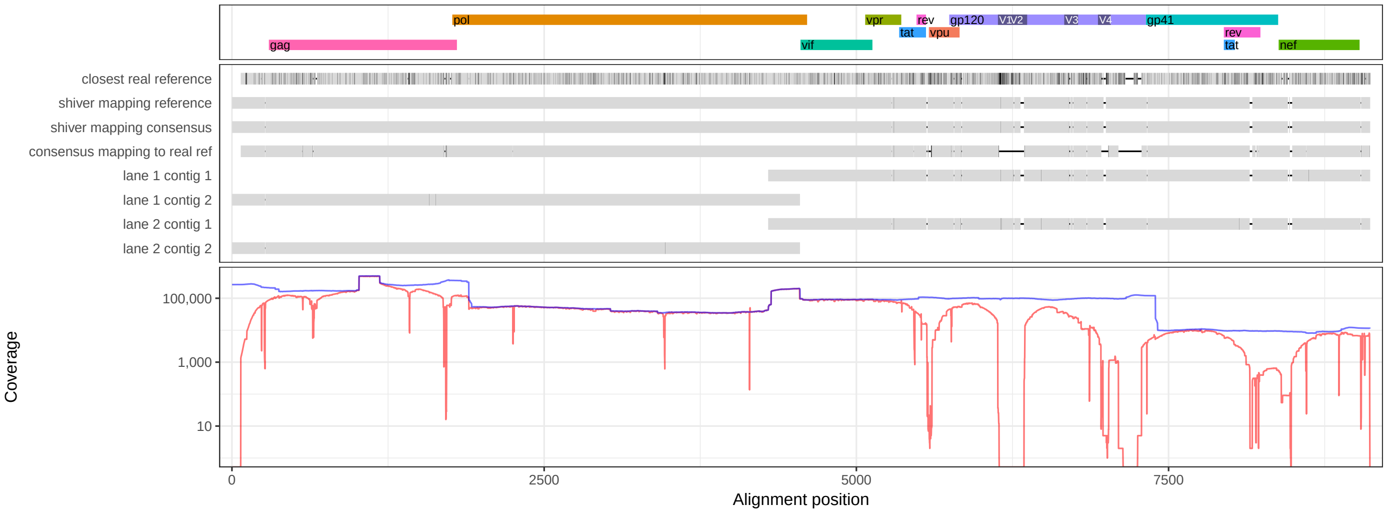

Figure S88: 18209_3_36 sequences and coverage (mapping to the `shiver` reference in blue, to the real reference in red).

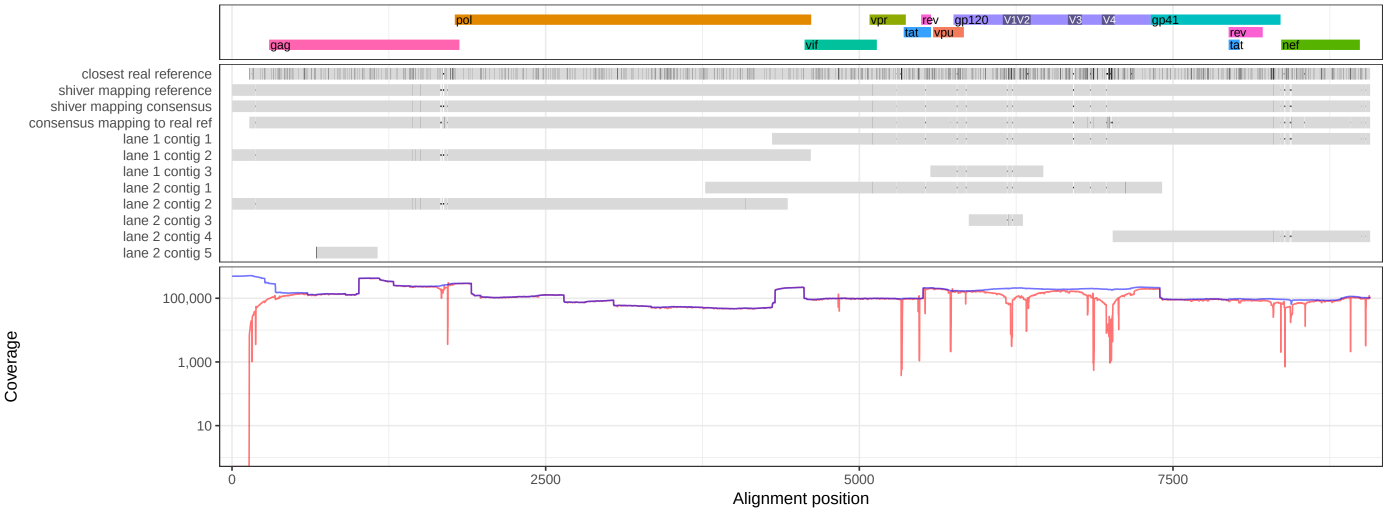

Figure S89: 18209_3_38 sequences and coverage (mapping to the `shiver` reference in blue, to the real reference in red).

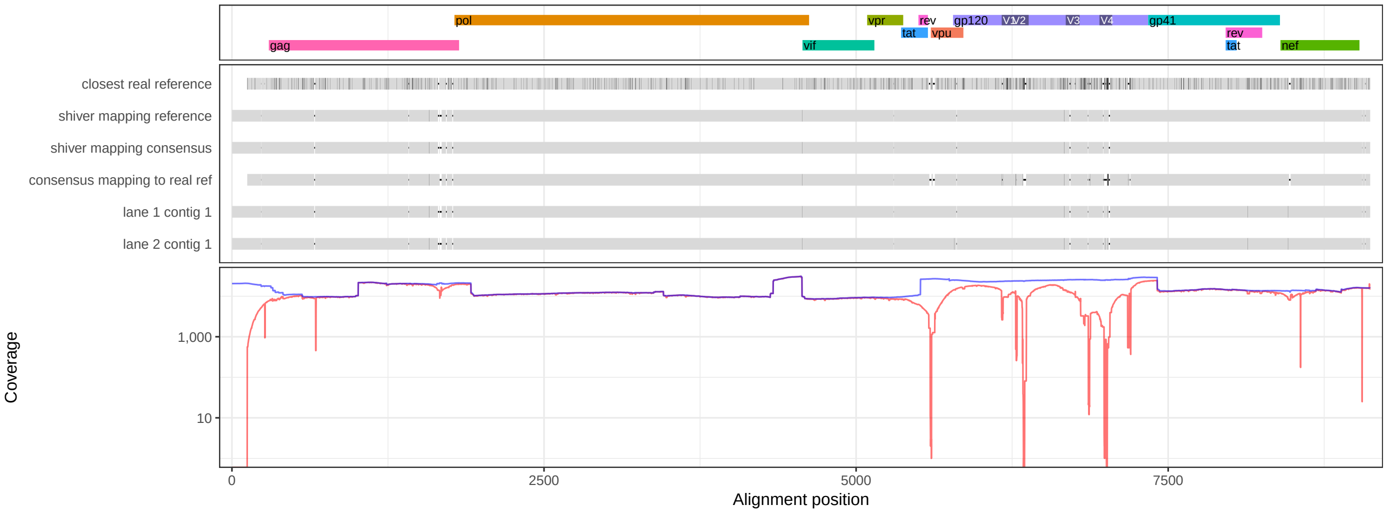

Figure S90: 19561_3_127 sequences and coverage (mapping to the `shiver` reference in blue, to the real reference in red).

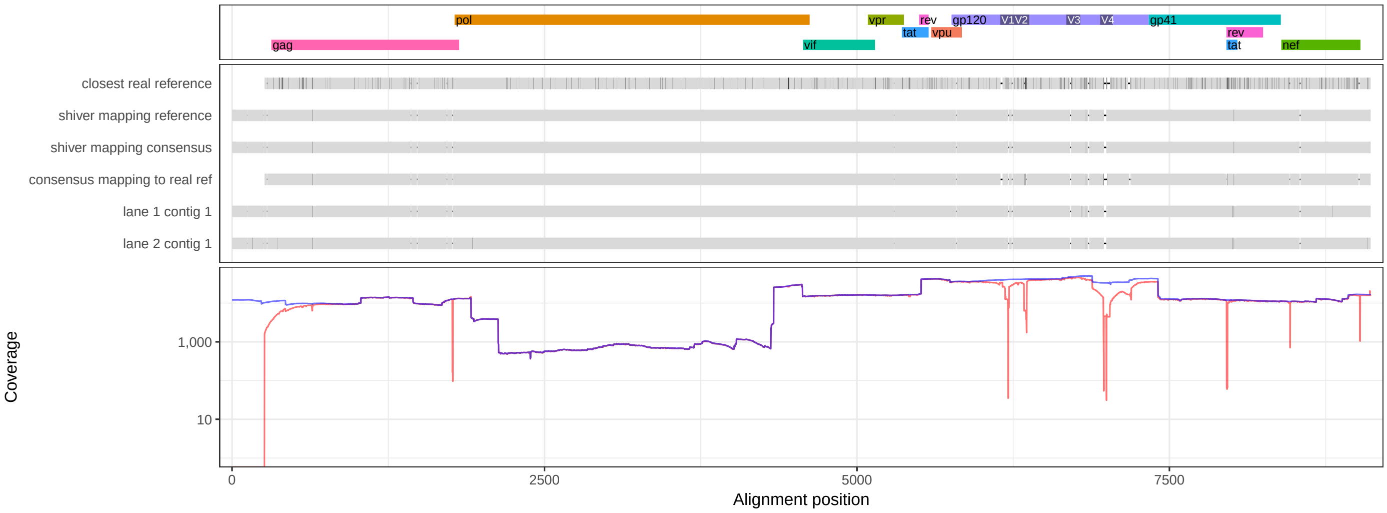

Figure S91: 19562_3_109 sequences and coverage (mapping to the shiver reference in blue, to the real reference in red).

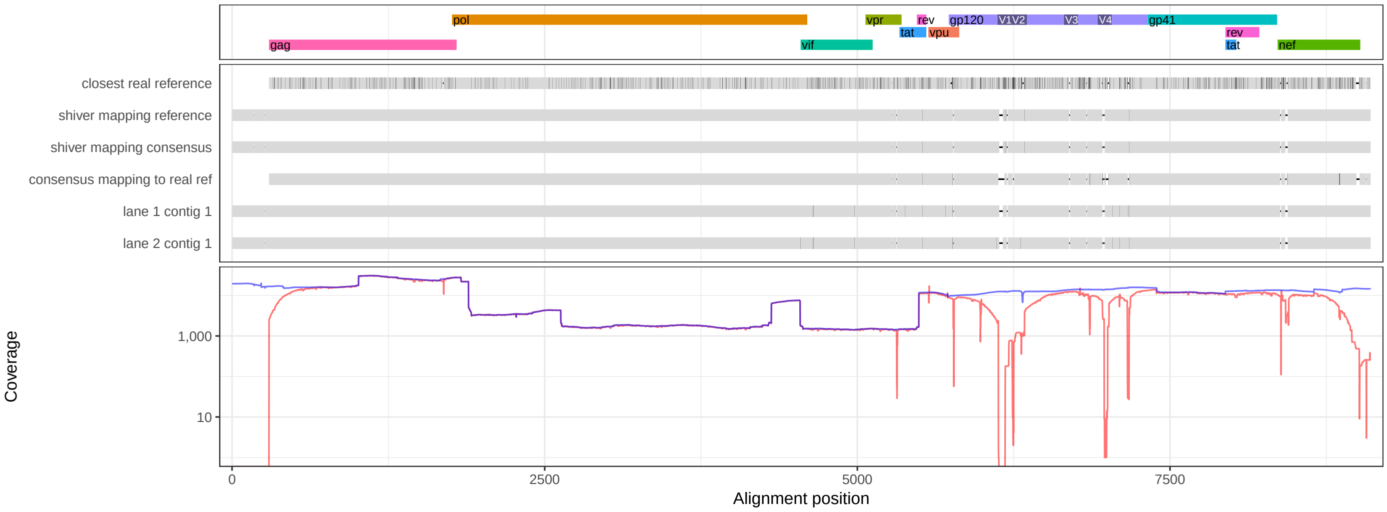

Figure S92: 19562_3_2 sequences and coverage (mapping to the shiver reference in blue, to the real reference in red).

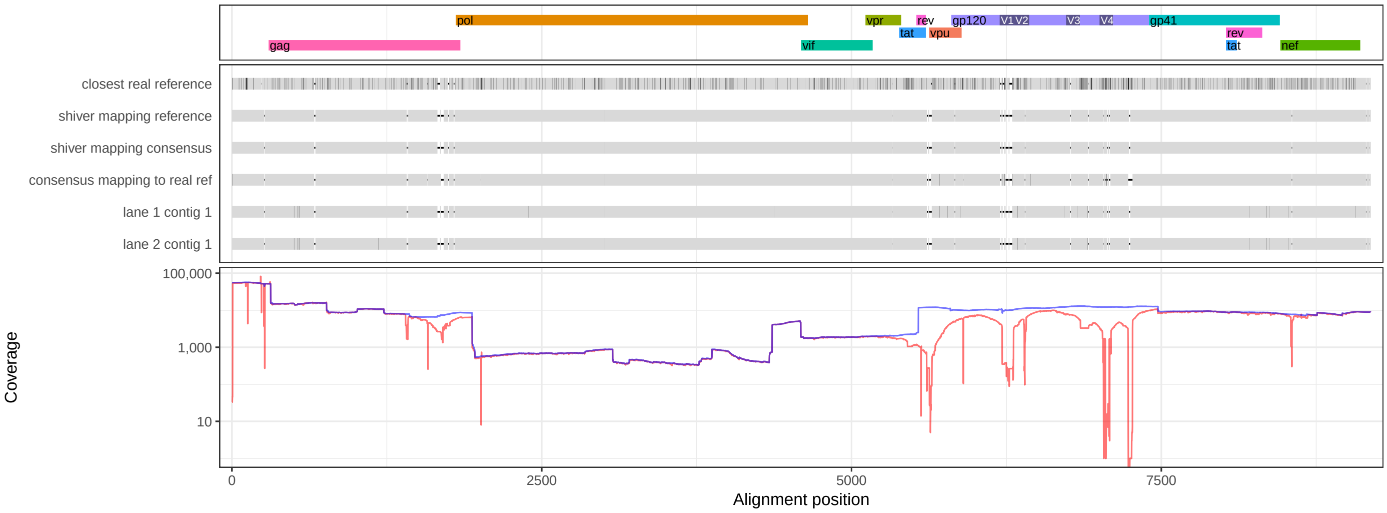

Figure S93: 19562_3_30 sequences and coverage (mapping to the shiver reference in blue, to the real reference in red).

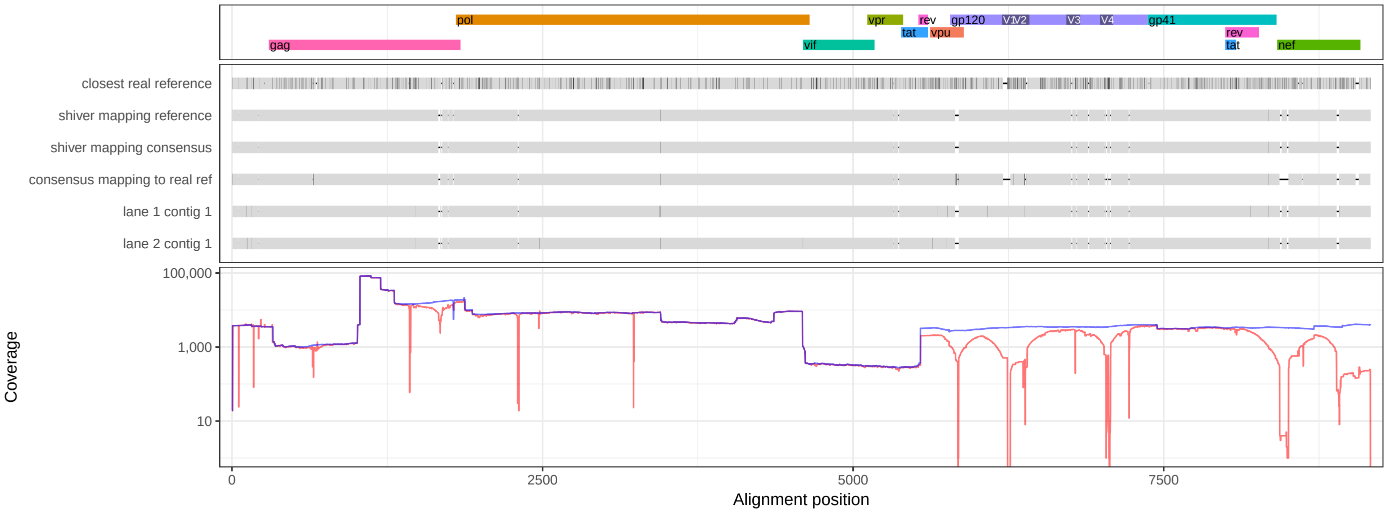

Figure S94: 19562_3_31 sequences and coverage (mapping to the shiver reference in blue, to the real reference in red).

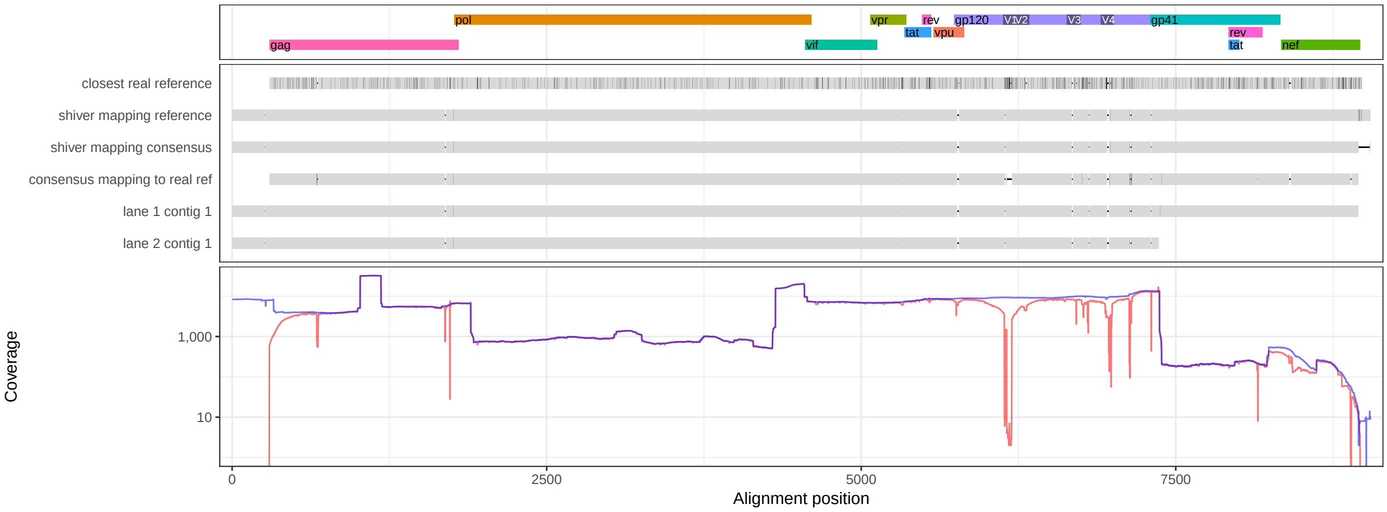

Figure S95: 19562_3_46 sequences and coverage (mapping to the shiver reference in blue, to the real reference in red).

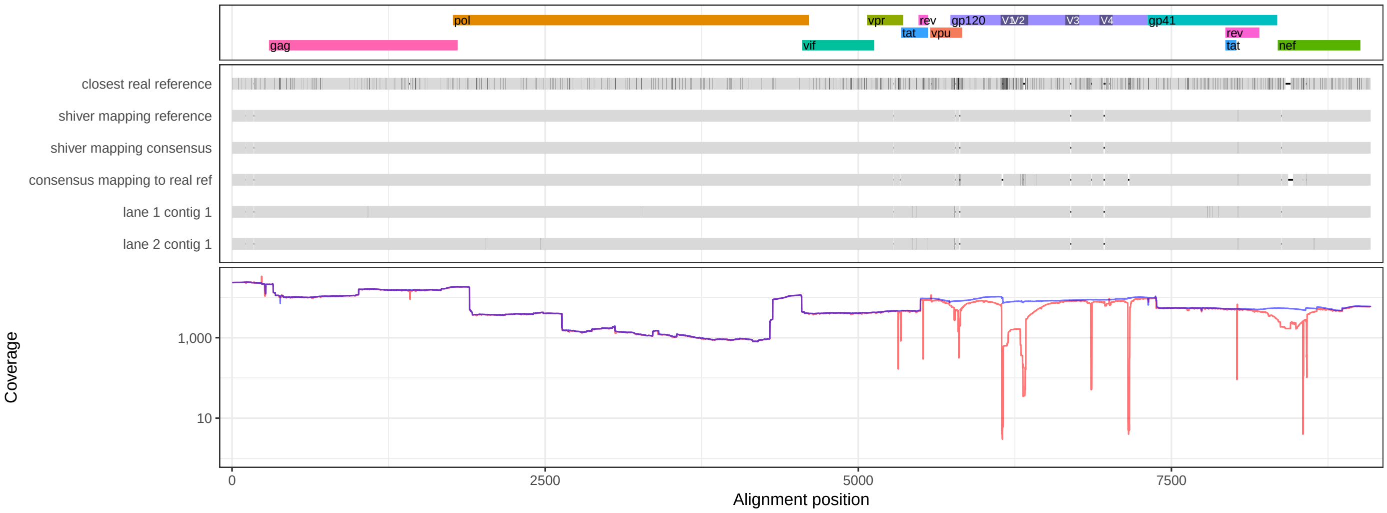

Figure S96: 19562_3_50 sequences and coverage (mapping to the shiver reference in blue, to the real reference in red).

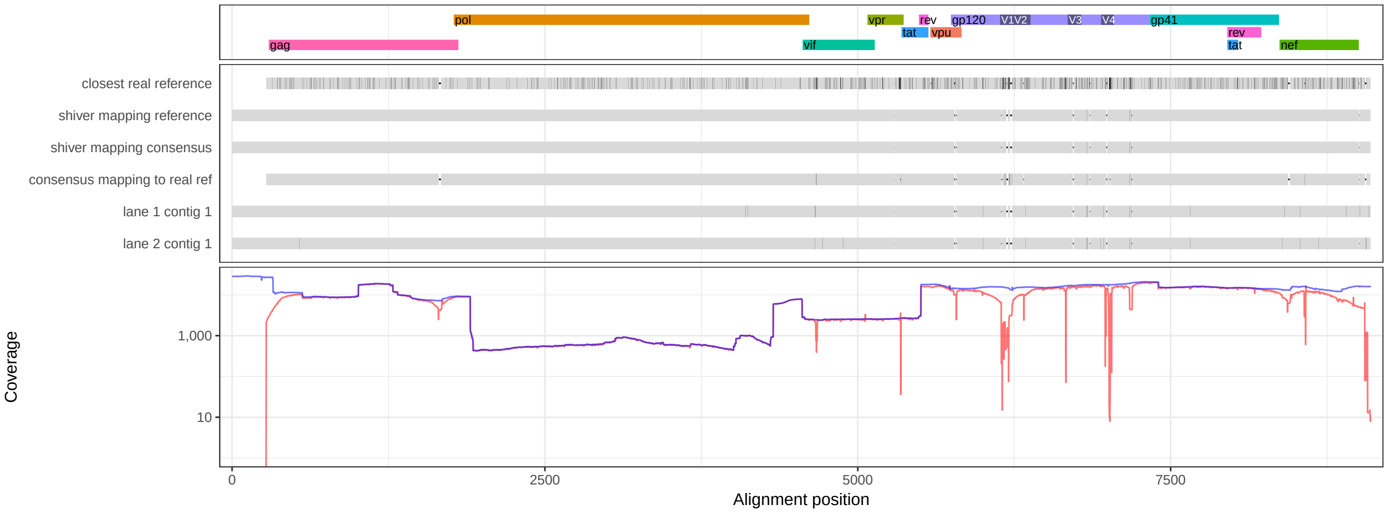

Figure S97: 19562_3_51 sequences and coverage (mapping to the `shiver` reference in blue, to the real reference in red).

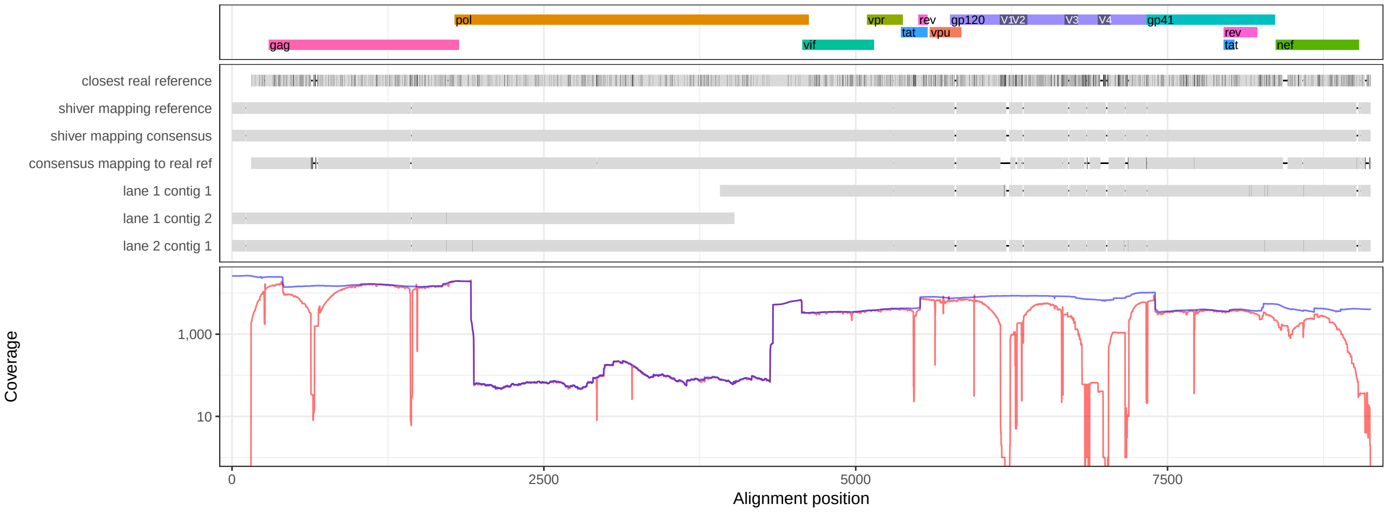

Figure S98: 19562_3_6 sequences and coverage (mapping to the `shiver` reference in blue, to the real reference in red).

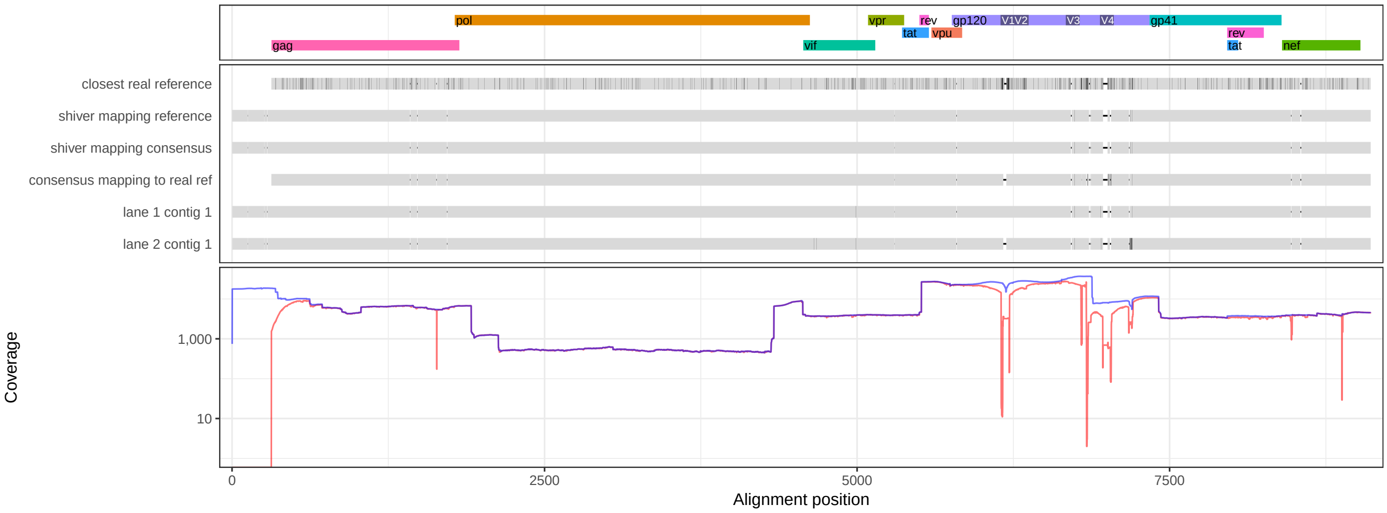

Figure S99: 19893_3_71 sequences and coverage (mapping to the `shiver` reference in blue, to the real reference in red).

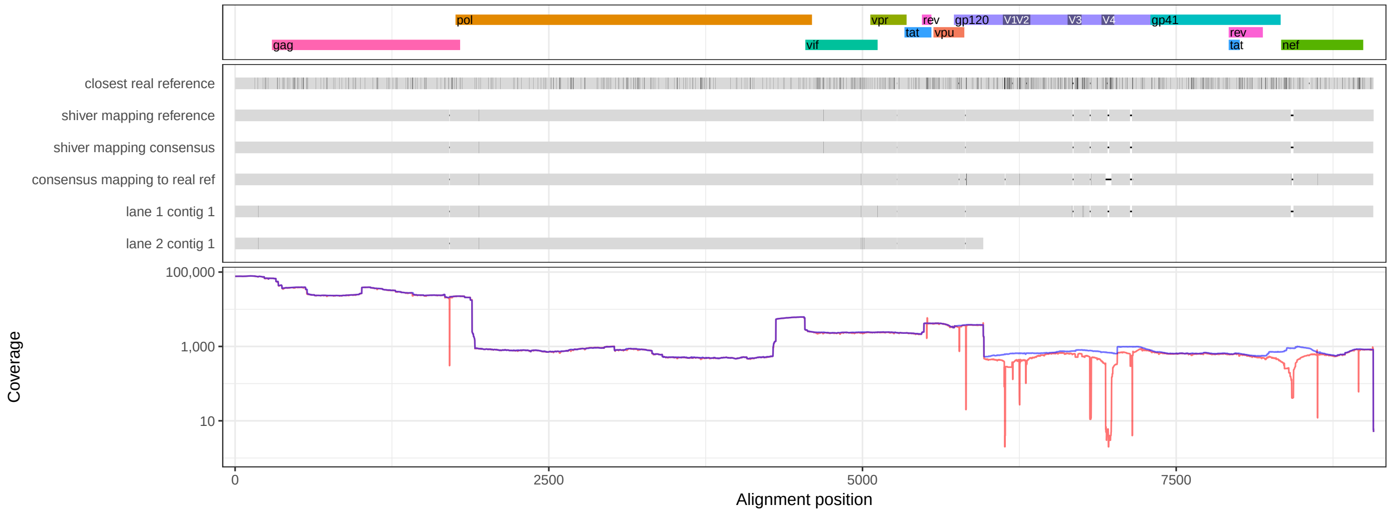

Figure S100: 19960_3_116 sequences and coverage (mapping to the `shiver` reference in blue, to the real reference in red).

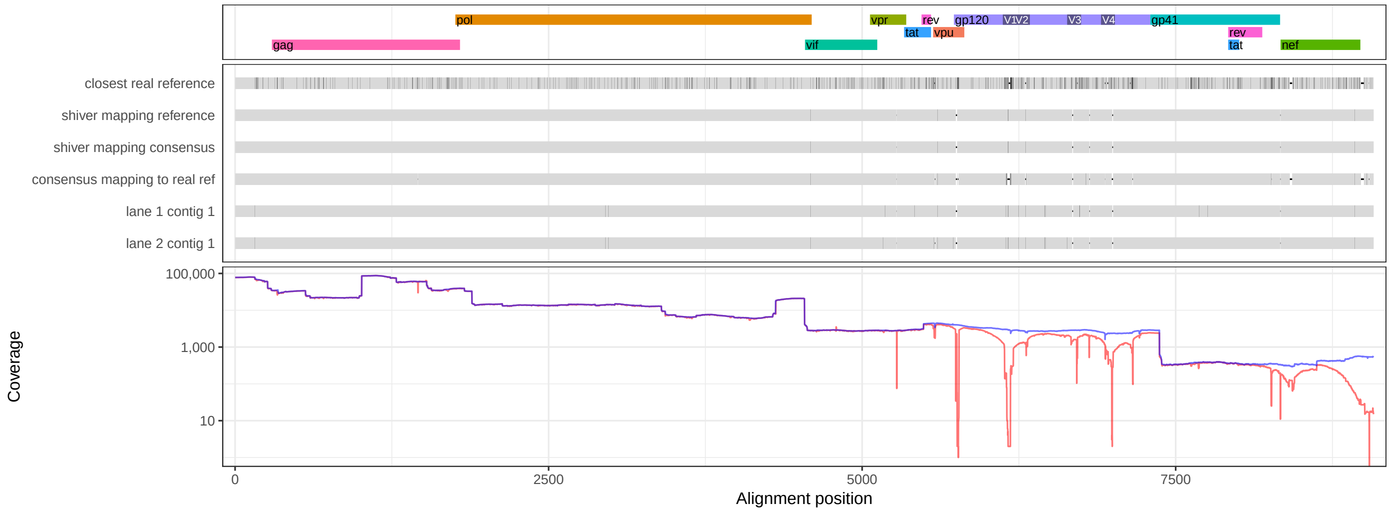

Figure S101: 19960_3_119 sequences and coverage (mapping to the `shiver` reference in blue, to the real reference in red).

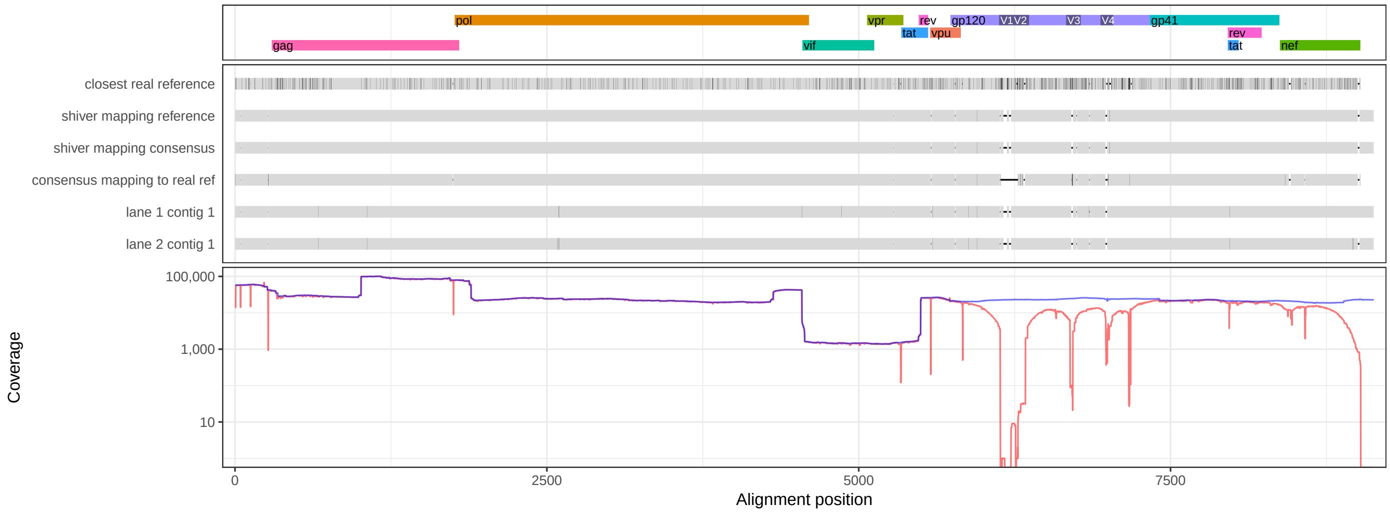

Figure S102: 19960_3_11 sequences and coverage (mapping to the `shiver` reference in blue, to the real reference in red).

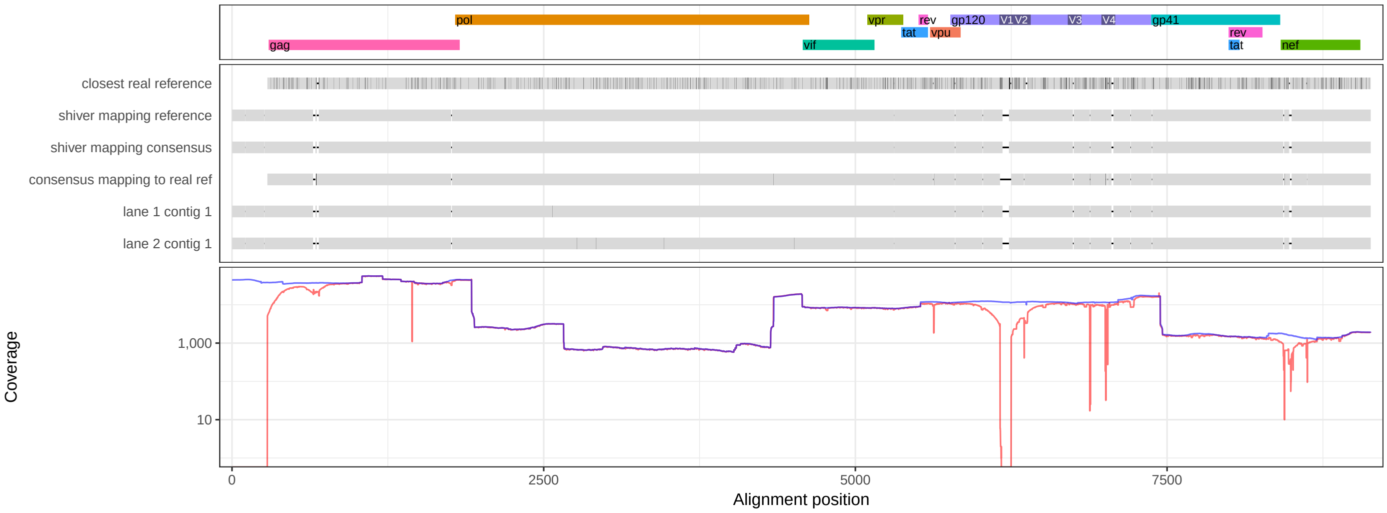

Figure S103: 19960_3_12 sequences and coverage (mapping to the `shiver` reference in blue, to the real reference in red).

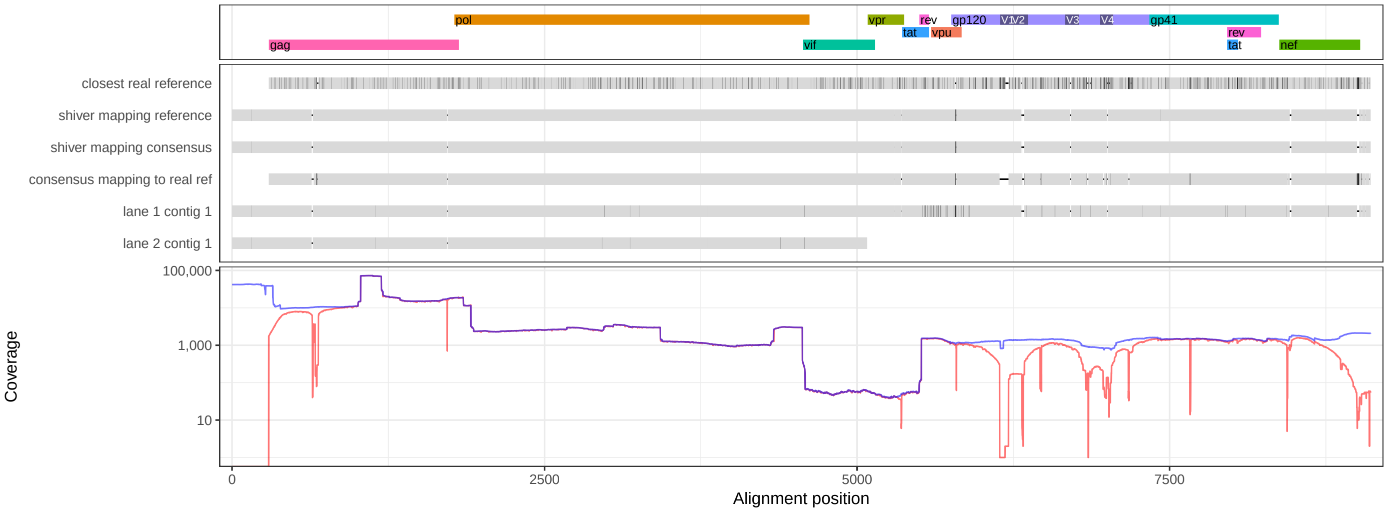

Figure S104: 19960_3_146 sequences and coverage (mapping to the `shiver` reference in blue, to the real reference in red).

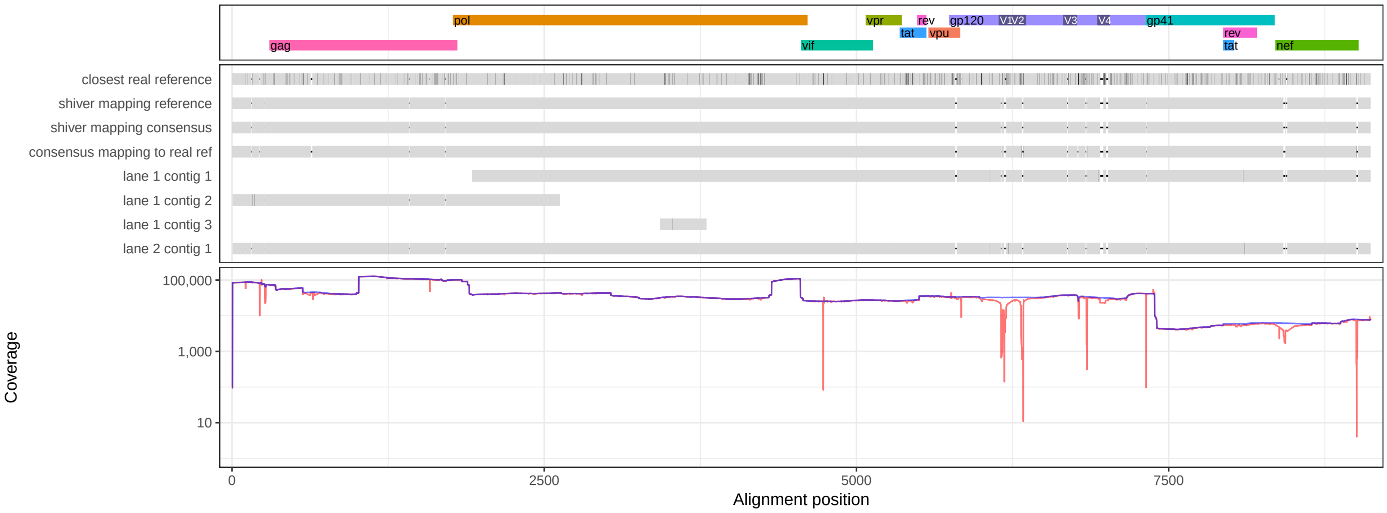

Figure S105: 19960_3_15 sequences and coverage (mapping to the `shiver` reference in blue, to the real reference in red).

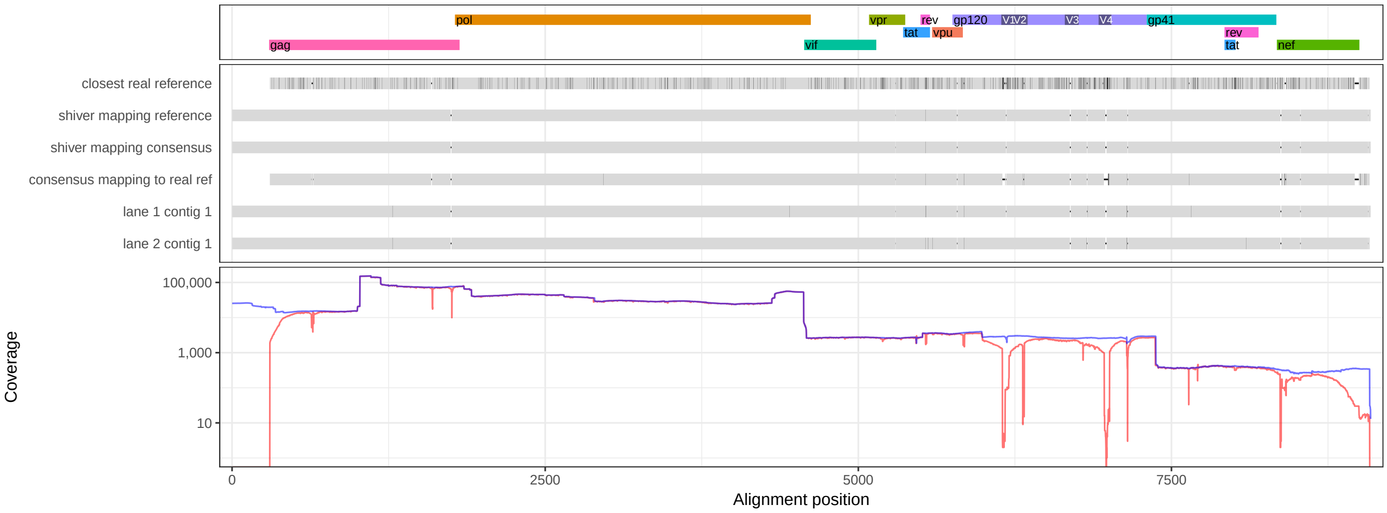

Figure S106: 19960_3_16 sequences and coverage (mapping to the `shiver` reference in blue, to the real reference in red).

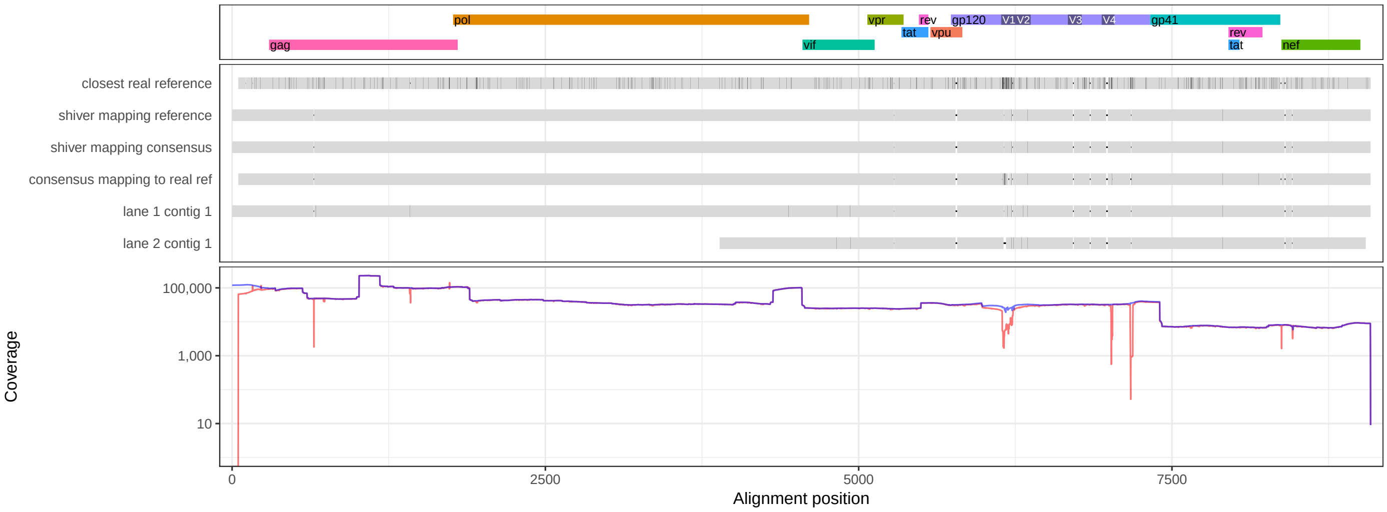

Figure S107: 19960_3_17 sequences and coverage (mapping to the `shiver` reference in blue, to the real reference in red).

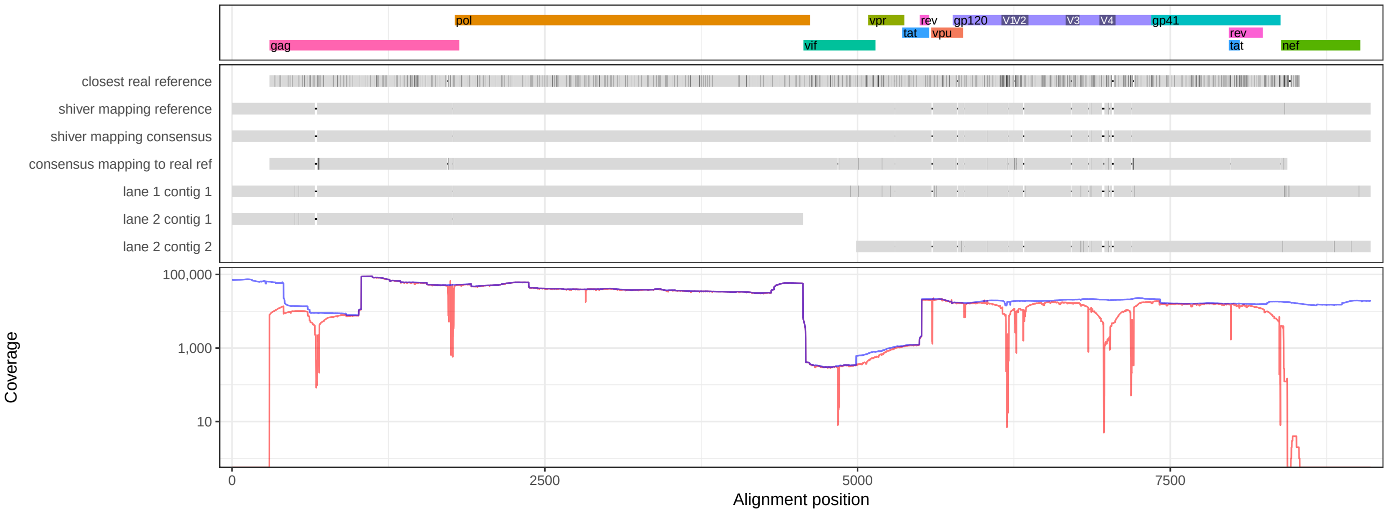

Figure S108: 19960_3_18 sequences and coverage (mapping to the `shiver` reference in blue, to the real reference in red).

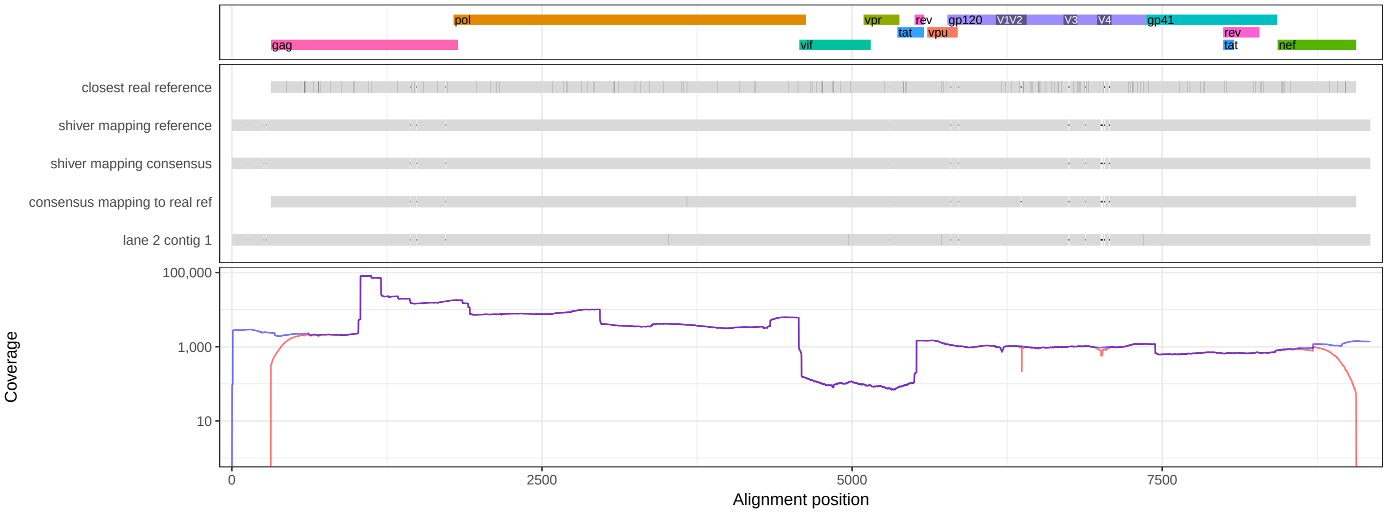

Figure S109: 19960_3_22 sequences and coverage (mapping to the shiver reference in blue, to the real reference in red).

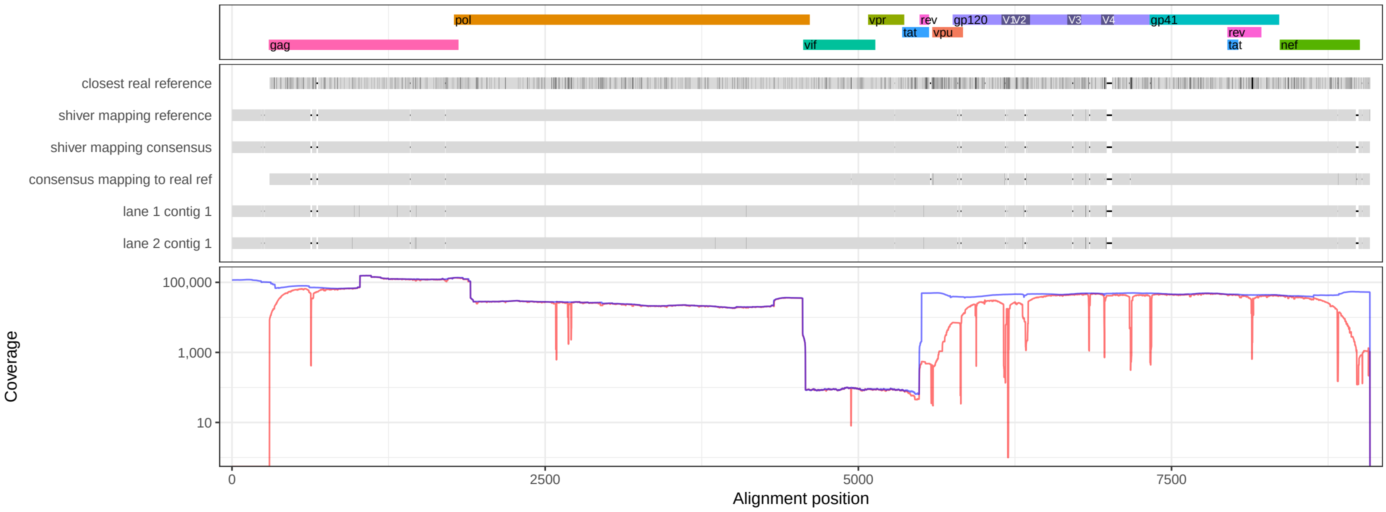

Figure S110: 19960_3_28 sequences and coverage (mapping to the shiver reference in blue, to the real reference in red).

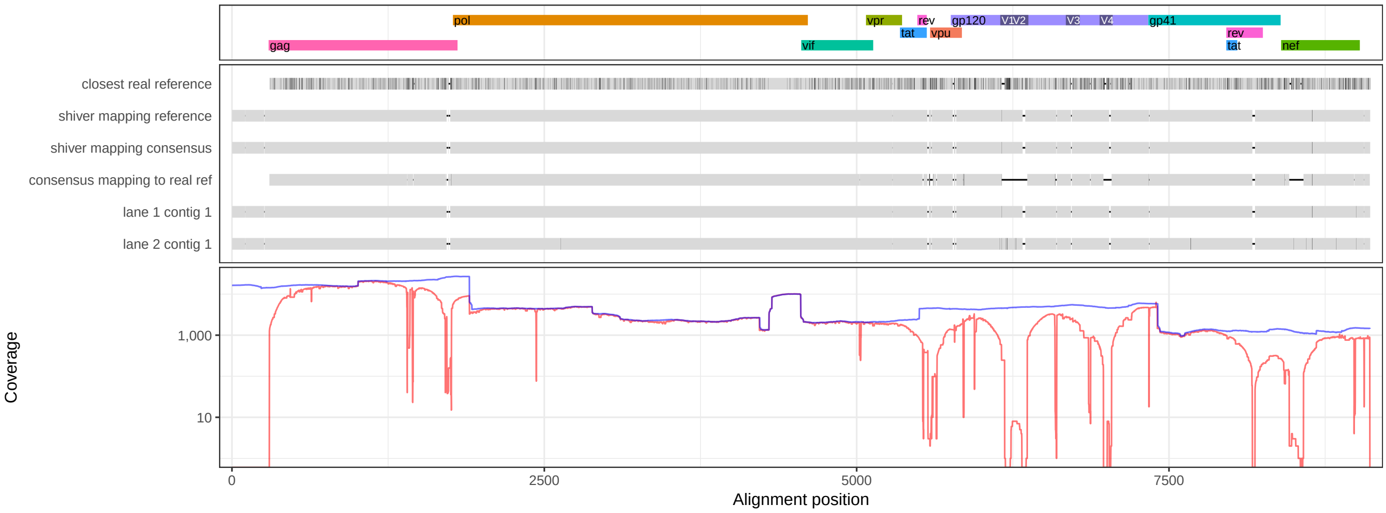

Figure S111: 19960_3_40 sequences and coverage (mapping to the shiver reference in blue, to the real reference in red).

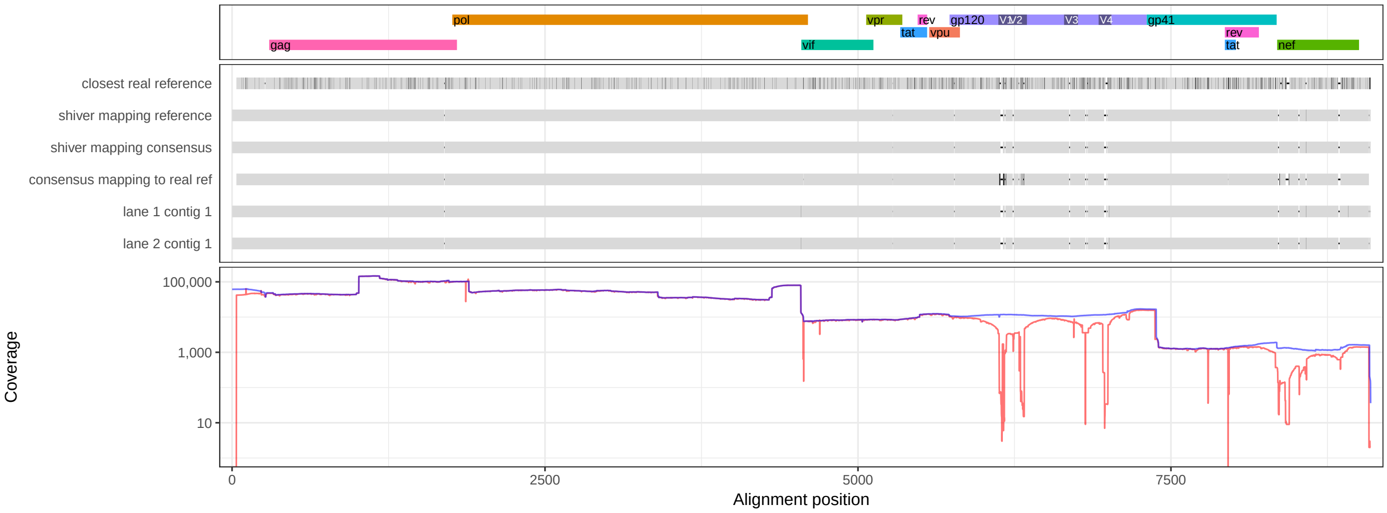

Figure S112: 19960_3_44 sequences and coverage (mapping to the `shiver` reference in blue, to the real reference in red).

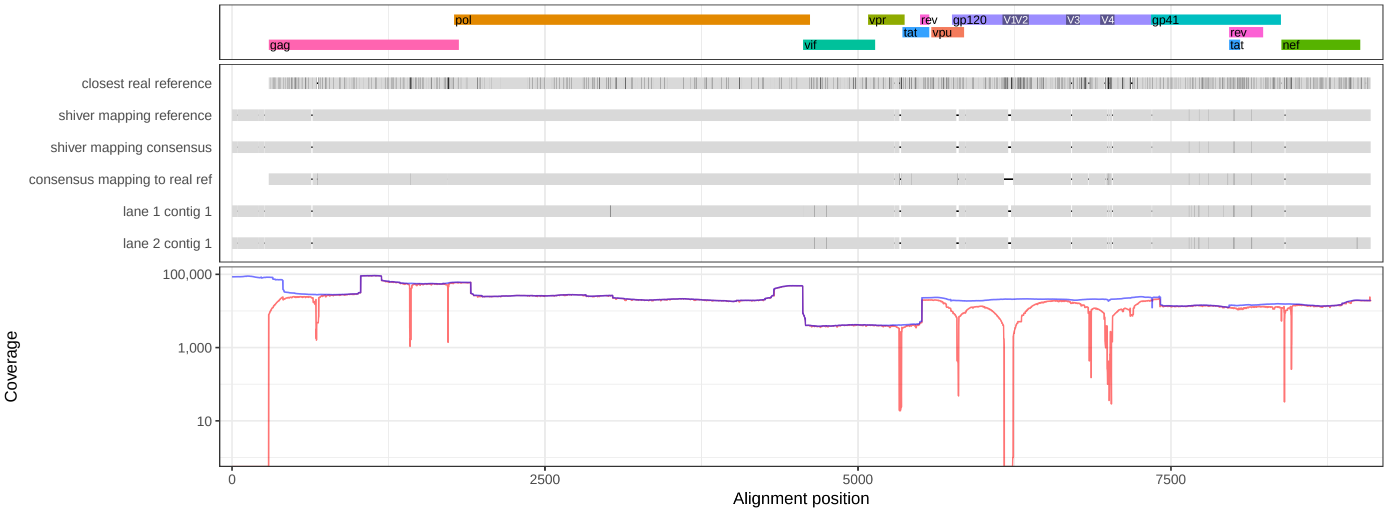

Figure S113: 19960_3_49 sequences and coverage (mapping to the `shiver` reference in blue, to the real reference in red).

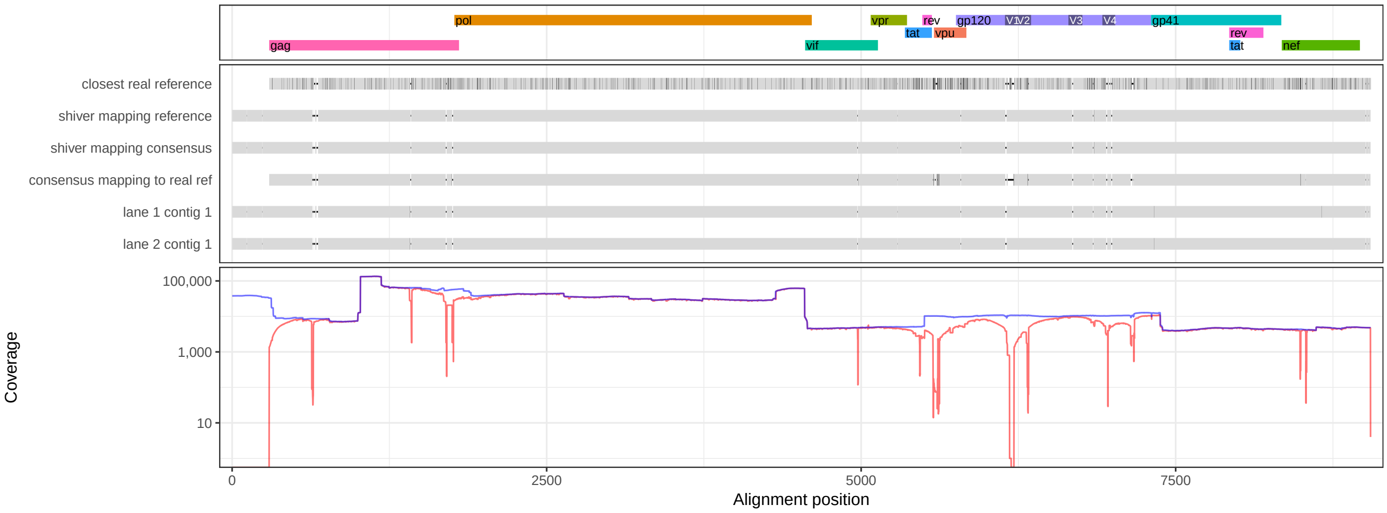

Figure S114: 19960_3_6 sequences and coverage (mapping to the `shiver` reference in blue, to the real reference in red).

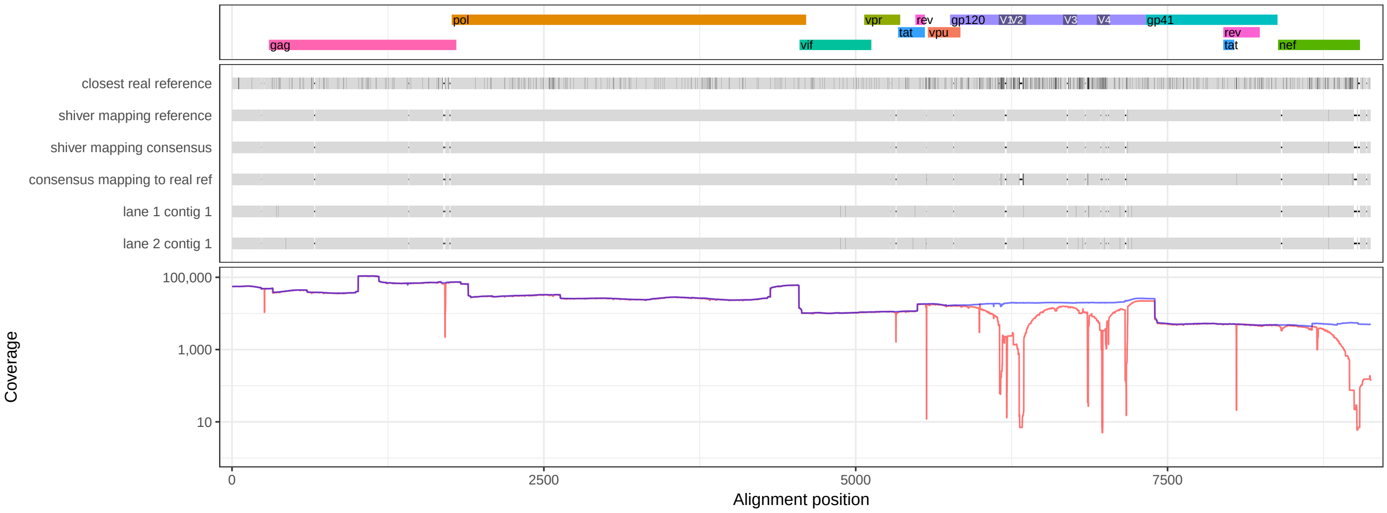

Figure S115: 19960_3_70 sequences and coverage (mapping to the `shiver` reference in blue, to the real reference in red).

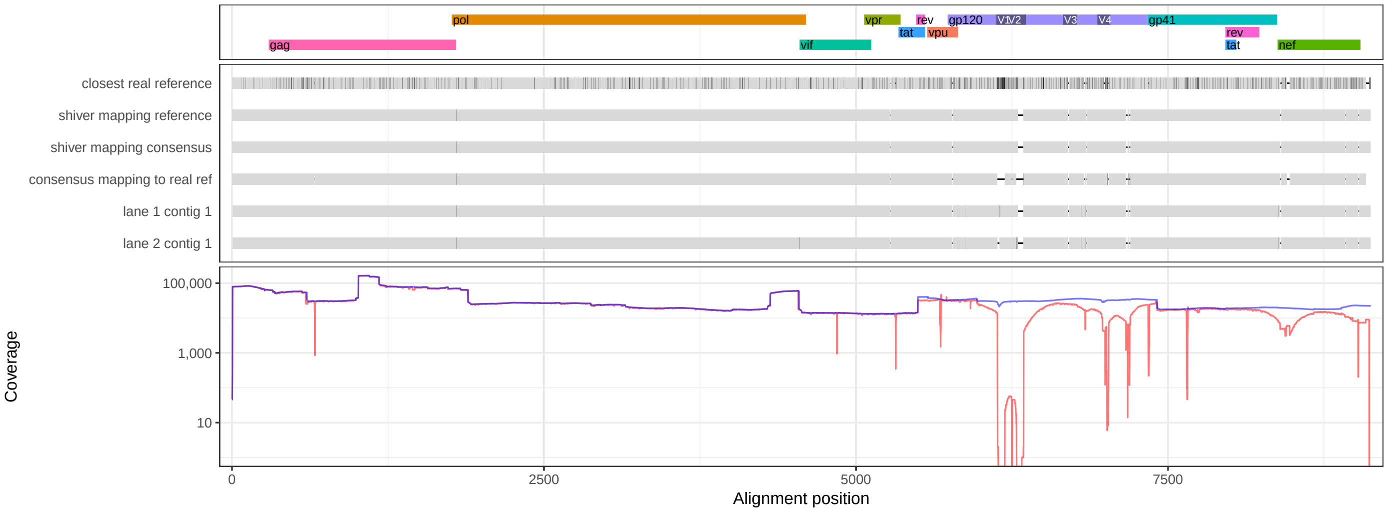

Figure S116: 19960_3_9 sequences and coverage (mapping to the `shiver` reference in blue, to the real reference in red).

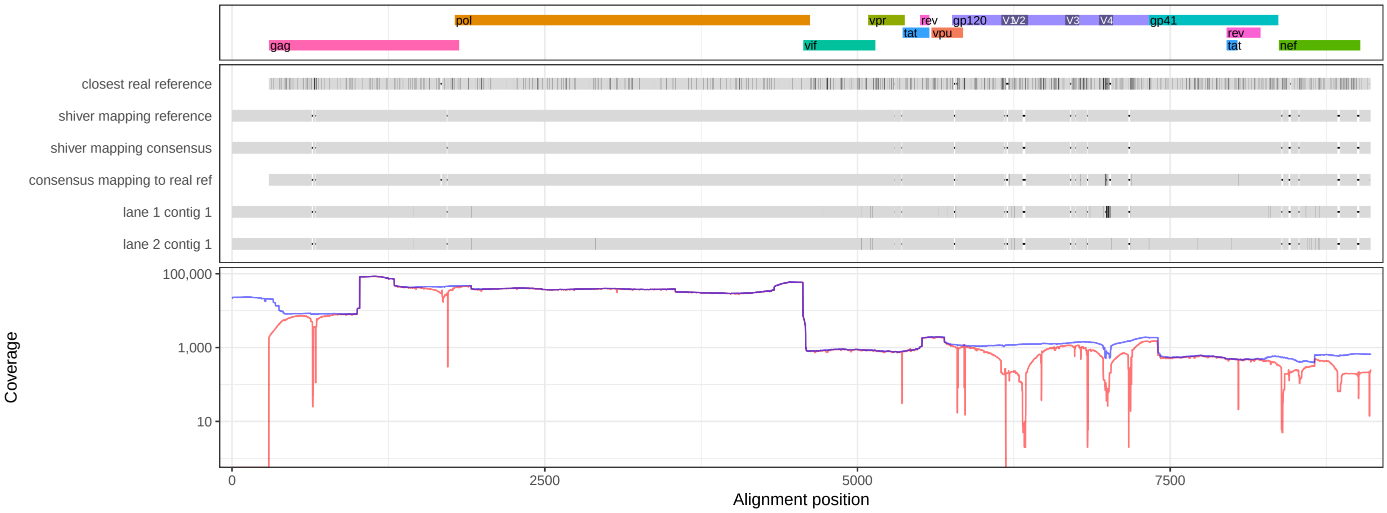

Figure S117: 20004_3_146 sequences and coverage (mapping to the `shiver` reference in blue, to the real reference in red).

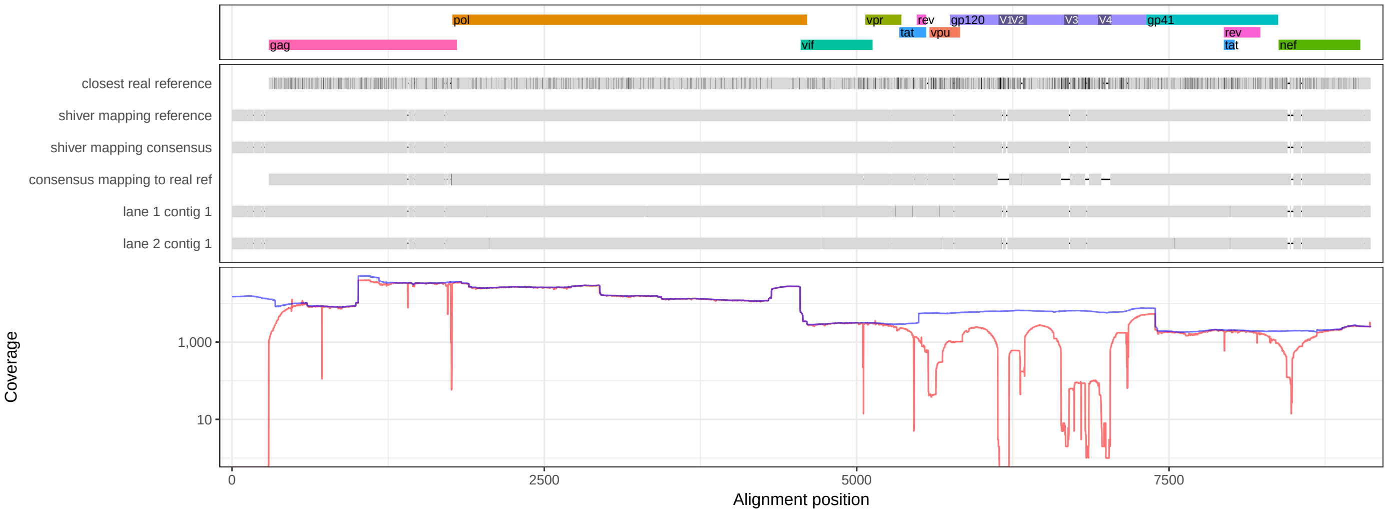

Figure S118: 20004_3_155 sequences and coverage (mapping to the `shiver` reference in blue, to the real reference in red).

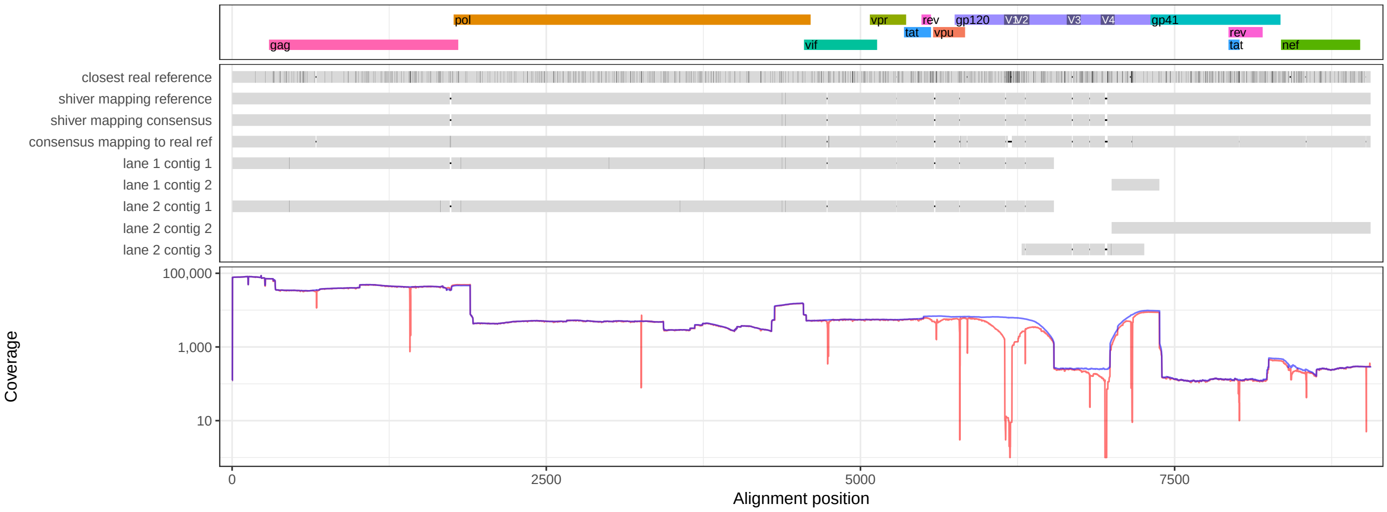

Figure S119: 20004_3_56 sequences and coverage (mapping to the `shiver` reference in blue, to the real reference in red).
