# Supplementary material for: Easy and accurate reconstruction of whole HIV genomes from short-read sequence data with shiver
Source: Virus Evol. 2018 May 18;4(1):vey007. doi: 10.1093/ve/vey007 (PMC5961307; doi:10.1093/ve/vey007)
Supplement: Supplementary Data [file vey007_supp.zip › PipelinePaper_v4_SIonly.pdf]

Supplementary Information for *Easy and Accurate Reconstruction of Whole HIV Genomes from Short-Read Sequence Data with SHIVER*

SI 1 Sequencing Platform Usage Statistics for HIV

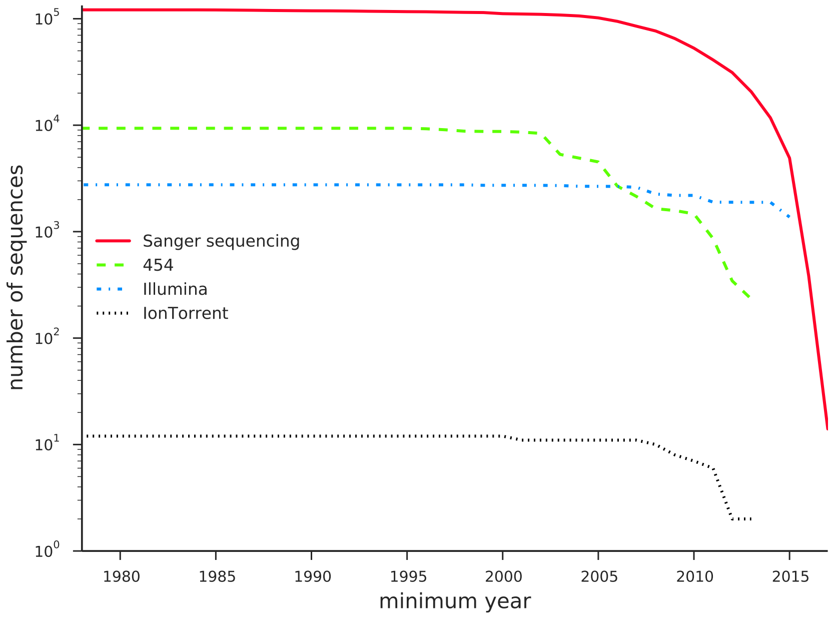

Figure S1: the number of HIV sequences available from the Los Alamos National Laboratory database on 11th Oct 2017 with sampling year and sequencing platform information available, as a function of minimum sampling year for inclusion (i.e. restricting the included sequences to increasingly recent ones).

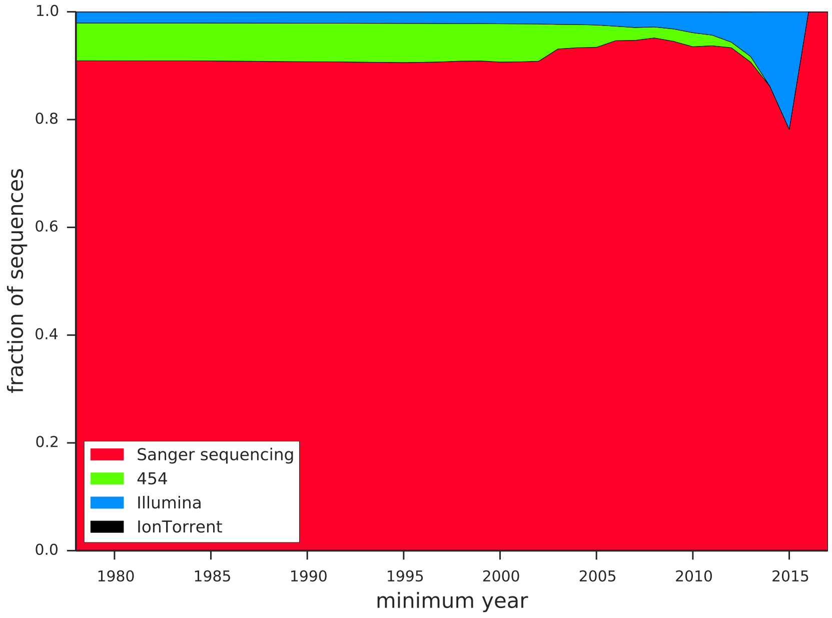

Figure S2: As Fig. S1 but showing the fraction for each platform.

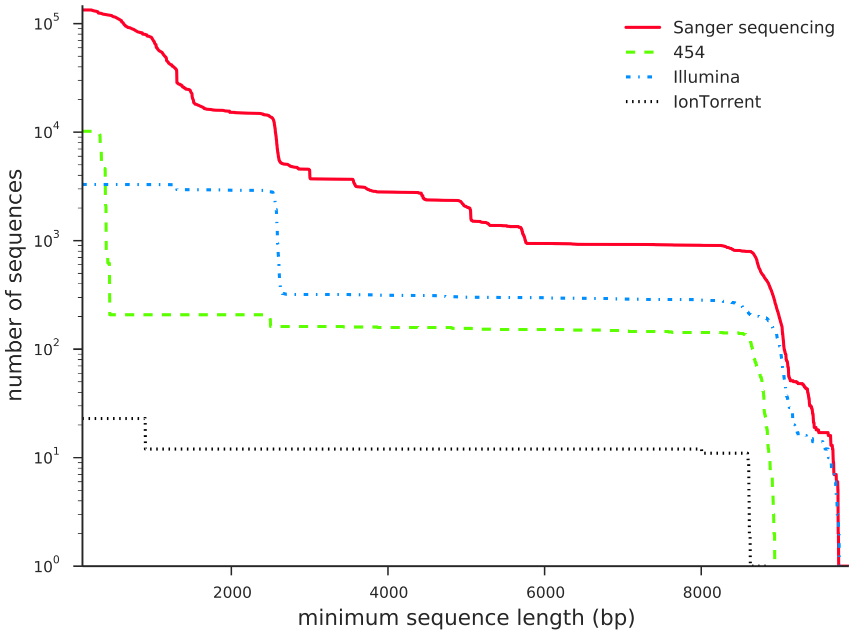

Figure S3: the number of HIV sequences available from the Los Alamos National Laboratory database on 11th Oct 2017 with sequencing platform information available, as a function of minimum sequence length for inclusion (i.e. restricting the included sequences to increasingly long ones).

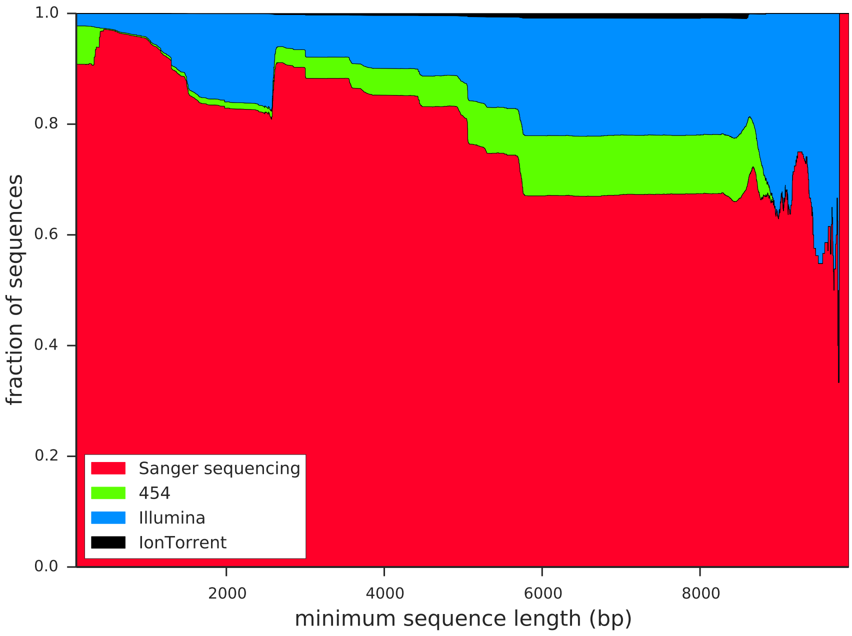

Figure S4: As Fig. S3 but showing the fraction for each platform.

SI 2 Our Method in More Detail

shiver is under continuing development; if at a later date description here contradicts descriptions at github.com/ChrisHIV/shiver, the latter has precedence.

2.1 Existing References

An alignment of existing reference sequences is required as input for shiver. Construction of a custom reference for mapping involves identifying the existing references that are closest to the sample under consideration. The greater the number and diversity of existing references given as input, the denser and broader the coverage of sequence space is, and the closer the closest reference is expected to be, with corresponding benefits for the accuracy of the results. However these existing references should be aligned to each other accurately, in order for the addition of each sample's contigs to the alignment to be meaningful; this means that producing such an input by automatically aligning a large number of diverse sequences without checking the results would be a bad idea. You will use this alignment as input for every sample in a dataset processed by shiver, and so we advise putting a little thought into sequence selection and manually curating the alignment if needed.

2.2 Constructing a Tailored Reference Using the Contigs

Custom reference construction begins with contig preprocessing as follows. Matches between the contigs and any existing reference from the alignment are searched for using BLASTN with default settings, except for the -max_target_seqs 1 option (specifying that all reported hits are to a single reference only), and with -word_size set to 17 (this can be changed in shiver's configuration file). Contaminant sequence is inevitable in high-throughput NGS; any contig that has no BLASTN hit to any of the HIV references is taken to be contamination, and is put aside for later use, leaving contigs that are putatively HIV. The BLASTN results are used to correct the contigs in three ways.

1. Where a single contig has multiple BLASTN hits (discarding any hit wholly contained inside another hit), we consider this evidence that the contig is spliced – concatenating two separated regions of the genome – due to errors *in silico* or during sequencing, as mentioned in the introduction. We correct this by cutting the contig into separate contigs at the midpoint between the hits.

2. We trim off any part of the contig that was not spanned by a BLASTN hit. The ends of contigs are by definition points at which the assembler has been unable to continue extending the sequence, either because of lack of reads, or because the within-sample diversity has become too great for a single, meaningful, representative sequence to be chosen. The latter possibility also means erroneous bases are more common in short stretches of sequence at the end of a contig. Trimming such sequence from the ends of contigs means the corresponding sequence from the closest existing reference will be used instead, giving a better reference for mapping. (Some assembly algorithms trim a fixed length from the ends of contigs for precisely this reason; however trimming a variable length dependent on its match to known sequence is clearly preferable.)

3. Any contig whose BLASTN hit is in the opposite orientation is reverse-complemented. If the assembler does not orientate the contigs, on average half of them will be in the reverse orientation. IVA orientates contigs such that the longest open reading frame is on the forward strand, however for very short contigs this may fail. In the process of assembling a spliced contig, an assembler may concatenate different regions in different orientations; shiver considers whether each separate part of a split contig requires reverse-complementation.

Contigs are then aligned to the existing reference alignment using MAFFT, trying both --add and --addfragments modes and using the one with the smallest maximum gap fraction (the maximum calculated over all contigs in each alignment). After alignment, a contig found to have an overly large internal deletion (by default 160bp) is split into two separate contigs at that point. This has the same role as BLASTN-based correction step 1 above, serving as a backup.

The alignment of contigs to the set of existing references should be visually inspected at this point. For HIV sequences, reference [1] states that "Algorithmic alignment does not necessarily retrieve the best alignment. It is important to always verify whether the sequence data are aligned unambiguously and, if necessary, manually correct the alignment." Reference [2] echoes this for any evolving pathogen: "the

'best' alignment chosen by an alignment program is not necessarily the 'true' alignment... Alignment quality should also be inspected manually in a visualisation program". The commonness of indels in HIV makes alignment more difficult, as does the fact that the contigs may be an imperfect representation of the true sample even after correction. We used `Geneious` [3] for sequence visualisation and editing where needed.

As well as revealing alignment error, inspection of the aligned contigs allows the user to check for any remaining problems with the contigs. We suggest that in general the user inspects both the alignment of the existing references with the *raw* HIV contigs (before any correction by shiver), and the alignment of the existing references with the *corrected* HIV contigs, as a check that all `shiver`'s modifications of the contigs are desired. An example of when this might not be the case is when the sample contains an indel not observed in the existing reference set, that is large enough to cause the contig to be split in two at that point, but which the user thinks might be genuine rather than an a misassembly (through previous/expert knowledge, or perhaps simply observing the same indel in multiple samples in a dataset). With sufficiently accurate mapping, reads will map here correctly whether or not the reference constructed from the contigs contains the indel, making the question moot; however with mapping inaccuracies of the kind shown in Figure 2 possible, it's best to get the reference's structure as correct as possible before mapping.

Using the alignment of contigs to existing references, the set of contigs is flattened into a single sequence as follows. At positions covered by one contig, its base (or gap character, for a deletion) is used. At positions covered by multiple contigs, we use whatever the longest contig has (be it base or gap). We used this heuristic expecting that, where sufficiently distinct haplotypes exist to result in multiple contigs covering the same place, haplotypes supported by a higher depth of reads would tend to be assembled into longer contigs. The sequence resulting from this flattening of the contigs is compared to each existing reference in the alignment in turn: we count towards similarity shared bases and gaps within contigs (known deletions), but not gaps between contigs (missing information). The existing references are ranked by their similarity to the contigs. As existing references have variable lengths (the long terminal repeat regions that flank the clinical genome are sometimes sequenced only partially or not at all), the closest reference is extended outwards using any overhanging sequence from the second closest reference, then the third longest sequence etc. terminating when both edges of the alignment are reached. This sequence – the elongated closest reference – is used to fill in any gaps between (but not inside of) the flattened contigs. This completes production of the reference tailored for this sample.

2.3 Preparing and Mapping the Reads

Before mapping to this reference, the reads are trimmed and cleaned as follows. Adapters, primers and low quality bases are trimmed using `Trimmomatic` and `Fastaq`. We then consider contaminant reads from non-HIV sources. Most of these would presumably be discarded by mapping to an HIV reference, due to lack of similarity. However there is ample opportunity for traces of human DNA to end up in a sample, and sequence of endogenous retroviruses in human DNA may resemble HIV. As a guard against this, and against any other contamination resembling HIV, we use `BLASTN` to find all read pairs that are a better match to one of the contigs previously found to be contamination, than to the tailored reference. These pairs are discarded.

The cleaned reads are mapped to the tailored reference, using `SMALT` by default (with `BWA` and `bowtie` as optional alternatives), giving a file in BAM format. Using `SAMtools` the BAM file is read into pileup format, which is parsed to give base frequencies at each position in the genome. Note that within-host diversity does not consist exclusively of point mutations: indels can be present in some reads and not others (Fig. 7 is an example), which must be dealt with in the pileup. Where some reads have a deletion relative to the reference and others do not, the deletion/gap character can simply be considered as a fifth base whose frequency can be counted like the others. Where some reads have an insertion relative to the reference and others do not, or more generally where insertions of two or more sizes are present, we find the most common insertion size and, inside that insertion, consider only those reads with an insertion of that size (thus avoiding any ambiguity in the alignment of the inserted sequences to each other). Finally, the base frequency file is parsed to call the consensus base at each position. By default the most common base is called to give the consensus, using an ambiguity code only for an exact tie in the frequency of two or more bases; optionally ambiguity codes can be used more readily, when the frequency of the most common base or bases is below a specified threshold. A consensus base is only called if the coverage equals or exceeds a minimum threshold specified by the user, to protect against the effect of residual

low-coverage contaminant reads in genomic regions lacking genuine HIV reads. By default this is 15, but this is likely to need adjusting for different datasets. (See the tool `LinkIdentityToCoverage.py` in section 3.)

2.4 Aligning Multiple Consensuses

Since we know how the consensus aligns to the reference used for mapping, and we know how that reference (constructed from the contigs) aligns to the input alignment of existing references, we can construct a global alignment of the consensuses from all samples merely by coordinate translation, negating the need for further alignment and manual curation. Two things must be excised from the consensus for this global alignment reconstruction: insertions present in the majority of reads but not in their tailored reference (which are rare, since the reference is constructed from the contigs which are constructed from the reads), and insertions present in the contigs but none of the existing references (which are rare provided the set of existing references is large and diverse). In both cases this is sequence whose alignment to the common anchor of the existing references is not known, and so coordinate translation cannot align it.

2.5 Fully Automatic `shiver`

As mentioned, `shiver` can be run from beginning to end without the break in the middle, with the single command `shiver_full_auto.sh`, for uses where visually checking the contigs is impractical. This begins with separation of contigs into HIV (those with `BLASTN` hits) and contamination as previously. Subsequent steps are as follows.

1. The need for contig correction is checked, but correction is not performed: if it is needed, processing stops. Blind trust in the accuracy of an automated alignment of contigs cut into pieces based on evidence of structural problems would be trust misplaced.

2. Each HIV contig is now certain to have a single `BLASTN` hit (discarding any smaller hits wholly contained inside others). That hit is checked to span some minimum fraction of the contig length (by default 90%) as a guard against contigs containing containing some erroneous or foreign sequence; otherwise processing stops.

3. Multiple sequence alignment is performed with these contigs and just one of the existing reference sequences, for each of the existing reference sequences separately.

4. For each such alignment, generated both with regular `mafft` and with `mafft --addfragments`, we calculate the fractional agreement between the flattened contigs and the reference, i.e. the fraction of positions spanned by the reference and at least one contig where the reference and the longest contig agree. Misalignment is penalised in this score because gaps inside contigs are taken as genuine deletions.

5. For the alignment with the highest score, the maximum gap fraction amongst the contigs in the alignment (i.e. the fraction of positions inside the contig that are gaps) is checked to be below a user-specified threshold (the default is 5%, based on analysis of thousands of such alignments that we visually checked) as a further guard against misalignment.

6. The contigs are flattened using this single existing reference to fill in any gaps between them, generating the mapping reference tailored for this sample.

Aligning contigs to the references one at a time (step 3) is simpler for the alignment algorithm than aligning to all of them at once, and means that even if misalignment occurs for what is truly the closest reference to the contigs, the alignment to the second closest can be used instead. Trimming of low-quality bases, trimming of adapter and primer sequences, removal of contaminant reads and mapping to the tailored reference all occur as described previously. For samples that cannot be processed fully automatically this way – when contig correction is required, or a contig is spanned by too small a `BLASTN` hit, or too many gaps are present after alignment – the main mode of shiver is available (for which we advise inspection of the aligned contigs).

As argued earlier, we advocate visually inspecting the aligned contigs, i.e. running the two-command implementation of `shiver` (with the check occurring between the commands). This also has the advantage of working for all samples, whereas `shiver_full_auto.sh` will not proceed if problems with the contigs

or their alignment are detected. `shiver_full_auto.sh` also does not produce a global alignment of all consensuses to each other, because the coordinate translation procedure allowing its construction is derived from each sample's alignment of contigs to all of the references at once. That alignment is produced for the two-command implementation of `shiver`, but step 3 above aligns contigs to references one at a time.

SI 3 Sample Reprocessing and Analysis

Individual steps from `shiver` can be run with stand-alone command line tools, for ease of reapplication elsewhere. For example `CorrectContigs.py` is run with a file of contigs and a file of their `BLASTN` hits to some set of references, and corrects the contigs by cutting, trimming and reverse complementing where needed. Also included in `shiver` are command-line tools for easy analysis and modification of sample output without rerunning the whole pipeline:

- Two parameters specified in the configuration file are a minimum coverage required to call a base (below this coverage, the character '`?`' is used) and a larger minimum coverage required to use upper case instead of lower, as an easy signal of increased confidence. (Note that decreasing these parameters will, in general, allow bases to be called at more positions, giving a longer consensus. However there is a trade-off: where there are fewer reads, the effect of contaminant reads on the consensus may be greater.) To regenerate a consensus with new values of these parameters, `CallConsensus.py` can be run on a sample's base frequencies file. To regenerate a coordinate-translated version of this consensus for the global alignment (of all consensuses produced by `shiver`), `TranslateSeqForGlobalAln.py` can be run on the consensus.

- Another parameter in the configuration file is the minimum read *identity* – the fraction of bases in the read which are mapped and agree with the reference – required for a read to be considered mapped, and so retained in the BAM file. If you wish to increase this after completion of `shiver`, reads with an identity below your new higher threshold can be discarded by running `RemoveDivergentReads.py` on a BAM file. Running `shiver_reprocess_bam.sh` on the resulting BAM file (or indeed any BAM file) implements just the last steps in `shiver`, namely generating pileup, calculating the base frequencies, and calling the consensus.

- `FindNumMappedBases.py` calculates the total number of mapped bases in a BAM file (where read length is constant this equals the number of mapped reads multiplied by read length, minus the total length of sequence clipped from reads), optionally binned by read identity. In the absence of mapped contaminant reads, and all else being equal, mapping to a reference which is closer to the true consensus should map more bases and mapped reads should have higher identities.

- `FindClippingHotSpots.py` counts, at each position in the genome, the number and percentage of reads that are clipped from that position to their left or right end. Having many such reads is a warning sign of the kind of biased loss of information shown in Figure 2B.

- `FindSubSeqsInAlignment.py` finds the location of specified sub-sequences in an alignment (allowing for gaps).

- `LinkIdentityToCoverage.py` finds, for each different coverage encountered when considering all positions in a BAM file, the mean read identity at such positions. The mean read identity tends to be lower at positions of low coverage due to a background of contaminant reads, which differ from the reference by virtue of being contamination, but which are nevertheless similar enough to be mapped. Quantifying the decline in identity at low coverage helps inform what coverage threshold is appropriate for a given data set.

- `AlignMoreSeqsToPairWithMissingCoverage.py` allows more sequences to be added to a pairwise alignment in which one sequence contains missing coverage (such as a consensus and its reference), correctly maintaining the distinction between gaps (indicating a deletion) and missing coverage.

- `AlignBaseFreqFiles.py` aligns not two sequences, but two of the csv-format base frequency files output by `shiver`. Optionally a similarity metric is calculated at each position in the alignment, between 0 (no agreement on which bases/gaps are present) and 1 (perfect agreement on which

bases/gaps are present and on their proportions). This allows comparison not just of consensus sequences between two samples but also of minority variants.

- `ConvertAlnToColourCodes.py` converts each base in a sequence alignment into a colour code indicating agreement with the consensus and indels; `AlignmentPlotting.R` takes such colour codes and visualises the alignment. These two scripts were used to produce the plots of Supplementary Information sections 4 and 5.
- `QuantifyPairwiseIndels.py` considers all possible pairs of sequences in an alignment and calculates the sizes and positions of relative indels (i.e. ignoring positions at which both have a gap). It was used to make Figure 3.
- Finally some simple tools for convenience: `FindSeqsInFasta.py` extracts named sequences from a fasta file, with options including gap stripping, returning only windows of the sequences, and inverting the search; `PrintSeqLengths.py` prints sequence lengths with or without gaps; `SplitFasta.py` splits a fasta file into one file per sequence therein.

References

[1] A. Abecasis, A. Vandamme, and P. Lemey, HIV Sequence Compendium 2006/2007 (2007).

[2] K. McElroy, T. Thomas, and F. Luciani, Microbial Informatics and Experimentation **4**, 1 (2014).

[3] Geneious version 7.1 created by Biomatters. Available from http://www.geneious.com .
