# Supplementary material for: Easy and accurate reconstruction of whole HIV genomes from short-read sequence data with shiver
Source: Virus Evol. 2018 May 18;4(1):vey007. doi: 10.1093/ve/vey007 (PMC5961307; doi:10.1093/ve/vey007)
Supplement: Supplementary Data [file vey007_supp.zip › SupplementaryTableS1_formatted.pdf]

Supplementary Table S1: statistics for the processed samples. In the 'Notes' column: 'QC failure' indicates those samples that failed QC checks for assembly in the IVA publication; 'Contig correction' indicates those samples specifically chosen as examples of the need for shiver's correction of structural problems in contigs; 'Lane differences' indicates the sample chosen to illustrate differences in assembly output for technical replicate samples (i.e. the contigs for the two Hiseq lanes for this sample).

Sample	Miseq (M) or Hiseq (H) data	Closest identified real reference	shiver consensus length	Extra length in shiver consensuses (c.f. real reference consensus)	Extra length in shiver consensuses (c.f. real reference consensus) at the ends	Extra length in shiver consensus (c.f. real reference consensus) internally
ERR732065	M	B.AU.87.MBC925.AF042101	8198	174	0	174
ERR732066	M	B.DK.01.CTL_035.EF514710	7354	306	193	113
ERR732067	M	02_AG.GH.97.97GH_AG1.AB049811	5767	-54	0	-54
ERR732068	M	B.US.86.5096_86.AY835749	4561	2	0	2
ERR732069	M	B.US.00.THRO_TF1.JN944930	5695	54	0	54
ERR732070	M	A1.KE.04.04KE354207V3.KT022363	8077	410	289	121
ERR732071	M	11_cpx.CM.04.1230_24.KP718938	8230	471	12	459
ERR732072	M	B.KR.92.HP_10_02SHJ8_6986.KJ140255	8072	220	79	141
ERR732073	M	C.ZA.03.03ZASK107B1.DQ056410	9018	84	70	14
ERR732074	M	C.TZ.08.707010457_CH457.w8.KC156220	9048	86	0	86
ERR732076	M	C.ZA.99.99ZALT21.EU293446	9053	81	1	80
ERR732077	M	C.ZA.03.03ZASK107B1.DQ056410	9053	243	70	173
ERR732078	M	C.ZA.99.99ZALT21.EU293446	9053	78	0	78
ERR732079	M	C.ZA.03.03ZASK107B1.DQ056410	9053	283	70	213
ERR732080	M	C.ZA.03.03ZASK107B1.DQ056410	9029	189	70	119
ERR732081	M	C.ZA.03.03ZASK107B1.DQ056410	9023	185	70	115
ERR732082	M	C.IN.00.DEMC00IN008.KP109483	9038	258	185	73
ERR732083	M	B.TW.94.TWCYS_LM49.AF086817	9002	74	0	74
ERR732085	M	B.KR.93.HP_17_02LSP11_2268.KJ140262	9000	230	120	110
ERR732086	M	BF1.BR.10.10BR_RJ075.KT427651	9002	140	59	81
ERR732087	M	BF1.BR.10.10BR_RJ075.KT427651	9002	149	59	90
ERR732088	M	C.ZM.02.02ZMBC.AB254149	9027	1	0	1
ERR732089	M	B.KR.93.HP_17_02LSP11_2268.KJ140262	9005	173	121	52
ERR732090	M	B.KR.93.HP_17_02LSP11_2268.KJ140262	9002	132	0	132
ERR732091	M	01_AE.GB.10.Donor_N094_20_Month.KP873161	9048	18	3	15
ERR732092	M	01_AE.GB.10.Donor_N094_20_Month.KP873161	9030	4	3	1
ERR732093	M	B.KR.04.04WK7_HIV_1_wk.DQ295194	7312	86	-1	87
ERR732094	M	C.ZA.03.03ZASK107B1.DQ056410	9017	88	70	18
ERR732095	M	C.ZA.03.03ZASK107B1.DQ056410	9019	92	73	19
ERR732096	M	C.ZA.03.03ZASK107B1.DQ056410	9017	80	70	10
ERR732097	M	C.ZA.03.03ZASK107B1.DQ056410	9045	108	70	38
ERR732098	M	B.KR.93.HP_17_02LSP11_2268.KJ140262	9020	311	0	311
ERR732099	M	B.KR.93.HP_17_02LSP11_2268.KJ140262	9020	303	80	223
ERR732100	M	01_AE.GB.10.Donor_N094_20_Month.KP873161	9030	6	3	3
ERR732101	M	01_AE.GB.10.Donor_N094_20_Month.KP873161	9036	0	3	-3
ERR732102	M	C.TZ.08.707010457_CH457.w8.KC156220	9014	34	0	34
ERR732103	M	B.KR.93.HP_17_02LSP11_2268.KJ140262	9000	223	0	223
ERR732104	M	C.ZA.99.99ZALT21.EU293446	9029	56	0	56
ERR732105	M	C.ZA.99.99ZALT21.EU293446	9045	74	0	74
ERR732106	M	B.US.03.933384.KT124807	8961	447	374	73
ERR732107	M	B.US.06.06US_SAJ_C166_SG.JF689864	8967	608	380	228
ERR732108	M	B.US.13.862898.KT124796	9010	385	372	13
ERR732109	M	B.US.07.HIV_US_BID_V4516_2007.JQ403096	4559	293	293	0
ERR732110	M	B.AU.95.C24.AF538304	9037	334	115	219
ERR732111	M	B.CY.05.CY124.FJ388933	9064	1262	1077	185
ERR732112	M	B.JP.x.DR1712.AB604946	8127	80	0	80
ERR732113	M	B.KR.93.HP_17_02LSP11_2268.KJ140262	7353	207	0	207
ERR732114	M	B.KR.93.HP_17_02LSP11_2268.KJ140262	9008	299	0	299
ERR732115	M	B.KR.93.HP_17_02LSP11_2268.KJ140262	7957	501	232	269
ERR732116	M	B.KR.93.HP_17_02LSP11_2268.KJ140262	8999	270	80	190
ERR732117	M	01_AE.GB.10.Donor_N094_20_Month.KP873161	5004	53	3	50
ERR732118	M	01_AE.GB.10.Donor_N094_20_Month.KP873161	9070	65	22	43
ERR732119	M	C.ZA.05.05ZASK245B1.DQ369982	7355	145	70	75
ERR732120	M	B.US.87.5113_87.AY835758	7351	131	0	131
ERR732121	M	B.US.87.5113_87.AY835758	7351	131	0	131
ERR732122	M	B.US.04.ES8_43.EF363126	7328	216	-1	217
ERR732123	M	B.UY.99.99UY_TRA0177.JN235965	7763	596	299	297

Sample	Miseq (M) or Hiseq (H) data	Closest identified real reference	shiver consensus length	Extra length in shiver consensuses (c.f. real reference consensus)	Extra length in shiver consensuses (c.f. real reference consensus) at the ends	Extra length in shiver consensus (c.f. real reference consensus) internally
ERR732124	M	B.KR.05.05YJN2.JQ316134	1911	0	0	0
ERR732126	M	B.US.07.07US_SAJ_C161_H1.JF689883	7346	379	298	81
ERR732127	M	B.US.11.ES22_27.KF384808	1910	0	0	0
ERR732128	M	B.US.08.HIV_US_BID_V4489_2008.JQ403094	7345	492	293	199
ERR732129	M	B.GB.05.MM43d368_GN1.HM586209	9023	364	381	-17
ERR732130	M	B.GB.05.MM43d368_GN1.HM586209	9042	382	382	0
ERR732131	M	B.GB.05.MM43d368_GN1.HM586209	9022	377	381	-4
ERR732132	M	B.GB.05.MM43d368_GN1.HM586209	9020	365	381	-16
17621_3_80	H	0107.CN.07.JL070032.KC990127	8993	168	78	90
17653_3_25	H	B.JP.x.JRC65B.AB565502	9007	73	0	73
17653_3_36	H	B.FR.11.DEMB11FR001.KF716496	8932	253	257	-4
17653_3_56	H	02_AG.CM.01.01CM_0002BBY.AY371122	8994	643	638	5
17653_3_62	H	09_cpx.SN.95.95SN7808.AY093604	9080	454	336	118
17653_3_64	H	22_01A1.CM.01.01CM_0001BBY.AY371159	9057	712	647	65
17653_3_72	H	B.US.11.CP7_2B.KF384805	8999	158	89	69
17653_3_74	H	B.AU.86.MBC200.AF042100	9031	19	0	19
17654_3_46	H	B.YE.02.02YE508.AY795905	9026	327	299	28
17654_3_71	H	02_AG.CM.02.02CM_4082STN.AY371141	9042	640	638	2
17654_3_72	H	B.JP.98.DR1120.AB480698	9071	140	3	137
17654_3_78	H	B.KR.04.04KMK5.JQ316126	9046	205	92	113
17795_3_40	H	B.AU.86.MBC200.AF042100	9026	1	0	1
17796_3_1	H	14_BG.ES.05.X1870.FJ670522	9076	104	48	56
17796_3_29	H	B.DE.04.963987.KT124812	8971	361	293	68
17796_3_30	H	B.DE.86.D31.U43096	8966	61	0	61
17796_3_35	H	B.US.07.07US_SAJ_C166_MS.JF689886	9009	351	304	47
18209_3_31	H	C.ZA.03.03ZASK107B1.DQ056410	9039	110	70	40
18209_3_36	H	C.ZA.04.SK133B1.AY772698	8984	496	73	423
18209_3_38	H	B.ES.09.DEMB09ES007.KC473841	9003	145	137	8
19561_3_127	H	C.ZA.03.SK041B1.AY772693	9044	218	122	96
19562_3_109	H	01_AE.VN.97.97VNAG218.FJ185255	9041	299	257	42
19562_3_2	H	B.US.07.HIV_US_BID_V3120_2007.JQ403078	9014	369	293	76
19562_3_30	H	C.ZA.07.705010162_CH162.mo6.KC156115	8971	8	0	8
19562_3_31	H	B.JP.08.NMC104_clone_01.AB731663	9018	140	3	137
19562_3_46	H	B.US.x.AC_16_0_Days_Consen_fa.DQ127537	8904	370	300	70
19562_3_50	H	B.US.85.5077_85.AY835769	9057	70	0	70
19562_3_51	H	B.US.x.CR0192W.FJ469704	9040	315	275	40
19562_3_6	H	D.KE.11.DEMD11KE003.KF716476	9046	400	150	250
19893_3_71	H	01_AE.TH.05.05TH342968.JN248342	9018	340	310	30
19960_3_11	H	B.KR.92.HP_10_02SHJ8_6986.KJ140255	9032	221	104	117
19960_3_116	H	B.GB.x.MANC.U23487	9003	30	0	30
19960_3_119	H	B.FR.83.HXB2_LAI_IIIB_BRU.K03455	9051	87	0	87
19960_3_12	H	B.US.03.CR0154X.FJ469701	8980	309	281	28
19960_3_146	H	B.US.06.502_0346_wg02.JF320097	9026	378	293	85
19960_3_15	H	12_BF.UY.99.URTR23.AF385934	8978	9	0	9
19960_3_16	H	B.US.05.05US_SAJ_NVS12.JF689852	9046	411	309	102
19960_3_17	H	B.BR.04.BREPM1066.FJ195090	9028	64	49	15
19960_3_18	H	B.CY.08.CY226.JF683775	9002	962	298	664
19960_3_22	H	02_AG.DE.09.701114.KT124792	9120	429	309	120
19960_3_28	H	17_BF.BO.02.BO02_BOL119.EU581827	8948	293	296	-3
19960_3_40	H	A1.KE.99.KSM4021.AF457075	8986	690	298	392
19960_3_44	H	B.DE.86.HAN.U43141	9002	96	46	50
19960_3_49	H	B.TH.04.04TH803686.JN248333	9005	327	288	39
19960_3_6	H	BC.CN.07.jx070017.KF250384	8962	374	293	81
19960_3_70	H	BC.BR.92.92BR023.HM100716	9001	13	0	13
19960_3_9	H	B.KR.05.05CSR3.DQ837381	9038	155	38	117
20004_3_146	H	B.US.06.502_0346_wg02.JF320097	8951	339	294	45
20004_3_155	H	A1D.KE.06.06KE894822V7.KT022417	9020	517	285	232
20004_3_56	H	B.US.00.ES1_20.EF363123	8994	41	0	41
**minimum**	N/A	N/A	1910	-54	-1	-54
**median**	N/A	N/A	9009	205	70	73
**mean**	N/A	N/A	8535.7	239.4	143.2	96.2
**maximum**	N/A	N/A	9120	1262	1077	664

Sample	Number of bases called differently with higher coverage mapping to the shiver reference than to the real reference	Number of bases called differently with higher (or equal) coverage mapping to the real reference than to the shiver reference	Number of positions where at least one of the corrected contigs agrees with the shiver consensus	Number of positions where all corrected contigs disagree with the consensus
ERR732065	2	0	8142	7
ERR732066	1	0	7332	0
ERR732067	5	2	4911	5
ERR732068	1	0	4536	3
ERR732069	4	0	5632	20
ERR732070	16	0	8016	28
ERR732071	26	0	8178	13
ERR732072	42	4	7992	40
ERR732073	51	1	8962	56
ERR732074	53	3	9019	41
ERR732076	9	0	9049	4
ERR732077	19	0	9053	0
ERR732078	10	0	9053	0
ERR732079	20	1	9046	7
ERR732080	19	0	9020	9
ERR732081	32	0	8958	7
ERR732082	14	0	9025	13
ERR732083	27	9	8987	15
ERR732085	40	2	7029	44
ERR732086	16	0	8989	13
ERR732087	7	0	8998	4
ERR732088	52	0	9003	24
ERR732089	43	1	8992	13
ERR732090	57	6	8981	21
ERR732091	23	2	9019	29
ERR732092	42	4	8996	28
ERR732093	5	0	4867	2
ERR732094	30	2	8964	55
ERR732095	38	0	8914	106
ERR732096	31	1	8974	43
ERR732097	38	1	8994	59
ERR732098	2	0	9018	2
ERR732099	13	0	9019	1
ERR732100	23	2	9016	14
ERR732101	15	18	9004	32
ERR732102	55	1	8980	34
ERR732103	37	0	8827	14
ERR732104	25	0	9019	10
ERR732105	15	0	9040	13
ERR732106	19	0	8922	54
ERR732107	4	0	8960	7
ERR732108	2	0	9004	6
ERR732109	0	1	4535	2
ERR732110	16	0	9019	18
ERR732111	8	0	9027	37
ERR732112	5	0	8093	2
ERR732113	2	0	7327	6
ERR732114	5	0	8971	37
ERR732115	3	0	7315	9
ERR732116	22	0	8974	25
ERR732117	12	2	4944	9
ERR732118	8	1	9057	13
ERR732119	11	0	7325	10
ERR732120	6	0	7335	6
ERR732121	8	0	7333	0
ERR732122	3	0	7169	5
ERR732123	6	0	7738	8

Sample	Number of bases called differently with higher coverage mapping to the shiver reference than to the real reference	Number of bases called differently with higher (or equal) coverage mapping to the real reference than to the shiver reference	Number of positions where at least one of the corrected contigs agrees with the shiver consensus	Number of positions where all corrected contigs disagree with the consensus
ERR732124	0	0	1888	0
ERR732126	6	0	7324	3
ERR732127	0	0	1857	30
ERR732128	6	0	6090	7
ERR732129	0	0	9022	1
ERR732130	0	0	9042	0
ERR732131	1	0	9005	21
ERR732132	2	4	8997	23
17621_3_80	41	0	8911	46
17653_3_25	18	0	9001	6
17653_3_36	6	0	8794	3
17653_3_56	6	0	8989	5
17653_3_62	19	0	9062	33
17653_3_64	12	0	9048	9
17653_3_72	7	0	8957	3
17653_3_74	23	0	9029	2
17654_3_46	18	0	9013	11
17654_3_71	28	1	9036	5
17654_3_72	28	0	9069	2
17654_3_78	5	0	9038	8
17795_3_40	31	24	9019	7
17796_3_1	6	0	9026	2
17796_3_29	50	3	6740	3
17796_3_30	23	23	7319	2
17796_3_35	34	1	8035	13
18209_3_31	50	11	9017	19
18209_3_36	21	0	8980	4
18209_3_38	12	0	8999	4
19561_3_127	10	0	9039	5
19562_3_109	9	0	9038	3
19562_3_2	17	0	9005	10
19562_3_30	8	0	8960	11
19562_3_31	13	0	9012	3
19562_3_46	18	1	8894	3
19562_3_50	14	0	9052	5
19562_3_51	13	0	9033	7
19562_3_6	18	0	9043	3
19893_3_71	10	0	9014	3
19960_3_11	14	0	9023	9
19960_3_116	8	0	8997	6
19960_3_119	13	0	9039	12
19960_3_12	8	1	8980	0
19960_3_146	19	0	8965	64
19960_3_15	5	0	8973	2
19960_3_16	20	0	9042	4
19960_3_17	11	0	9024	4
19960_3_18	32	0	8998	4
19960_3_22	0	1	9116	4
19960_3_28	5	0	8942	6
19960_3_40	10	0	8983	3
19960_3_44	21	0	8999	3
19960_3_49	15	0	8992	13
19960_3_6	18	0	8959	3
19960_3_70	20	0	8993	8
19960_3_9	11	0	9029	6
20004_3_146	12	0	8941	10
20004_3_155	4	0	9018	2
20004_3_56	4	0	8987	5
**minimum**	0	0	1857	0
**median**	13	0	8992	7
**mean**	16.8	1.2	8408.7	13.7
**maximum**	57	24	9116	106

Sample	Number of positions where at least one contig has a base and the shiver mapping failed to call a base	Number of positions where there is no contig coverage but the shiver consensus has a base	Notes
ERR732065	0	49	QC failure
ERR732066	0	22	QC failure
ERR732067	0	852	QC failure
ERR732068	0	22	QC failure
ERR732069	0	43	QC failure
ERR732070	0	33	QC failure
ERR732071	0	39	QC failure
ERR732072	0	41	QC failure
ERR732073	0	0	
ERR732074	0	0	
ERR732076	0	0	
ERR732077	0	0	
ERR732078	0	0	
ERR732079	0	0	
ERR732080	0	0	
ERR732081	0	58	
ERR732082	0	0	
ERR732083	0	0	
ERR732085	0	1927	
ERR732086	0	0	
ERR732087	0	0	
ERR732088	0	0	
ERR732089	0	0	
ERR732090	0	0	
ERR732091	0	0	
ERR732092	0	6	
ERR732093	0	2443	QC failure
ERR732094	0	4	
ERR732095	0	0	
ERR732096	0	0	
ERR732097	0	3	
ERR732098	0	0	
ERR732099	0	0	
ERR732100	0	0	
ERR732101	0	0	
ERR732102	0	0	
ERR732103	0	159	
ERR732104	0	0	
ERR732105	0	0	
ERR732106	0	0	
ERR732107	0	0	
ERR732108	0	0	
ERR732109	0	22	QC failure
ERR732110	0	0	
ERR732111	0	0	
ERR732112	0	32	QC failure
ERR732113	0	20	QC failure
ERR732114	0	0	
ERR732115	0	633	QC failure
ERR732116	0	0	
ERR732117	0	51	QC failure
ERR732118	0	0	
ERR732119	0	20	QC failure
ERR732120	0	10	QC failure
ERR732121	0	18	QC failure
ERR732122	0	154	QC failure
ERR732123	0	17	QC failure

Sample	Number of positions where at least one contig has a base and the shiver mapping failed to call a base	Number of positions where there is no contig coverage but the shiver consensus has a base	Notes
ERR732124	0	23	QC failure
ERR732126	0	19	QC failure
ERR732127	0	23	QC failure
ERR732128	0	1248	QC failure
ERR732129	0	0	
ERR732130	0	0	
ERR732131	0	0	
ERR732132	0	0	
17621_3_80	0	36	Contig correction
17653_3_25	0	0	
17653_3_36	0	135	
17653_3_56	0	0	
17653_3_62	0	0	Contig correction
17653_3_64	0	0	
17653_3_72	0	39	
17653_3_74	0	0	
17654_3_46	0	5	
17654_3_71	0	1	
17654_3_72	0	0	
17654_3_78	0	0	
17795_3_40	0	0	Contig correction
17796_3_1	0	48	Contig correction
17796_3_29	0	2228	Contig correction
17796_3_30	0	1645	Lane differences
17796_3_35	0	967	Contig correction
18209_3_31	0	6	Contig correction
18209_3_36	0	0	Contig correction
18209_3_38	0	0	Contig correction
19561_3_127	0	0	
19562_3_109	0	0	
19562_3_2	0	0	
19562_3_30	0	0	
19562_3_31	0	3	
19562_3_46	0	7	
19562_3_50	0	0	
19562_3_51	0	0	
19562_3_6	0	0	
19893_3_71	0	1	
19960_3_11	0	0	
19960_3_116	0	0	
19960_3_119	0	0	
19960_3_12	0	0	
19960_3_146	0	0	
19960_3_15	0	3	
19960_3_16	0	0	
19960_3_17	0	0	
19960_3_18	0	0	
19960_3_22	0	0	
19960_3_28	0	0	
19960_3_40	0	0	
19960_3_44	0	0	
19960_3_49	0	0	
19960_3_6	0	0	
19960_3_70	0	0	
19960_3_9	0	3	
20004_3_146	0	0	
20004_3_155	0	0	
20004_3_56	0	2	
**minimum**	0	0	
**median**	0	0	
**mean**	0.0	114.1	
**maximum**	0	2443	
